# Behavioral changes before lockdown and decreased retail and recreation mobility during lockdown contributed most to controlling COVID-19 in Western countries

**DOI:** 10.1186/s12889-021-10676-1

**Published:** 2021-04-06

**Authors:** Koen Deforche, Jurgen Vercauteren, Viktor Müller, Anne-Mieke Vandamme

**Affiliations:** 1 Emweb bv, Herent, Belgium; 2 Department of Microbiology, Immunology and Transplantation, Rega Institute for Medical Research, Clinical and Epidemiological Virology, Katholieke Universiteit Leuven, 3000 Leuven, Belgium; 3 Insitute of Biology, Eötvös Loránd University, Budapest, Hungary; 4 Center for Global Health and Tropical Medicine, Unidade de Microbiologia, Instituto de Higiene e Medicina Tropical, Universidade Nova de Lisboa, Lisbon, Portugal

**Keywords:** COVID19, Mobility, Reproduction number, Non-pharmaceutical interventions

## Abstract

**Background:**

The COVID-19 pandemic has prompted a lockdown in many countries to control the exponential spread of the SARS-CoV-2 virus, hereby reducing the time-varying basic reproduction number (*R*_*t*_) to below one. Governments are looking for evidence to balance the demand of their citizens to ease some of the restriction, against the fear of a new peak in infections. In this study, we wanted to quantify the relative contribution of mobility restrictions, and that of behavioral changes that occurred already before the lockdowns, on the reduction of transmission during lockdowns in Western countries in early 2020.

**Methods:**

Incidence data of cases and deaths from the first wave of infections for 35 Western countries (32 European, plus Israel, USA and Canada) were analyzed using epidemiological compartment models in a Bayesian framework. Mobility data was used to estimate the timing of changes associated with a lockdown, and was correlated with estimated reductions of *R*_*t*_.

**Results:**

Across all countries, the initial median estimate for *R*_*t*_ was 3.6 (95% IQR 2.4–5.2), and it was reduced to 0.78 (95% IQR 0.58–1.01) during lockdown. 48% (18–65%) of the reduction occurred already in the week before lockdown, with lockdown itself causing the remaining drop in transmission. A lower *R*_*t*_ during lockdown was independently associated with an increased time spent at home (0.21 per 10% more time, *p* < 0.007), and decreased mobility related to retail and recreation (0.07 per 10% less mobility, *p* < 0.008).

**Conclusions:**

In a Western population unaware of the risk, SARS-CoV-2 can be highly contagious with a reproduction number *R*_*0*_ > 5. Our results are consistent with evidence that recreational activities (including restaurant and bar visits) enable super-spreading events. Exiting from lockdown therefore requires continued physical distancing and tight control on this kind of activities.

**Supplementary Information:**

The online version contains supplementary material available at 10.1186/s12889-021-10676-1.

## Background

In February–March 2020, it became clear that the COVID-19 pandemic had been introduced in Europe and Northern America [1] and governments and societies responded in various ways to slow down the spread of the virus. In the containment phase, testing, contact-tracing and individual quarantine of (suspected) infected individuals are crucial to prevent a widespread epidemic. If this fails, mitigation measures consist mainly of mobility restrictions and encouraged or mandated physical distancing to everyone regardless of symptoms or testing results (jointly called lockdown).

Failing containment, most European countries managed to curb the epidemic by May through lockdown measures (timeline and details of measures in the different countries [2]), however without fully understanding which of the different measures had the strongest impact. Because of economic and social implications of a lockdown, governments have been cautiously relaxing measures while trying to avoid a new peak by closely following the effect on the epidemics through testing and monitoring hospitalizations and casualties [3]. Coming out of lockdown entails a return to containment measures, with testing, contact tracing and quarantine again being crucial.

To understand the evolution of the COVID-19 epidemics, to estimate the impact of testing strategies and interventions and to estimate key epidemiological parameters, epidemiological models have been widely used [4–8]. Epidemic spread is determined by both biological properties of the virus as well as the behavior of the host population, and this is reflected in the epidemiological parameters. One important parameter, the time-varying basic reproductive number *R*_*t*_, determines whether an epidemic is exponentially growing (*R*_*t*_ > 1) or declining (*R*_*t*_ < 1). The goal of both containment and lockdown measures is to bring *R*_*t*_ below 1 by changing the behavior of the infected, respectively, susceptible population through reducing their contact networks.

In early 2020, the population in Western countries started to develop an increased awareness as soon as media reported on the epidemic unfolding in Lombardy (Italy), and this led to a gradual response ranging from voluntary physical distancing to government imposed mandatory self-isolation upon showing symptoms, banning of public events, and encouraging physical distancing, with ultimately a drastic lockdown when many businesses and schools were closed and mobility was severely restricted [9]. The diversity of measures and their timing gives us the opportunity to investigate which responses were correlated with a better outcome. However, reliable data quantifying the impact on human behavior of various country-dependent specific lockdown measures is currently lacking.

Attempts to create such data sets face challenges such as variation in compliance to imposed measures, and the unknown level of measures taken by individuals or organizations in absence or anticipation of government regulations. Rather than an account of the ordered measures in each country, we used mobility changes extracted from smartphone location data as an objective indication and quantification of the lockdown (Suppl 1).

To quantify the impact of various aspects of non-pharmaceutical interventions in response to the epidemic, we estimated in this study the change in transmission over time using incidence data of deaths and diagnosed cases in 35 countries (ECDC data downloaded on 6 June 2020, see Suppl 2), differentiating between the effect of measures that preceded the lockdown, and the effect of the lockdown itself. The change in transmission during lockdown was further analyzed by testing for correlation with the estimated reductions in six mobility categories. Only data up to 60 days into the lockdown (up to about mid-May in European countries) were used to avoid the confounding effect of the gradual lifting of restrictions, which started in many European countries in May.

## Methods

**Fig. 1:**
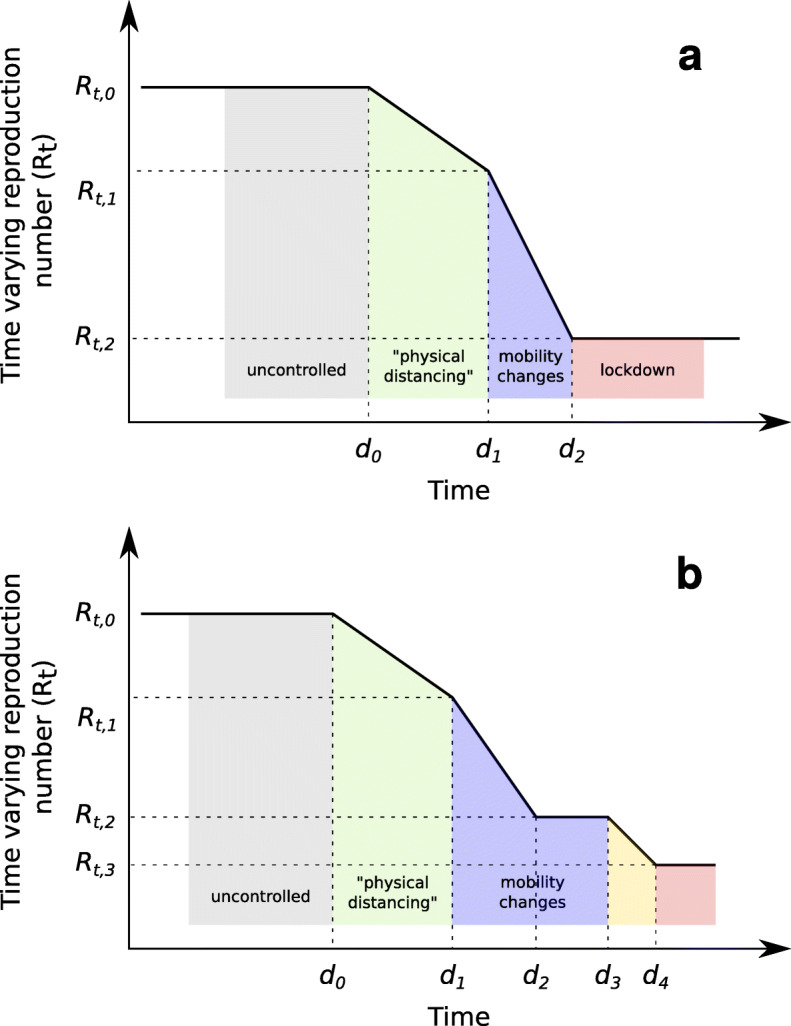
Model for changes of the time-varying reproduction number *R*_*t*_ as a piece-wise linear function. Dates *d*_*1*_ and *d*_*2*_ were estimated from mobility data. Date *d*_*0*_ and values for *R*_*t,0*_ – *R*_*t,2*_ were estimated from incidence data on diagnosed cases and deaths. a. *model-2* used for all countries; b. *model-3p* additionally used for Slovakia, which allowed an extra change during lockdown with dates *d*_*0*_, *d*_*3*_ and *d*_*4*_, and values for *R*_*t,0*_ – *R*_*t,3*_ estimated from incidence data on diagnosed cases and deaths

In order to obtain comparable estimates of *R*_*t*_, a simple SEIR compartment model was used, with epidemiological parameters that model biological properties (latent period, infectious period duration), or that were of less importance to the study (infection fatality rate), kept constant. Parameters that model transmission rates were allowed to change from an initial estimated value *R*_*t,0*_ during a transition period, which was also estimated from the data, to *R*_*t,1*_ until the day that mobility changes started, and then to *R*_*t,2*_ during the lockdown identified on the basis of mobility data, using a piecewise linear model (Fig. 1a and Methods). Although the introduction of mobility restrictions coincided with other measures including behavioral changes, in the manuscript we use “mobility changes” to indicate the combination of mobility restrictions and behavioral changes that happened at the time of “lockdown”, versus “behavioral changes” that we use for changes that have led to a reduction in transmission prior to the lockdown.

**Fig. 2:**
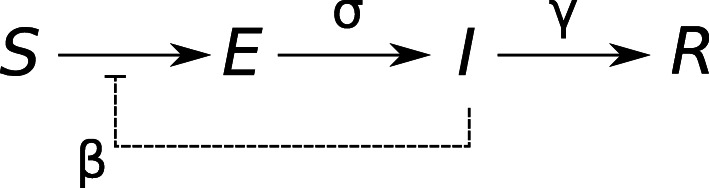
Structure of a standard SEIR compartment model with four compartments: susceptible (S), exposed (E), infectious (I), and removed (recovered or deceased, R). Susceptible individuals become latently infected by infectious individuals, with transmission rate β. Latently infected become infectious at rate σ. Infectious people are removed at rate γ

The following equations describe the dynamics of individuals in each of the four compartments of a standard SEIR model (see Fig. 2):

$$ \frac{dS}{dt}=\frac{-\beta IS}{N} $$

$$ \frac{dE}{dT}=\frac{\beta IS}{N}-\sigma E $$

$$ \frac{dI}{dt}=\sigma E-\gamma I $$

$$ \frac{dR}{dt}=\gamma I $$

The differential equations of the SEIR compartment models were numerically integrated using deSolve [10]. The duration of the latent period *T*_lat_ = 1/σ was fixed to 3 days, and the infectious period *T*_inf_ = 1/γ to 5.2 days [11, 12]. Sensitivity of the results to other parameter values (*T*_lat_ = 2 or 4 days, and *T*_inf_ = 4.5 or 5.9 days) were conducted.

The dates *d*_*1*_ and *d*_*2*_ which mark the transition period for mobility changes were estimated from mobility data reports [13], by fitting a step function with a linear transition period through the sum of mobility changes related to transit stations and workplaces (see Suppl 1). This was motivated by the adoption of teleworking as a measure in most countries, even in those (like Sweden) which had the lightest lockdown regimen. The value for β was modeled as a piece-wise linear function of time (Fig. 1a). To verify that the assumption that mobility changes leading to the lockdown were the final measure to control transmission, models were also estimated which allowed a further reduction in transmission during lockdown to a value *R*_*t,3*_ (assuming a prior distribution *R*_*t,3*_ − *R*_*t,2*_ = N(0, 0.3) expressing no change with respect to *R*_*t,2*_), with a linear transition between co-estimated dates *d*_*3*_ and *d*_*4*_ (see Fig. 1b). The statistical support for these more complex models was evaluated using Deviance Information Criterion.

We wanted a model that does not require independent estimates for the timing or number of introductions per country. Instead, the models were seeded with an initial single exposed individual and it was assumed that the estimated date *d*_*0*_, marking the first change in value of β, was linked to a co-estimated threshold for cumulative deaths. This method had the benefit of avoiding any bias on estimated *R*_*0*_ values because of assumptions on introduction time and numbers, while being more efficient to sample than time of first infection.

To estimate the model parameters, incidence data of diagnosed cases and deaths was used (ECDC, https://opendata.ecdc.europa.eu/covid19/casedistribution/csv, accessed on 6 June), within a Bayesian framework. The daily incidence of diagnosed cases and deaths was compared to the predicted numbers using a negative binomial distribution, and assuming a Gamma distribution for infection-to-test and infection-to-death intervals. Taking advantage of the assumed timing of lockdown dates, the gamma distribution parameters (shape and scale) were co-estimated for each country (see Suppl 3). The posterior distributions of model parameters were estimated in R using MCMC with Metropolis coupling (20 chains at different temperatures) [14–17].

Suppl 3 Table 1 lists all parameters used in the models and during the estimation from data, with their values (either a constant, or a prior distribution for parameters that were estimated).

To correlate mobility changes in the different categories reported in Google Mobility reports [13], with the estimated reduction in transmission estimated from the incidence data, first we calculated the mean value in each of mobility category before date *d1* (before the start of reduction in mobility) and after *d2* (the start of the lockdown period). We then used univariate and multivariate linear models to estimate the effect of mobility changes on the reproduction rate of the epidemic, for each mobility category separately, and for all of them together. Collinearity was assessed by calculating covariance-inflation factors [18]. All data files, R scripts and analysis steps are described in Suppl 2. All data used for the analyses were obtained from published databases. No additional data were collected.

## Results

**Fig. 3:**
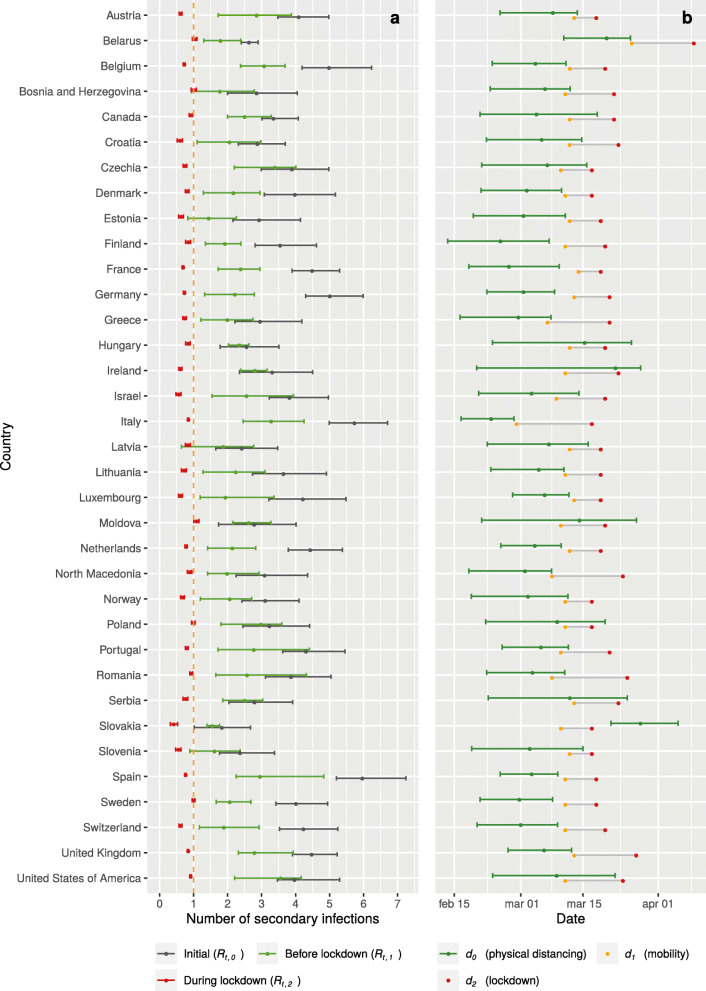
a. Posterior estimates of the initial basic reproduction numbers (*R*_*t,0*_), the reproduction number at start of lockdown (*R*_*t,1*_) and during lockdown (*R*_*t,2*_). b. Estimated median values (95% IQR) for *d*_*0*_ (date of first reduction in transmission, presumably due to physical distancing, estimated from incidence data of deaths and diagnosed cases); and lockdown transition start and end dates *d*_*1*_ and *d*_*2*_, estimated from mobility data (see also Suppl 1)

The estimated models fitted well the reported incidence data for most countries (see Suppl 4). The time between infection and reported death was estimated as a Gamma distribution with mean 26 days but with considerable variation between different countries (95% IQR 16–32, Suppl 3 Table 3). Across all countries, the median of posterior estimates for *R*_*t,0*_ was 3.6 (95% IQR 2.4–5.2) (see Fig. 3a, Suppl 3 Table 2). Before changes in mobility were observed (*d*_*1*_), the reproduction number was reduced to 2.2 (95% IQR 1.7–3.3). During lockdown, transmission was further reduced to 0.78 (95% IQR 0.58–1.01). Only for Belarus and Moldova, the median estimates of *R*_*t,2*_ were slightly above 1, and for Bosnia and Herzegovina, Poland and Sweden, the 95% CRI did not exclude a value of *Rt,*_*2*_ above 1, while all other countries had the *R*_*t,2*_ value below 1. Comparing the estimated reproduction number during lockdown *R*_*t,2*_ with the initial reproduction number *R*_*t,0*_ in each country, we found that 48% (18–65%) of the reduction occurred before lockdown, and the remaining 52% (35–82%) associated with mobility changes during lockdown.

**Fig. 4:**
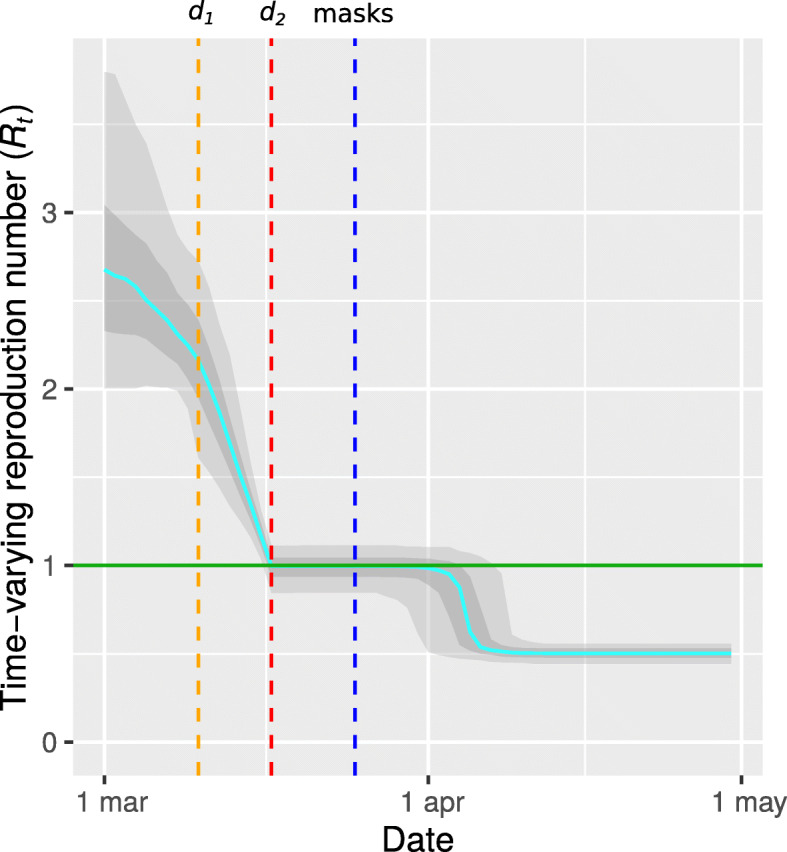
Estimated time-varying basic reproduction number *R*_*t*_ for Slovakia using a model that allowed an additional reduction of transmission rate at a co-estimated date during lockdown. The orange and red lines mark *d*_*1*_ and *d*_*2*_ as estimated from google mobility data. For reference, the date at which mandatory mask wearing was introduced (25 March) is indicated in blue

We estimated that the date *d*_*0*_ of the first decline in transmission preceded observed mobility changes by on average 6 days (95% IQR -6 – 12) (Fig. 3a). Although the semantic meaning given to *d*_*0*_ (first date of decline of *R*_*0*_, before mobility changes), assumed that it would be estimated before *d*_*1*_, this order was not enforced. For Slovakia, the estimated model placed this date around 27 March (95% IQR 21 March – 5 April), later than the dates *d*_*1*_ and *d*_*2*_ that mark the mobility changes period (10–17 March). At the same time, a suspiciously low value of 1.8 (95% IQR 1.0–2.7) for *R*_*t,0*_ was estimated. Both observations indicated that the assumption that mobility changes leading to the lockdown were the final measure to control transmission, was not applicable to data of Slovakia. We therefore re-estimated a model which allowed a further reduction in transmission to a value *R*_*t,3*_. This model estimated an additional reduction of *R*_*t*_ around 3 April (95% IQR 27 March – 8 April, Fig. 4), reducing transmission with 50% (95% IQR 36–59%) from 0.92 (95% IQR 0.81–1.14) to 0.50 (95% IQR 0.43–0.56). In further statistical analyses, the parameters estimated for this latter model were used only for Slovakia.

**Fig. 5:**
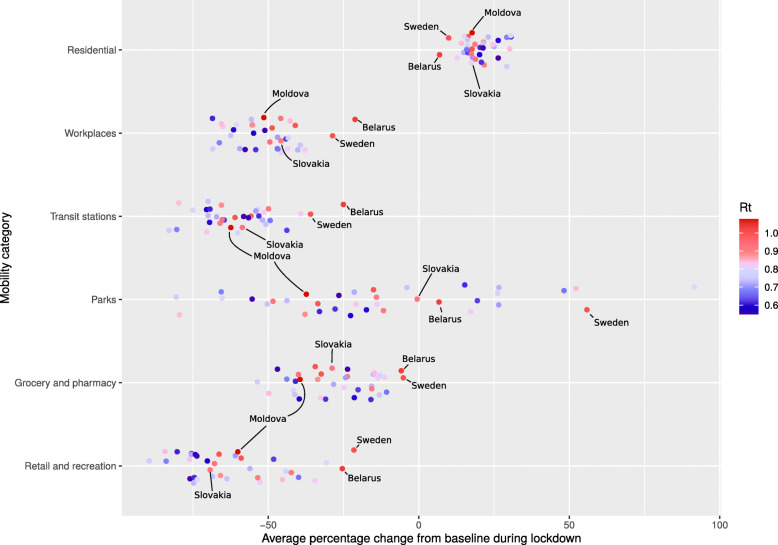
Average percentage change in mobility, compared to baseline, for six location Google mobility categories. Color reflects basic reproduction number *R*_*t*_ during lockdown. Retail and Recreation: restaurants, cafes, shopping centers, theme parks, museums, libraries, and movie theaters; Grocery and Pharmacy: grocery markets, food warehouses, farmers markets, specialty food shops, drug stores, and pharmacies; Parks: local parks, national parks, public beaches, marinas, dog parks, plazas, and public gardens; Transit Stations: public transport hubs such as subway, bus, and train stations; Workplaces: places of work; Residential: places of residence. To avoid overloading the Figure, only countries which were mentioned in the Results and Discussion in the context of the estimated effectiveness of their lockdown, were highlighted here

To investigate how mobility changes correlate with the reduction of *R*_*t*_ during lockdown, Google Mobility report data were used. These reports define the mobility change for six categories, compared to a baseline defined as the median mobility in January (Fig. 5). For all six mobility categories, mobility during lockdown was significantly different compared to mobility in the few weeks before lockdown (Wilcoxon paired test *p* < 0.01, data not shown). Mobility before lockdown was similar to the baseline with 95% IQR ranges of − 6 to 6%, with the exception of mobility related to parks which showed already a median increase of 10% (95% IQR 3–19%) compared to January. The latter may be expected since park visits are less common during the winter. Mobility changes for each category showed the same trend in all countries, again with the exception of mobility related to parks which was increased or decreased depending on the country (Fig. 5). As a consequence, each mobility category can explain a large part of the variation in *R*_*t*_, even in absence of a causal relation. To overcome this difficulty in identifying the causal factors driving the change, we correlated the final (lowest) reproduction number *R*_*t,2*_ during lockdown (rather than the change in *R*_*t*_) with variation in mobility changes during lockdown. Lower *R*_*t,2*_ values during lockdown were significantly associated with a larger mobility reduction related to retail, recreation, and workplaces, and a longer time spent in residential places (Fig. 5, Table 1). The associations of mobility reduction related to retail and recreation, and residential places remained significant in a multivariate model which explained 47% of variance (adjusted *R*_*2*_) of the *R*_*t,2*_ value during lockdown (*p* < 0.0008). Using this model, we estimated that, in 35 Western countries, reductions of mobility related to retail and recreation during lockdown caused a mean reduction of *R*_*t*_ of 0.45 (95% CI 0.13–0.76), or thus an average reduction of 20% (95% CI 6–34%) in transmission, and in addition more time spent in residential places (presumably instead of going to school or work) caused a mean reduction of *R*_*t*_ of 0.41 (95% CI 0.12–0.68) or thus an average reduction of 18% (95% CI 6–31%). Increased mobility related to parks may be an indicator of social restrictions in visiting friends or family, and explained up to 5% (95% CI 1–9%) of additional reduction in transmission.

**Table 1:** Univariate and multivariate associations of mobility changes during lockdown (per 10% mobility change) with the basic reproduction number R t,2 during lockdown. Mobility data related to workplaces were left out from the multivariate analysis since this variable was highly correlated with mobility data related to residential places and transit stations (variance-inflation factors > 8)

Variable	Univariate estimate		Multivariate estimate	
Retail and recreation	0.04 +/− 0.01	(*p* < 0.01)	0.07 +/− 0.02	(*p* < 0.008)
Grocery and pharmacy	0.01 +/− 0.02		− 0.02 +/− 0.03	
Parks	0.002 +/− 0.006		−0.021 +/− 0.009	(*p* < 0.03)
Transit stations	0.03 +/− 0.02		− 0.06 +/− 0.03	(*p* = 0.096)
Workplaces	0.05 +/− 0.02	(*p* < 0.02)		
Residential	− 0.11 +/− 0.04	(*p* < 0.009)	−0.21 +/− 0.07	(*p* < 0.007)
*R*_*t,1*_	0.04 +/− 0.05		0.06 +/− 0.04	

To verify that the relationship between mobility changes and *R*_*t,2*_ can also explain the large reduction in *R*_*t*_ as a result of the lockdown, a multivariate model that directly predicted the percentage change of *R*_*t*_ compared to *R*_*t,1*_ before lockdown, based on mobility changes before *d*_*1*_ and after *d*_*2*_, was also estimated (data not shown). This model used two data points per country (at date *d*_*1*_ and date *d*_*2*_) and had a high explanatory power (*R*^2^ = 0.94) but as expected suffered from high collinearity (with covariation-inflation factors over 100) and was therefore not reliable in attributing and quantifying the change of *R*_*t*_ to specific mobility categories. Nevertheless, confirming the trends of the above findings, this model attributed an estimated 40% (95% CI 20–58%) and 29% (95% CI 13–44%) of the drop in R t during lockdown respectively to a reduction in mobility related to retail and recreation and staying at home (*p* < 10^− 3^).

**Fig. 6:**
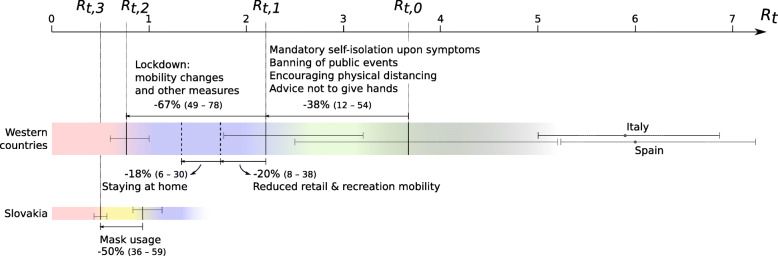
Summary of estimated contributions to the reduction of transmission in 35 Western countries. Initial basic reproduction numbers (*R*_*t,0*_), the reproduction number at start of lockdown (*R*_*t,1*_) and during lockdown (*R*_*t,2*_), and percentage reductions are shown as median values and 95% IQR. Estimates for individual countries (Italy, Spain and Slovakia) are shown as median posterior value and 95% cri

Our findings are summarized in Fig. 6 and trends were found to be robust to different assumptions of latent period duration (in the range of 2 to 4 days), and different assumptions of generation time (in the range of 4.5 to 5.9 days), see Suppl 3.

## Discussion

By assuming that lockdown is associated with drastic mobility changes, the timing of the lockdown in each country was estimated from mobility data. Time-varying reproduction numbers were then estimated for 35 countries resulting in country-specific estimates for the initial reproduction number *R*_*t,0*_, a reproduction number *R*_*t,1*_ at the beginning of mobility changes, and a *R*_*t,2*_ during lockdown. Estimates for *R*_*t,0*_ are expected to vary depending on setting, methodology and assumptions on parameters (especially duration of infectious period and generation time) and assumptions on how the number may vary over time [7]. We find that our estimated values for *R*_*t,0*_ tend to be higher, and estimated values for *R*_*t,1*_ lower, compared to estimates in other studies for *R*_*0*_ of around 2.7. Our estimates for *R*_*t,0*_ are similar to *R*_*0*_ estimates obtained using models that also consider interventions that preceded a full lockdown [9]. For the initial reproduction number *R*_*t,0*_, substantial variation between countries was estimated. In particular, in European countries with the earliest outbreaks, high values of 5.7 (95% cri 5.0–6.7) for Italy and 6.0 (95% cri 5.2–7.2) for Spain were found, reflecting the high level of transmission of this virus in a Western population that is mostly unaware of the risk.

During a period of on average 6 days before lockdown, less invasive measures such as the mandatory self-isolation upon showing symptoms, banning of public events, and encouraging physical distancing may have contributed to a decline in transmission (there was no substantial decline in mobility during this period). We found that about half of the reduction in transmission happened during this period before lockdown, possibly as a consequence of behavioral changes which may include increased hand hygiene, physical distancing and encouraged or mandated self-isolation and measures such as prohibition of public events. This is in contrast with the conclusions of Flaxman et al [9] who attributed 81% (75–87%) of the reduction in transmission to lockdown, and only limited contributions of other measures. However, for one country (Sweden), they assumed no lockdown and attributed over 80% of the reduction in transmission to the banning of public events, which is at odds with the finding that this same measure had a negligible contribution (< 5%) in other countries. Furthermore, their results were found to be highly sensitive to the assumed order of interventions [19]. In our analyses, the estimated value for *R*_*t,2*_ for the epidemic in Sweden was found to be relatively high (1.00, 95% cri 0.96–1.04), but this value was equally well explained based on mobility changes. This suggests that, although not enforced, in practice the Swedish population reacted similarly to the epidemic, but to a lesser extent, compared to other Western countries.

For several countries (Finland, France, Greece, Italy, and Sweden) the initial reduction in transmission was estimated near the end of February and might have been a result of awareness raised by the discovery of the first European cluster around that time in Lombardy, Italy. Three countries with a high estimated *R*_*0*_ > 5 (Spain, Italy, and Belgium) were also the countries in Europe with the highest excess mortality during this period (https://www.euromomo.eu/graphs-and-maps), suggesting that the high mortality rate in these countries may in part be explained by a high transmission rate in these countries even before awareness was raised by the discovery of the outbreak in Lombardy beginning of March, and thus not necessarily because of a too late or less effective reaction in response. With regard to Belgium, several lines of evidence suggest that the virus had already entered the country before the end of February [20].

The assumed time interval distribution between infections and deaths is essential to correlate changes in transmission rates with incidence data of deaths. By associating the changes in transmission rate with changes in mobility data, parameters for Gamma distributions were coestimated for each country. The estimated median mean time (26 days) was comparable with earlier estimates (21 days) based on clinical data [9], but showed considerable variation between countries which may reflect differences in clinical and reporting practices.

To explore sensitivity of our findings to assumptions on the duration of the latent period and infectious period, sensitivity analyses were conducted. These analyses suggested that an incubation time of 2 days rather than the assumed 3 days is more realistic based on more robust associations with mobility data. Using an estimated incubation period of around 5 days [21], this also implying that the average period of presymptomatic transmission is 3 days.

For Slovakia, an additional reduction in transmission during lockdown was found to have occurred at the beginning of April, and this followed shortly after the introduction of mandatory face masks use in Slovakia on 25 March [22]. This would imply that using face masks reduced transmission by half. This finding may however be sensitive to a violation of model assumptions since for 10 other countries a significant (but smaller) reduction in transmission during lockdown was also estimated (Suppl 3 Table 4), seemingly uncorrelated to additional measures adopted in these countries.

Because most mobility restrictions coincided in time, it is challenging to identify those mobility changes that contributed most to the reduction in transmission. In our analysis, because of correlation, the potential effect of a lower mobility related to workplaces could not be disambiguated from the general effect of increased time spent in residential places. The fact that in addition to more time spent in residential places, a reduced mobility related to retail and recreation was significantly associated with *R*_*t*_ both in univariate and multivariate analyses, suggests that activities related to visiting malls, bars, restaurants, or museums are linked to increased transmission and releasing those mobility restrictions should be done with care since they may carry a high risk for reigniting the epidemic. This is in line with mounting evidence of transmission being promoted by (loud) speaking or singing (bars, choir [23]), and longer time spent in densely populated indoor locations with low air circulation (bars, restaurants, malls, events with mass gatherings [24]) [25, 26]. The remaining reduction of *R*_*t*_ during lockdown may still be related to mobility changes of other types of mobility currently not reported by Google (for example mobility related to schools) or would require further refinement of categories to become observable. Alternatively, other types of changes that coincided in time with these mobility changes could be responsible.

Despite the large impact of mobility restrictions and business closures on society, the implementation of certain measures may need to be maintained for a long time and should thus be as fine-grained and optimally chosen as possible. Therefore, the confirmation and quantification of how measures reduce transmission have been studied using diverse approaches in terms of data sets and methods [8, 19, 27–29]. Our results largely confirm findings of these other studies.

This study has several limitations. The use of a compartment model in conjunction with a piecewise linear model for *R*_*t*_ is necessarily an approximation, and more complicated temporal changes in transmission may have occurred in reality, but still it served the purpose of examining the impact of different measures over time. Epidemiological parameter estimates may have been impacted by shifts in age distribution of cases over time, given that COVID-19 has a distinct age risk profile, and also by variation in data accuracy between countries. By limiting the study to Western countries which coincided more or less in time, differences related to culture, climate, and google mobility data interpretation, may have been less of a confounding factor. Finally, the effect of the stringency of the mobility restrictions was not evaluated as it is hard to measure in an objective way.

## Conclusions

In a Western population unaware of the risk, exemplified by Italy and Spain being the initial European countries to have been hit by the epidemic, SARS-CoV-2 is highly transmissable with reproduction numbers *R*_0_ > 5. Behavioral changes and measures such as restricting large gatherings can already reduce transmission by half. Next to increased time spent at home, in general, the reduction of recreational activities (including restaurant and bar visits) was identified as contributing most to the reduction in transmission during lockdown. Our finding that nearly half of the reductions in transmission occurred before lockdown indicates the importance of behavioral patterns that are independent of state-imposed restrictions. This finding underlines the danger of ‘COVID-19 fatigue’ – the increased difficulty over time to maintain the behavioral routines that are needed to minimize the transmission of the virus. All efforts should be made to increase awareness of the importance of individual decisions and risk avoidance in the continued fight against the pandemic. Exiting from lockdown therefore requires continued physical distancing and tight control on circumstances that facilitate massive spread such as large gatherings especially indoors.

## Supplementary Information

**Additional file 1.** Lockdown period estimates.

**Additional file 2.** Data and analysis scripts

**Additional file 3.** Supplementary methods and results.

**Additional file 4.** Model predictions per country.

## Data Availability

More info on the methods used in this study and on the location of the datasets generated and/or analyzed is described in the supplementary material: Supplementary material 1 Lockdown period estimates Supplementary material 2 Data and analysis scripts Supplementaery material 3 Supplementary methods and results Supplementary material 4: model predictions per country Lockdown dates for each of the countries were estimated from Google COVID-19 Mobility reports (https://www.google.com/covid19/mobility/, Accessed: 2020-05-04). The source code and analysis files are hosted at: https://github.com/kdeforche/epi-mcmc
