# Supplementary material for: Behavioral changes before lockdown and decreased retail and recreation mobility during lockdown contributed most to controlling COVID-19 in Western countries
Source: BMC Public Health. 2021 Apr 6;21:654. doi: 10.1186/s12889-021-10676-1 (PMC8022318; doi:10.1186/s12889-021-10676-1)
Supplement: Supplementary file 1 — Additional file 1. Lockdown period estimates. [file 12889_2021_10676_MOESM1_ESM.pdf]

Supplementary material 1: Lockdown period estimates

Lockdown dates for each of the countries were estimated from Google COVID-19 Mobility reports (`https://www.google.com/covid19/mobility/`, Accessed: 2020-05-04). The breakdown of mobility parameters is as follows (see: `https://www.openriskmanagement.com/exploring_community_mobility_reports_using_opencpm/`):

Retail and Recreation: restaurants, cafes, shopping centers, theme parks, museums, libraries, and movie theaters

Grocery and Pharmacy: grocery markets, food warehouses, farmers markets, specialty food shops, drug stores, and pharmacies

Parks: local parks, national parks, public beaches, marinas, dog parks, plazas, and public gardens

Transit Stations: public transport hubs such as subway, bus, and train stations

Workplaces: places of work

Residential: places of residence

The lockdown was modeled as a transition period between two dates d_1 and d_2. These dates were estimated by fitting a stepfunction with a linear transition to % change mobility data of (transit stations + workplaces), using a least absolute value error function (to be less sensitive to outliers). The four parameters of the step function (d_1, d_2, v_1, and v_2) were estimated using `optim` in R (implemented in *data.R*, see Suppl 2).

To judge how the estimated values for d_1 and d_2 compared to the introduction of measures as countries went into lockdown, the documented measures for Belgium and neighbouring countries are listed here for reference (source: Wikipedia):

- *Belgium* (d_1 = March 12, and d_2 = March 20): a first set of measures were announced on March 12. These included closure of schools, discos, cafes and restaurants, and cancellation of public gatherings, and telework wherever possible. On March 19, further measures (and final) measures were put in place: closure of non-essential shops, non-essential travel was prohibited, gatherings banned, and penalties for companies and individuals to abide by the rules.

- *the Netherlands* (d_1 = March 12, and d_2 = March 19): between March 9 and March 10, gradually guidelines from recommendations to work from home and stop shaking hands, to bans on large events, were put in place for the province of North Brabant. On March 12, people in the entire nation were encouraged to work from home, and gatherings were banned. On March 15, schools, childcare centres, cafs, restaurants, sports clubs, saunas, sex clubs and coffeeshops were closed.

- *France* (d_1 = March 14, and d_2 = March 19): bans on gatherings were introduced between March 5 to 14, from 5000 down to 100 people. On March 15, closure of most public establishments were ordered. Only essential services (food shops, pharmacies, banks, newsagents, petrol/service stations) and all essential public services" were authorised to remain open. Between March 16 and March 24, further measures were introduced (school closings, religious gatherings, and all non-essential travel.

- *Germany* (d_1 = March 13, and d_2 = March 21): bans on gatherings were introduced on March 8. On March 13, 14 of the 16 German federal states decided to close schools and nurseries. On March 14, several states limited public activities (closing bars and other leisure venues). Between March 16 and March 22, further measures were taken by states (forbidding public gatherings of more than two people, closing of restaurants, hairdressers) and some states forbid also all non-essential travel.

- *Luxembourg* (d_1 = March 13, and d_2 = March 19): on March 15, closure of non-food shops, restaurants was mandated. On March 16, schools were closed. On March 20, public transport services were significantly reduced.

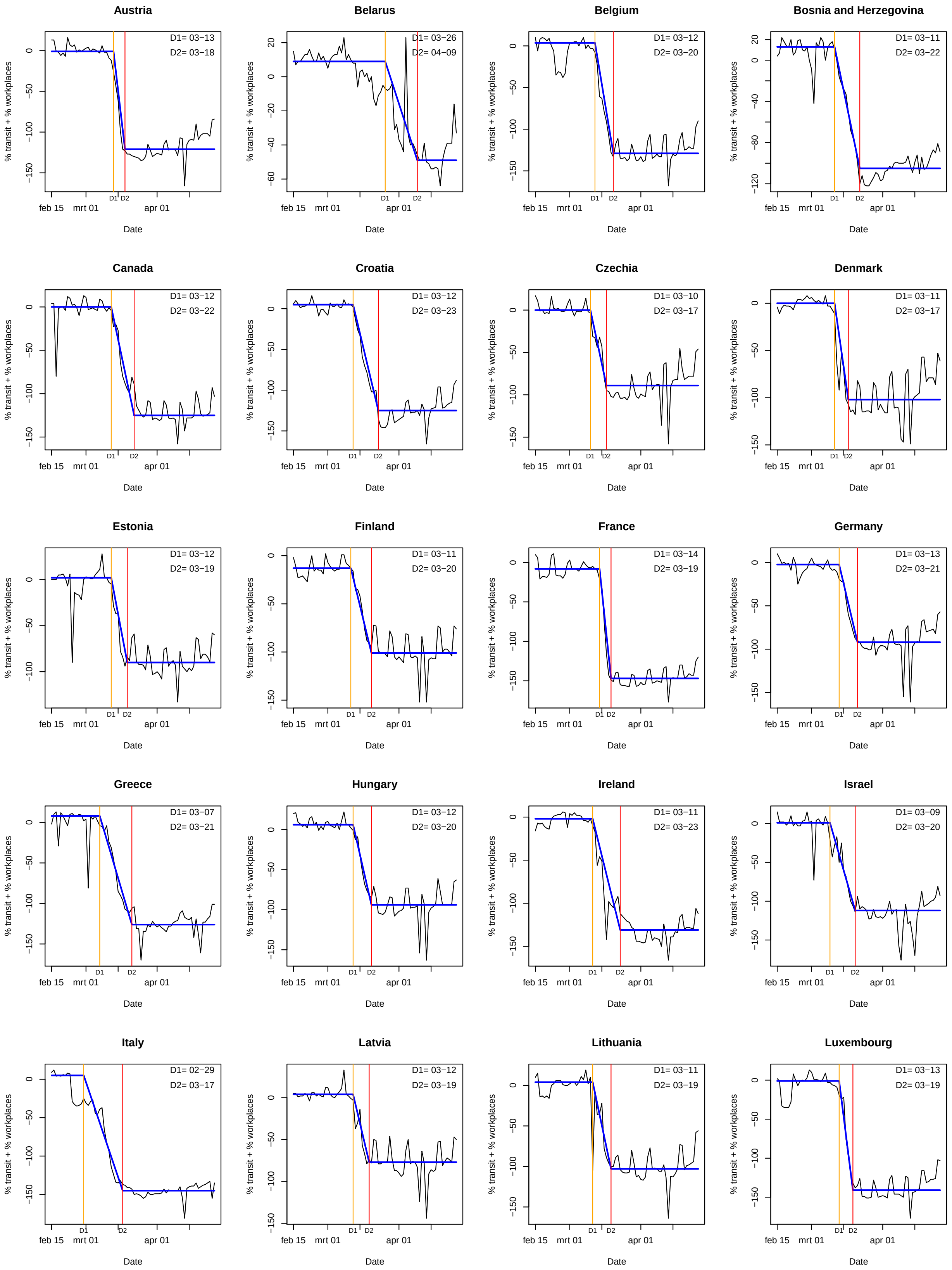
Austria
D1= 03−13
D2= 03−18
Belarus
D1= 03−26
D2= 04−09
Belgium
D1= 03−12
D2= 03−20
Bosnia and Herzegovina
D1= 03−11
D2= 03−22
Canada
D1= 03−12
D2= 03−22
Croatia
D1= 03−12
D2= 03−23
Czechia
D1= 03−10
D2= 03−17
Denmark
D1= 03−11
D2= 03−17
Estonia
D1= 03−12
D2= 03−19
Finland
D1= 03−11
D2= 03−20
France
D1= 03−14
D2= 03−19
Germany
D1= 03−13
D2= 03−21
Greece
D1= 03−07
D2= 03−21
Hungary
D1= 03−12
D2= 03−20
Ireland
D1= 03−11
D2= 03−23
Israel
D1= 03−09
D2= 03−20
Italy
D1= 02−29
D2= 03−17
Latvia
D1= 03−12
D2= 03−19
Lithuania
D1= 03−11
D2= 03−19
Luxembourg
D1= 03−13
D2= 03−19
% transit + % workplaces
Date
feb 15
mrt 01
apr 01
D1
D2

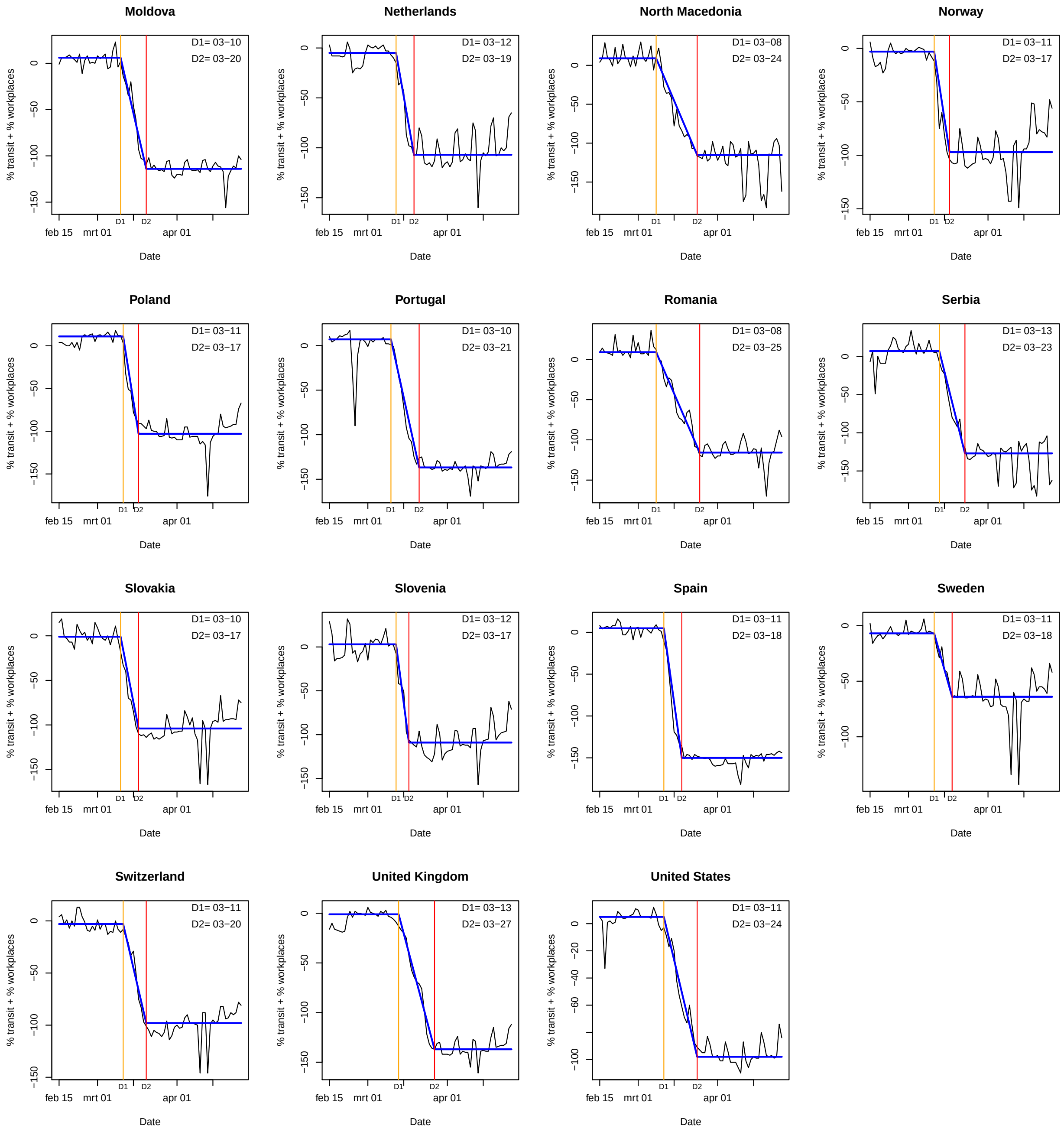
Moldova
D1= 03−10
D2= 03−20
Netherlands
D1= 03−12
D2= 03−19
North Macedonia
D1= 03−08
D2= 03−24
Norway
D1= 03−11
D2= 03−17
Poland
D1= 03−11
D2= 03−17
Portugal
D1= 03−10
D2= 03−21
Romania
D1= 03−08
D2= 03−25
Serbia
D1= 03−13
D2= 03−23
Slovakia
D1= 03−10
D2= 03−17
Slovenia
D1= 03−12
D2= 03−17
Spain
D1= 03−11
D2= 03−18
Sweden
D1= 03−11
D2= 03−18
Switzerland
D1= 03−11
D2= 03−20
United Kingdom
D1= 03−13
D2= 03−27
United States
D1= 03−11
D2= 03−24
% transit + % workplaces
Date
feb 15
mrt 01
apr 01
D1
D2
