# Supplementary material for: Behavioral changes before lockdown and decreased retail and recreation mobility during lockdown contributed most to controlling COVID-19 in Western countries
Source: BMC Public Health. 2021 Apr 6;21:654. doi: 10.1186/s12889-021-10676-1 (PMC8022318; doi:10.1186/s12889-021-10676-1)
Supplement: Supplementary file 2 — Additional file 2. Data and analysis scripts [file 12889_2021_10676_MOESM2_ESM.pdf]

Supplementary material 2: Data and analysis scripts

Here we describe the organization of the github repository which contains the data files and scripts that can be used to reproduce the results.

1 Source code and data

The source code and analysis files are hosted at: `https://github.com/kdeforche/epi-western-analysis`. Here we describe the main analysis scripts, data files, and steps to do the analysis.

1.1 R analysis scripts

R/data/ecdc/data.R script for estimating d_1 and d_2 from Google Mobility data, and reading and preparing ECDC data for one country for analysis.

R/models/model-tv2-cmp.R model used for main analysis.

R/models/model-tv3p-cmp.R model with informative priors and an additional transition point in R_t.

R/models/model-tv2p-cmp.R model with informative priors used as baseline for model testing for *model-tv3p*.

R/MCMC/fitMCMC-drj.R R script to estimate the model parameters using MCMC for one data set.

R/MCMC/analyzeMCMC.R R script to analyze an estimated model for one data set.

analyses/ecdc/analysis.R R script to calculate cross-country statistics

1.2 Data files

R/data/ecdc/ecdc.csv Data of incidence and deaths retrieved from ECDC `https://opendata.ecdc.europa.eu/covid19/casedistribution/csv` on 6 June 2020.

R/data/ecdc/Global_Mobility_Report.csv Google Mobility Report retrieve fromd Google `https://www.google.com/covid19/mobility/` on 4 May 2020.

2 Analysis steps

2.1 Bayesian model parameter estimation

To obtain a sample from the posterior distribution of parameters fitted to data for a country with 2-leter country code *CC*, using the model-tv2-cmp.R:

```
cd analyses/ecdc/tv2-cmp
Rscript ../../../R/MCMC/fitMCMC-drj.R --args CC
```

The samples are subsequently analyzed to infer marginalized estimates, using :

```
Rscript ../../../R/MCMC/analyzeMCMC.R --args CC
```

This results in a file `CC_analysis.csv`, a plot of key parameters for the samples `CC_sample.pdf` that were used (without burn-in), and a figure with the 6 epidemiological graphs (of Suppl 4), `CC_graphs.pdf`.

The same steps are used to estimate the other two models.

2.2 Statistical analyses

First combine all of the analysis CSV files (each containing 1 data line), into a single CSV file, using:

```
head -n 1 BE_analysis.csv > analysis.csv
for i in HU CA IL GR AT BA BE BY CH CZ DE EE DK ES FI FR GB HR IT LT LU IE LV MD MK NL NO PL
PT RO RS SE SI SK US; do tail -q -n1 $i_analysis.csv >> analysis.csv; done
```

Next run the commands from `analysis.R` in the folder with the resulting `analysis.csv` CSV file.
