# Supplementary material for: Behavioral changes before lockdown and decreased retail and recreation mobility during lockdown contributed most to controlling COVID-19 in Western countries
Source: BMC Public Health. 2021 Apr 6;21:654. doi: 10.1186/s12889-021-10676-1 (PMC8022318; doi:10.1186/s12889-021-10676-1)
Supplement: Supplementary file 3 — Additional file 3. Supplementary methods and results. [file 12889_2021_10676_MOESM3_ESM.pdf]

Supplementary material 3: Supplementary methods and results

1 Supplementary methods

1.1 Relation between infections, cases and deaths

The data collected on cases and deaths are related to infections through biological and clinical processes, but also impacted by other unknown delays, such as testing and reporting practices. The time from infection to confirmed test depends largely on whether the diagnosis was performed based on presence of mild symptoms (which were in many countries not officially tested during the initial phase of the outbreak because of shortages of test kits), or whether the test was performed at the time of hospitalization, or whether the tests was done early on within the context of contact tracing. Time between infection and reported death may be dependent on treatment availability, clinical choices and practices, but also on reporting delays due to disparate policies in different countries.

Here we assumed for both periods a gamma distribution, and co-estimated the shape k and scale θ parameters. Incidence of diagnosed cases $C(t)$ and deaths $D(t)$ were then derived from the state of the SEIR model by convolving a Gamma density function function $\Gamma(k_c, \theta_c)$ and $\Gamma(k_d, \theta_d)$ over the number of new infections (transitioning from S to E compartment), with a rate of ρ_c (test rate) and ρ_d (infection fatality rate).

$$C(t) = -\rho_c \frac{dS(t)}{dt} * \Gamma(k_c, \theta_c)$$
$$D(t) = -\rho_d \frac{dS(t)}{dt} * \Gamma(k_d, \theta_d)$$

The parameters k and θ were co-estimated using a parameterization of their mean μ and standard deviation σ. Because the dates of mobility changes d_1 and d_2 were independently estimated from Google Mobility data, the incidence data was informative for the identification of these distribution parameters, and given the uncertainty as discussed above, a uniform prior was chosen for their mean μ. Initial experiments with an informative prior $\sigma = 8.5$ used by Flexman *et al.* (*1*) resulted in bad model fits for a number of countries (including Netherlands, Spain, and Switzerland), due to the inability of the model to capture the fast rise and fall of cases and deaths (see for example for Spain in Figure 1). This can be explained by considering that the infection-to-case and infection-to-death distributions may be interpreted as stepped impedence resonator (SIR) low pass filters of new infections over time, thus limiting the rate at which changes in the resulting time curves of incidence of confirmed tests and deaths may occur: a Gamma distribution with σ = 8.5 days was calculated to have a 20 dB stop-band cut-off at 1/20 day-1 using freqz, which intuitively implies that changes cannot occur faster than in 20 days. When using less informative priors, lower values for $\sigma \simeq 5$ were being estimated instead. The estimate of $\mu_d = 8.5$ by Flexman *et al.* (*1*) was based on the combination of estimated infection-to-onset distributions (incubation period) and onset-to-death distributions from clinical date of the Wuhan epidemic, assuming independence. Our analysis thus suggests that incubation time and onset-to-death are not independent, and a longer incubation time is associated with a shorter period for onset-to-death.

1.2 Data fitting of cases and deaths

For the likelihood calculation for each incidence data point (number of cases or number of deaths) a negative binomial distribution was used with μ equal to the expected count, and a suitable dispersion parameter r. Death incidence data was considered more trustworthy than case reporting (which is influenced also by a possibly changing testing strategy). Therefore, a low value $r_c = 3$ was used to reflect a low weight given to case date, while a higher value for the dispersion parameter $r_d = 60$ was used since the data for incidence of deaths is expected to be more reliable, but accounting for observed variance due to clustering effects and outliers (presumably caused by back-reported deaths) seen in several countries.

1.3 Assumed constants and prior distributions

The average generation time G was assumed a constant and calculated as $T_{\text{lat}} + 1/(2T_{\text{inf}})$ (*2*).

Table 1 lists the model parameters and their initial values or prior distributions for three models used in the analyses.

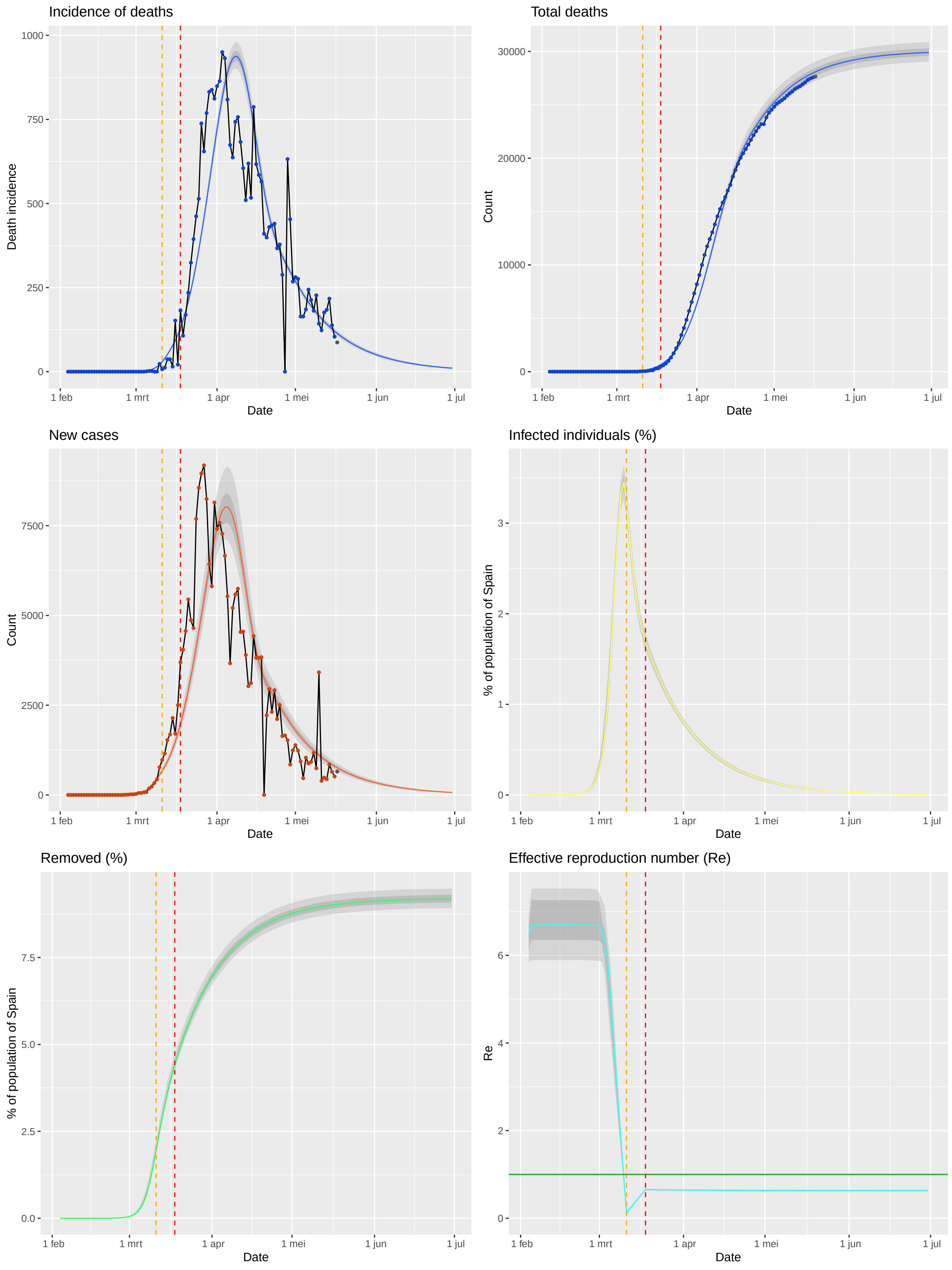

Figure 1: Estimated model for Spain using $\sigma_c = \sigma_d = 8.5$, showing how the bandwidth limitation of the infection-to-test and infection-to-death distributions cannot capture the quick rise and fall of cases and deaths, resulting in a bad fit.

Parameter	Description	Value or prior distribution		
		model-2	*model-2p*	*model-3p*
N	population size	ECDC		
d_1	date of start of mobility changes	estimated from mobility report		
d_2	date of start of lockdown	estimated from mobility report		
G	generation time	5.2 days (*3*)		
T_{lat}	latent period duration	3 days (*4*)		
ρ_c	test rate	$\text{uni}(0, 1)$		
ρ_d	infection fatality rate	0.007 (*5*)		
$S[0]$	initial Susceptible count	$N - 1$		
$E[0]$	initial Exposed count	1		
$I[0]$	initial Infectious count	0		
$R[0]$	initial Removed count	0		
$R_{t,0}$	initial basic reproduction number R_0 until d_0	$\text{uni}(0, 8)$	$\mathcal{N}(3.6, 0.6)$	
$R_{t,1}$	basic reproduction number at d_1	$\text{uni}(0, 8)$	$\mathcal{N}(2.1, 0.4)$	
$R_{t,2}$	basic reproduction number at d_2	$\text{uni}(0, 8)$	$\mathcal{N}(0.8, 0.1)$	
$R_{t,1} - R_{t,0}$	change in R_t from d_0 to d_1	$\mathcal{N}(0, 1)$	$\text{uni}(-\infty, \infty)$	
$R_{t,2} - R_{t,1}$	change in R_t from d_1 to d_2	$\mathcal{N}(0, 1)$	$\text{uni}(-\infty, \infty)$	
μ_c	infection-to-test latency	uni(5, 25)	$\mathcal{N}(14, 5)$	
σ_c	test latency uncertainty	$\mathcal{N}(5, 1)$	$\mathcal{N}(4.8, 0.5)$	
μ_d	infection-to-death latency	uni(10, 50)	$\mathcal{N}(26.5, 5)$	
σ_d	death latency uncertainty	$\mathcal{N}(5, 1)$	$\mathcal{N}(5.2, 0.5)$	
d_0	date of transition from R_0	$d_1 + \mathcal{N}(0, 10)$	$d_1 + \mathcal{N}(-6.5, 4)$	
d_3	date of transition point during lockdown	n.a.		$d_2 + \mathcal{N}(15, 5)$
d_4	end of transition during lockdown	n.a.		$d_3 + \mathcal{N}(0, 2)$
$R_{t,3}$	basic reproduction number at d_4	n.a.		$\text{uni}(0, 8)$
$R_{t,3} - R_{t,2}$	change in R_t from d_3 to d_4	n.a.		$\mathcal{N}(0, 0.3)$

Table 1: Model parameters and their constant assumed values for fixed parameters, or prior distribution for estimated parameters for three models. *model-2*: model with uninformative priors that was used for main results; *model-2p*: same model with informative priors based on country average values, used as baseline for model selection comparison with *model-3p*; *model-3p*: model with an additional transition point for R_t during lockdown (between dates d_3 and d_4).

1.4 Statistical analyses

For multivariate models, collinearity was assessed by calculating variance-inflation factors using vif. Variance-inflation factors higher than 6 were considered problematic, they were removed starting iteratively with the highest value.

The average effect of a mobility change on reduction of $R_{t,2}$ was estimated by applying the estimated effect to the median mobility reduction in the 35 analyzed countries. For example, for retail and recreation on the estimated effect (-0.07 +/- 0.02 per 10% mobility reduction) was applied to the median mobility reduction of -66%. Similarly, the upper bound of the impact of increased mobility to parks was estimated by applying the estimated effect (-0.021 +/- 0.009 per 10% mobility increase) to the 95% upper quantile of the mobility in the 35 analyzed countries (+53%).

To evaluate whether the $R_{t,2}$ value in Sweden could equally be explained by the linear model using changes in mobility, the influence of the data for Sweden was diagnosed using lm.influence (leave-one-out cross-validation) and the residual error of the model prediction was compared to the residual error distribution.

2 Model parameter estimates

The time curves for each country estimated using *model-2* are shown in Suppl 4 and parameter estimates are summarized in Tables 2 and 3.

3 Model with an additional transition point for R_t during lockdown

Since the initial model *model-2* was found to not be applicable to the data of Slovakia, a model *model-3p* with an additional transition point for R_t during lockdown was considered. To allow this transition point to be identified, all data (until June 3th) was used to fit this model. To obtain realistic models even for countries with a low amount of cases (such as Slovakia), informative priors were used based on estimates from *model-2*. Support for the additional transition point was evaluated using Deviance Information Criterion (DIC (*6*)) in comparison with a model *model-2p* that was estimated on the same data with the same priors but without the additional change point. DIC was from the posterior MCMC samples using:

$$\text{DIC} = p_V + \overline{D(\theta)}$$
$$D(\theta) = -2 \log p(y|\theta)$$
$$p_V = \frac{1}{2}\overline{\text{var}(D(\theta))}$$

The resulting estimates for the additional change of transmission during lockdown is indicated in Table 4. For 11 of the 35 countries (Bosnia and Herzegovina, Canada, Croatia, Germany, Italy, Netherlands, Romania, Serbia, Slovakia, Slovenia, and Switzerland), a reduction of transmission was found during lockdown (where $\Delta\text{DIC} < -5$), for 3 countries (North Macedonia, Portugal and Spain) a signicant increase of transmission, and for the remaining 21 countries no significant change in transmission during lockdown was detected. The reason for these detected changes may be a real change in transmission, but could also be due to some of the limitations inherent to the model or data (see limitations discussed in the main article). That for a large majority of the countries no significant change in transmission was observed during lockdown does however confirm that lockdown was indeed the final effective measure for controlling transmission in these countries.

4 Robustness to different assumptions of the duration of the latent period

To assess the robustness of the findings to different assumptions of latent period duration T_{lat} (which was assumed to be 3 days), models were re-estimated using an assumption of 2 and 4 days, by keeping generation time the same (and thus by varying T_{inf} as well). Table 5 shows the parameter estimates for each of the three assumptions of duration of the latent period. Since the duration of the infectious period decreased as latent period increased, the main observation is a reduction of R_t values larger than 1, and an increase of R_t values smaller than 1.

Univariate associations between different independent predictors (mobility changes in different categories and test rate) showed the same trend and had the same statistical significance using different assumed periods for T_{lat} (Tables 6 and 7). The improved fit when assuming a lower latent period duration of 2 days would suggest that this value of 2 is more suitable, implying that on average about 3 days of presymptomatic transmission occurs.

5 Robustness to different assumptions of the duration of generation time

To assess the robustness of the findings to different assumptions of generation time G (which was assumed to be 5.2 days), models were re-estimated using an assumption of 4.5 and 5.9 days, by keeping latent period duration the same (and thus by changing T_{inf}). Table 8 shows the parameter estimates for each of the three assumptions of duration of the latent period. Since the duration of the infectious period increased as generation time increased, the main observation is an increase of R_t values larger than 1, and an decrease of R_t values smaller than 1.

Univariate associations between different independent predictors (mobility changes in different categories and test rate) showed the same trend and had the same statistical significance using different assumed periods for T_{lat} (Tables 9 and 10).

References

1. Flaxman S, Mishra S, Gandy A, Unwin HJT, Mellan TA, Coupland H, et al. Estimating the effects of non-pharmaceutical interventions on COVID-19 in Europe. Nature. 2020 Jun. Available from: `https://doi.org/10.1038/s41586-020-2405-7`.

2. Åke Svensson. A note on generation times in epidemic models. Mathematical Biosciences. 2007;208(1):300 – 311. Available from: http://www.sciencedirect.com/science/article/pii/S0025556406002094.

3. Ganyani T, Kremer C, Chen D, Torneri A, Faes C, Wallinga J, et al. Estimating the generation interval for coronavirus disease (COVID-19) based on symptom onset data, March 2020. Eurosurveillance. 2020;25(17). Available from: https://www.eurosurveillance.org/content/10.2807/1560-7917.ES.2020.25.17.2000257.

4. He X, Lau EHY, Wu P, Deng X, Wang J, Hao X, et al. Temporal dynamics in viral shedding and transmissibility of COVID-19. Nature Medicine. 2020 May;26(5):672–675. Available from: https://doi.org/10.1038/s41591-020-0869-5.

5. Verity R, Okell LC, Dorigatti I, Winskill P, Whittaker C, Imai N, et al. Estimates of the severity of coronavirus disease 2019: a model-based analysis. The Lancet Infectious Diseases. 2020 2020/04/08. Available from: https://doi.org/10.1016/S1473-3099(20)30243-7.

6. Spiegelhalter DJ, Best NG, Carlin BP, Van Der Linde A. Bayesian measures of model complexity and fit. Journal of the royal statistical society: Series b (statistical methodology). 2002;64(4):583–639.

Country	$R_{t,0}$	$R_{t,1}$	$R_{t,2}$
Austria	4.1 (3.5 – 5.0)	2.4 (1.0 – 3.6)	0.62 (0.58 – 0.66)
Belarus	2.6 (2.4 – 2.9)	1.4 (0.9 – 2.1)	1.03 (0.97 – 1.10)
Belgium	5.0 (4.2 – 6.2)	2.6 (1.6 – 3.5)	0.72 (0.69 – 0.75)
Bosnia and Herzegovina	2.8 (2.0 – 4.0)	1.2 (0.4 – 2.5)	1.00 (0.93 – 1.08)
Canada	3.3 (3.0 – 4.1)	2.3 (1.6 – 3.2)	0.92 (0.87 – 0.98)
Croatia	2.9 (2.3 – 3.7)	1.7 (0.9 – 2.9)	0.59 (0.52 – 0.67)
Czechia	3.9 (3.0 – 5.0)	2.8 (1.0 – 3.9)	0.75 (0.69 – 0.80)
Denmark	4.0 (3.1 – 5.2)	1.8 (0.8 – 2.7)	0.81 (0.76 – 0.86)
Estonia	2.9 (2.2 – 4.1)	1.3 (0.5 – 2.1)	0.63 (0.56 – 0.70)
Finland	3.5 (2.8 – 4.6)	1.8 (1.1 – 2.4)	0.84 (0.77 – 0.91)
France	4.5 (3.9 – 5.3)	2.2 (1.4 – 3.0)	0.69 (0.66 – 0.71)
Germany	5.0 (4.3 – 6.0)	1.9 (1.1 – 2.8)	0.73 (0.70 – 0.76)
Greece	2.9 (2.2 – 4.2)	1.7 (0.7 – 2.5)	0.74 (0.68 – 0.79)
Hungary	2.6 (1.8 – 3.5)	2.1 (1.2 – 3.1)	0.83 (0.76 – 0.90)
Ireland	3.3 (2.3 – 4.5)	2.4 (1.6 – 3.4)	0.61 (0.56 – 0.66)
Israel	3.8 (3.2 – 5.0)	2.1 (1.0 – 3.6)	0.55 (0.48 – 0.63)
Italy	5.7 (5.0 – 6.7)	2.7 (1.6 – 3.4)	0.84 (0.82 – 0.86)
Latvia	2.4 (1.7 – 3.5)	1.4 (0.2 – 2.7)	0.83 (0.76 – 0.90)
Lithuania	3.6 (2.7 – 4.9)	1.7 (0.7 – 2.8)	0.71 (0.64 – 0.78)
Luxembourg	4.2 (3.2 – 5.5)	1.4 (0.5 – 2.5)	0.61 (0.56 – 0.67)
Moldova	2.8 (1.7 – 4.0)	2.4 (1.4 – 3.4)	1.08 (1.01 – 1.16)
Netherlands	4.4 (3.8 – 5.4)	1.7 (1.1 – 2.6)	0.78 (0.75 – 0.81)
North Macedonia	3.1 (2.2 – 4.4)	1.7 (0.9 – 2.4)	0.88 (0.81 – 0.95)
Norway	3.1 (2.4 – 4.1)	1.7 (0.8 – 2.7)	0.67 (0.61 – 0.73)
Poland	3.2 (2.5 – 4.4)	2.5 (1.2 – 3.8)	1.00 (0.94 – 1.05)
Portugal	4.3 (3.6 – 5.5)	2.0 (1.2 – 3.9)	0.80 (0.76 – 0.84)
Romania	3.9 (3.1 – 5.0)	1.7 (1.2 – 3.6)	0.93 (0.89 – 0.98)
Serbia	2.8 (2.0 – 3.9)	2.2 (1.3 – 3.2)	0.76 (0.69 – 0.83)
Slovakia	1.8 (1.0 – 2.7)	1.3 (0.5 – 2.1)	0.41 (0.31 – 0.53)
Slovenia	2.4 (1.8 – 3.4)	1.3 (0.5 – 2.3)	0.55 (0.47 – 0.63)
Spain	6.0 (5.2 – 7.2)	2.3 (1.4 – 3.3)	0.76 (0.74 – 0.78)
Sweden	4.0 (3.4 – 4.9)	1.8 (1.2 – 2.5)	1.00 (0.96 – 1.04)
Switzerland	4.2 (3.5 – 5.2)	1.6 (0.9 – 2.6)	0.62 (0.57 – 0.66)
United Kingdom	4.5 (3.9 – 5.2)	2.4 (1.6 – 3.1)	0.84 (0.82 – 0.86)
United States of America	4.0 (3.5 – 5.3)	2.5 (1.7 – 4.5)	0.91 (0.89 – 0.93)

Table 2: Estimates (95% cri interval) of the time-varying reproduction number R_t using *model-2*: initial basic reproduction numbers ($R_{t,0}$), the reproduction number at start of lockdown ($R_{t,1}$) and during lockdown ($R_{t,2}$).

Country	μ_c	σ_c	μ_d	σ_d
Austria	10.4 (5.3 – 15.6)	4.0 (2.8 – 4.9)	23.8 (21.5 – 27.0)	6.1 (5.0 – 7.2)
Belarus	18.7 (6.8 – 24.1)	4.6 (2.8 – 6.3)	14.4 (10.2 – 21.0)	5.1 (3.2 – 6.9)
Belgium	15.9 (6.1 – 24.0)	7.1 (5.9 – 8.0)	28.1 (26.3 – 30.6)	6.0 (4.9 – 6.6)
Bosnia and Herzegovina	11.8 (5.4 – 21.9)	4.6 (2.8 – 6.3)	19.5 (10.8 – 26.5)	4.8 (2.8 – 6.8)
Canada	23.4 (16.1 – 24.9)	4.7 (3.0 – 6.4)	36.6 (31.3 – 39.4)	7.4 (6.1 – 8.6)
Croatia	16.2 (5.9 – 23.2)	4.4 (2.8 – 6.0)	32.2 (26.1 – 39.1)	6.5 (4.5 – 8.2)
Czechia	14.1 (5.7 – 21.0)	4.9 (3.4 – 6.1)	26.4 (23.1 – 31.5)	4.6 (3.2 – 6.0)
Denmark	13.5 (6.3 – 20.9)	5.6 (4.1 – 7.0)	24.2 (21.4 – 28.1)	4.8 (3.3 – 6.3)
Estonia	11.3 (5.4 – 19.8)	4.7 (3.2 – 6.1)	26.6 (21.1 – 32.7)	5.1 (3.2 – 7.1)
Finland	13.9 (5.8 – 22.7)	5.1 (3.4 – 6.7)	32.0 (28.4 – 37.6)	6.1 (4.0 – 7.7)
France	13.2 (5.7 – 19.5)	5.5 (4.3 – 6.4)	24.6 (22.9 – 27.0)	5.8 (4.9 – 6.4)
Germany	11.7 (5.3 – 18.8)	4.8 (3.5 – 5.8)	28.2 (25.9 – 32.1)	6.6 (5.8 – 7.3)
Greece	11.0 (5.3 – 21.8)	4.5 (3.0 – 6.3)	20.5 (11.5 – 28.9)	5.0 (3.1 – 6.9)
Hungary	20.2 (5.9 – 24.8)	5.7 (3.7 – 7.5)	29.6 (22.2 – 34.6)	5.1 (3.5 – 6.9)
Ireland	14.8 (5.8 – 24.9)	5.9 (4.5 – 8.2)	18.7 (10.1 – 34.2)	8.0 (6.3 – 8.9)
Israel	19.0 (7.1 – 24.3)	4.7 (3.0 – 6.1)	28.7 (23.4 – 34.2)	6.0 (4.4 – 7.5)
Italy	10.4 (5.3 – 17.7)	4.6 (3.4 – 5.9)	18.0 (15.8 – 22.8)	4.9 (4.0 – 5.9)
Latvia	10.4 (5.3 – 22.0)	4.1 (2.6 – 5.8)	30.1 (14.5 – 43.7)	5.2 (3.2 – 7.3)
Lithuania	9.3 (5.2 – 15.8)	3.7 (2.5 – 5.0)	16.8 (10.4 – 24.2)	5.9 (4.0 – 7.4)
Luxembourg	7.8 (5.2 – 12.2)	3.0 (2.1 – 4.1)	14.6 (10.2 – 20.2)	5.8 (4.7 – 7.0)
Moldova	19.8 (5.8 – 24.6)	4.9 (3.0 – 6.8)	25.6 (13.6 – 31.9)	5.1 (3.3 – 7.1)
Netherlands	14.5 (5.7 – 22.4)	6.1 (4.8 – 7.2)	24.0 (21.5 – 27.6)	5.3 (4.2 – 6.2)
North Macedonia	11.7 (5.4 – 20.7)	4.6 (3.0 – 6.2)	18.3 (10.6 – 26.1)	5.2 (3.2 – 7.0)
Norway	12.0 (5.5 – 19.5)	4.7 (3.4 – 6.0)	29.9 (26.5 – 34.7)	5.9 (4.1 – 7.7)
Poland	17.7 (5.8 – 24.2)	5.3 (3.5 – 6.8)	26.3 (20.8 – 32.0)	4.9 (3.2 – 6.7)
Portugal	19.7 (11.8 – 24.1)	4.3 (2.8 – 5.7)	25.2 (20.3 – 29.6)	5.3 (3.9 – 6.6)
Romania	21.0 (6.0 – 24.8)	5.2 (3.5 – 6.7)	26.8 (18.6 – 32.0)	4.5 (2.9 – 6.2)
Serbia	21.7 (6.6 – 24.9)	5.3 (3.4 – 7.3)	21.6 (11.7 – 26.8)	5.6 (3.7 – 7.5)
Slovakia	17.5 (5.6 – 24.8)	5.6 (3.5 – 7.8)	31.2 (22.0 – 39.1)	4.9 (3.1 – 6.8)
Slovenia	14.0 (5.7 – 22.9)	5.3 (3.7 – 6.8)	31.8 (26.8 – 38.2)	5.2 (3.3 – 7.0)
Spain	9.5 (5.3 – 15.0)	4.4 (3.5 – 5.3)	19.4 (17.6 – 21.5)	4.7 (4.0 – 5.3)
Sweden	14.1 (6.1 – 22.1)	6.3 (4.8 – 7.7)	30.6 (27.9 – 33.5)	5.4 (4.1 – 6.6)
Switzerland	15.0 (5.7 – 20.9)	4.3 (2.7 – 5.5)	26.5 (23.1 – 31.6)	6.7 (5.5 – 7.8)
United Kingdom	17.5 (5.9 – 24.7)	7.2 (5.8 – 8.4)	22.9 (20.4 – 26.3)	4.7 (3.6 – 5.6)
United States of America	20.3 (6.5 – 24.6)	4.5 (2.9 – 5.9)	26.3 (20.1 – 30.3)	5.3 (3.8 – 6.5)

Table 3: Estimates (95% cri interval) of infection-to-test latency (mean μ_c and σ_c of gamma distributions), and infection-to-death latency (mean μ_d and σ_d of gamma distributions) using *model-2*.

Country	ΔDIC	Reduction @ d_3 (%)	d_3
Austria	10.73	-28.7 (-59.2 – 13.1)	2020-04-13
Belarus	2.90	-7.2 (-37.4 – 20)	2020-04-23
Belgium	-0.96	11.5 (-1.2 – 27.9)	2020-04-02
**Bosnia and Herzegovina**	**-6.52**	**27.1 (10.5 – 39.7)**	**2020-04-13**
**Canada**	**-18.25**	**20.9 (11.7 – 28.3)**	**2020-04-05**
**Croatia**	**-10.66**	**40.7 (23 – 59.4)**	**2020-04-06**
Czechia	-2.22	-17.1 (-42 – 3.6)	2020-04-02
Denmark	-2.01	13.7 (-1.6 – 25.2)	2020-04-02
Estonia	0.40	-4.4 (-37.5 – 18.3)	2020-04-03
Finland	-2.22	16.7 (-3.5 – 32.5)	2020-04-03
France	3.57	1.8 (-12.6 – 26.2)	2020-03-30
**Germany**	**-18.77**	**25.9 (16.3 – 34.2)**	**2020-04-04**
Greece	1.16	3.1 (-19.2 – 19.8)	2020-04-04
Hungary	-2.91	15.1 (-1.2 – 28.6)	2020-04-04
Ireland	12.66	42.1 (11.6 – 54.7)	2020-03-30
Israel	32.49	-28.5 (-97.2 – 12.8)	2020-04-14
**Italy**	**-25.15**	**14.3 (8.9 – 19.2)**	**2020-04-07**
Latvia	0.35	-9.3 (-39.8 – 11.8)	2020-04-02
Lithuania	-1.24	-17.5 (-50.5 – 7.4)	2020-04-05
Luxembourg	1.16	0.8 (-28.1 – 23.1)	2020-04-02
Moldova	1.59	-13.8 (-42.6 – 4.2)	2020-03-31
**Netherlands**	**-55.73**	**28.9 (22.7 – 34.6)**	**2020-04-06**
North Macedonia	*-23.84*	*-65.7 (-103.3 – -36.4)*	*2020-04-14*
Norway	1.13	12.6 (-12.8 – 31.3)	2020-03-28
Poland	-0.43	11.6 (-3.5 – 22.6)	2020-03-31
Portugal	*-5.63*	*-24.8 (-44.5 – -6.7)*	*2020-04-13*
**Romania**	**-17.75**	**24 (13.9 – 32.7)**	**2020-04-13**
**Serbia**	**-8.05**	**28.5 (11.4 – 40.9)**	**2020-04-07**
**Slovakia**	**-26.85**	**49.4 (35.9 – 59)**	**2020-04-03**
**Slovenia**	**-7.68**	**37.7 (19.6 – 51.6)**	**2020-04-02**
Spain	*-33.65*	*-27.6 (-38.1 – -18.7)*	*2020-04-16*
Sweden	0.94	6.6 (-7.2 – 20.4)	2020-03-30
**Switzerland**	**-31.84**	**35.9 (26.6 – 43.6)**	**2020-04-03**
United Kingdom	9.85	-7.8 (-29.7 – 5.5)	2020-04-16
United States of America	0.27	3.7 (-6.1 – 11.1)	2020-04-08

Table 4: Estimated dates d_3 and reduction in transmission (1 - $R_{t,3}/R_{t,2}$, 95% cri interval) for an additional change in transmission during lockdown. Countries for which an additional reduction or increase in transmission was supported by the data (ΔDIC < -5), are indicated respectively using a bold or italic font.

Variable	$T_{\text{lat}} = 2$	$T_{\text{lat}} = 3$	$T_{\text{lat}} = 4$
$R_{t,0}$	3.8 (2.6 – 5.5)	3.6 (2.4 – 5.2)	3.3 (2.2 – 4.8)
$R_{t,1}$	2.4 (1.8 – 3.2)	2.2 (1.7 – 3.3)	2.0 (1.5 – 2.8)
$R_{t,2}$	0.76 (0.54 – 1.01)	0.78 (0.58 – 1.01)	0.81 (0.62 – 1.01)
1 - $R_{t,1}/R_{t,0}$ (%)	36 (13 – 54)	36 (12 – 54)	36 (10 – 58)
1 - $R_{t,2}/R_{t,1}$ (%)	69 (55 – 80)	67 (49 – 78)	60 (41 – 73)
d_1 - d_0 (days)	6 (-4 – 12)	6 (-6 – 12)	6 (-3 – 13)

Table 5: Comparison of estimated parameters across all countries for different assumed durations of latent period T_{lat} (median values, 95% IQR).

Variable	$T_{\text{lat}} = 2$		$T_{\text{lat}} = 3$		$T_{\text{lat}} = 4$	
Retail and recreation	0.04 +/- 0.01	**	0.04 +/- 0.01	**	0.03 +/- 0.01	**
Grocery and pharmacy	0.01 +/- 0.02		0.01 +/- 0.02		0.01 +/- 0.02	
Parks	0.003 +/- 0.007		0.002 +/- 0.006		0.002 +/- 0.005	
Transit stations	0.03 +/- 0.02		0.03 +/- 0.02		0.02 +/- 0.02	
Workplaces	0.06 +/- 0.02	*	0.05 +/- 0.02	*	0.05 +/- 0.02	*
Residential	-0.12 +/- 0.04	**	-0.11 +/- 0.04	**	-0.10 +/- 0.03	*
$R_{t,1}$	0.05 +/- 0.06		0.04 +/- 0.05		0.04 +/- 0.05	

Table 6: Univariate association (estimate +/- standard deviation) of mobility changes during lockdown (per 10% mobility change) compared to baseline with $R_{t,2}$, for different assumptions of T_{lat}. ** $p < 0.01$; * $p < 0.05$; . $p < 0.1$

Variable	$T_{\text{lat}} = 2$		$T_{\text{lat}} = 3$		$T_{\text{lat}} = 4$	
Retail and recreation	0.07 +/- 0.02	**	0.07 +/- 0.02	**	0.06 +/- 0.02	**
Grocery and pharmacy	-0.04 +/- 0.03		-0.02 +/- 0.03		-0.02 +/- 0.02	
Parks	-0.020 +/- 0.009	*	-0.022 +/- 0.009	*	-0.019 +/- 0.008	*
Transit stations	-0.06 +/- 0.04		-0.06 +/- 0.03	.	-0.05 +/- 0.03	.
Residential	-0.25 +/- 0.08	**	-0.22 +/- 0.07	**	-0.19 +/- 0.06	**
$R_{t,1}$	0.11 +/- 0.05	*	0.06 +/- 0.04		0.06 +/- 0.04	
model adjusted R^2	0.53	***	0.47	***	0.48	***

Table 7: Multivariate models (estimate +/- standard deviation) of mobility changes during lockdown (per 10% mobility change) compared to baseline with $R_{t,2}$, for different assumptions of T_{lat}. Mobility data related to workplaces was left out from the multivariate analysis since this variable was highly correlated with mobility data related to residential and transit stations. ** $p < 0.01$; * $p < 0.05$; . $p < 0.1$

Variable	$G = 4.5$	$G = 5.2$	$G = 5.9$
$R_{t,0}$	3.2 (2.2 – 4.5)	3.6 (2.4 – 5.2)	4.0 (2.6 – 5.9)
$R_{t,1}$	2.0 (1.4 – 2.7)	2.2 (1.7 – 3.3)	2.6 (1.8 – 3.4)
$R_{t,2}$	0.82 (0.64 – 1.01)	0.78 (0.58 – 1.01)	0.74 (0.52 – 1.01)
1 - $R_{t,1}/R_{t,0}$ (%)	38 (11 – 56)	36 (12 – 54)	36 (11 – 55)
1 - $R_{t,2}/R_{t,1}$ (%)	58 (42 – 72)	67 (49 – 78)	71 (57 – 80)
d_1 - d_0 (days)	7 (-3 – 14)	6 (-6 – 12)	6 (-7 – 11)

Table 8: Comparison of estimated parameters across all countries for different assumed durations of generation period G by allowing changes in infectious period duration T_{inf} (median values, 95% IQR).

Variable	$G = 4.5$		$G = 5.2$		$G = 5.9$	
Retail and recreation	0.03 +/- 0.01	*	0.04 +/- 0.01	**	0.04 +/- 0.01	**
Grocery and pharmacy	0.01 +/- 0.02		0.01 +/- 0.02		0.01 +/- 0.02	
Parks	0.002 +/- 0.005		0.002 +/- 0.006		0.003 +/- 0.007	
Transit stations	0.02 +/- 0.02		0.03 +/- 0.02		0.03 +/- 0.02	
Workplaces	0.04 +/- 0.02	*	0.05 +/- 0.02	*	0.06 +/- 0.02	*
Residential	-0.09 +/- 0.03	*	-0.11 +/- 0.04	**	-0.12 +/- 0.04	**
$R_{t,1}$	0.05 +/- 0.06		0.04 +/- 0.05		0.05 +/- 0.05	

Table 9: Univariate association (estimate +/- standard deviation) of mobility changes during lockdown (per 10% mobility change) compared to baseline with $R_{t,2}$, for different assumed durations of generation period G by allowing changes in infectious period duration T_{inf}. ** $p < 0.01$; * $p < 0.05$; . $p < 0.1$

Variable	$G = 4.5$		$G = 5.2$		$G = 5.9$	
Retail and recreation	0.06 +/- 0.02	**	0.07 +/- 0.02	**	0.07 +/- 0.03	**
Grocery and pharmacy	-0.02 +/- 0.02		-0.02 +/- 0.03		-0.03 +/- 0.03	
Parks	-0.016 +/- 0.008	.	-0.022 +/- 0.009	*	-0.023 +/- 0.010	*
Transit stations	-0.05 +/- 0.03	.	-0.06 +/- 0.03	.	-0.06 +/- 0.04	
Residential	-0.17 +/- 0.06		-0.22 +/- 0.07	**	-0.24 +/- 0.08	**
$R_{t,1}$	0.06 +/- 0.04		0.06 +/- 0.04		0.08 +/- 0.04	.
model adjusted R^2	0.48	***	0.47	***	0.50	***

Table 10: Multivariate models (estimate +/- standard deviation) of mobility changes during lockdown (per 10% mobility change) compared to baseline with $R_{t,2}$, for different assumed durations of generation period G by allowing changes in infectious period duration T_{inf}. Mobility data related to workplaces was left out from the multivariate analysis since this variable was highly correlated with mobility data related to residential and transit stations. ** $p < 0.01$; * $p < 0.05$; . $p < 0.1$
