# Supplementary material for: Behavioral changes before lockdown and decreased retail and recreation mobility during lockdown contributed most to controlling COVID-19 in Western countries
Source: BMC Public Health. 2021 Apr 6;21:654. doi: 10.1186/s12889-021-10676-1 (PMC8022318; doi:10.1186/s12889-021-10676-1)

Supplementary material 4: model predictions per country

Legend

This document shows the modeling results for the countries included in in the analyses. The model, together with the data from which it was estimated, is shown for each country in six graphs. The start (d_1) and end (d_2) dates of the lockdown transition period is marked on each graph using an orange and red vertical dashed line. These dates were estimated from Google Mobility Reports. Incidence data of confirmed cases and deaths up to 60 days after d_2 was used (ECDC, https://opendata.ecdc.europa.eu/covid19/casedistribution/csv, accessed 6 June 2020). The data used to fit the model is shown as connected blue or orange dots. Data that was not used for fitting the model is shown in gray. The 50% and 90% cri intervals for the estimates are shown in dark gray and light gray areas.

The six charts are:

1. **Deaths per day**: Incidence plot of deaths per day. The data used to fit the model is shown as connected blue dots.
2. **Total deaths**: Cumulative plot of deaths per day. Model fitting was done on incidence data rather than cumulative data.
3. **New cases**: Incidence plot of new cases per day. The data used to fit the model is shown as connected orange dots.
4. **Infected individuals (%)**: Estimated number of currently infected individuals (sum of E, In and Is compartment states), as a proportion of the population.
5. **Removed (%)**: Estimated number of individuals that were removed (recovered or deceased) (R compartment state), as a proportion of the population.
6. **Effective reproduction number (Re)**: Estimated effective reproduction number based on the model's estimate of how many new infections are being generated each day by infectious individuals.

1 Austria

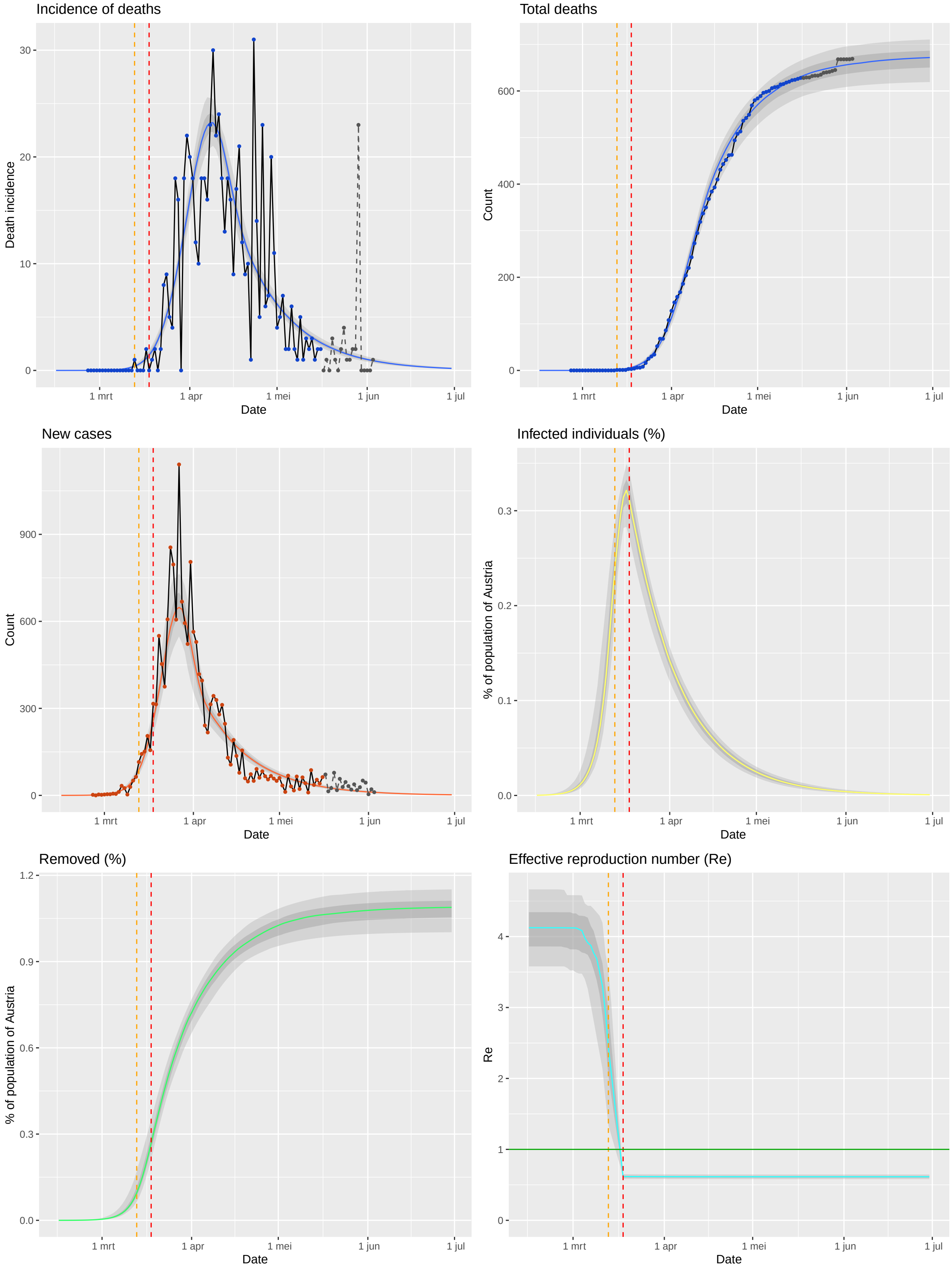
Incidence of deaths
Death incidence
0
10
20
30
1 mrt
1 apr
1 mei
1 jun
1 jul
Date
Total deaths
Count
0
200
400
600
Date
New cases
Count
0
300
600
900
Date
Infected individuals (%)
% of population of Austria
0.0
0.1
0.2
0.3
Date
Removed (%)
% of population of Austria
0.0
0.3
0.6
0.9
1.2
Date
Effective reproduction number (Re)
Re
0
1
2
3
4
Date

2 Belarus

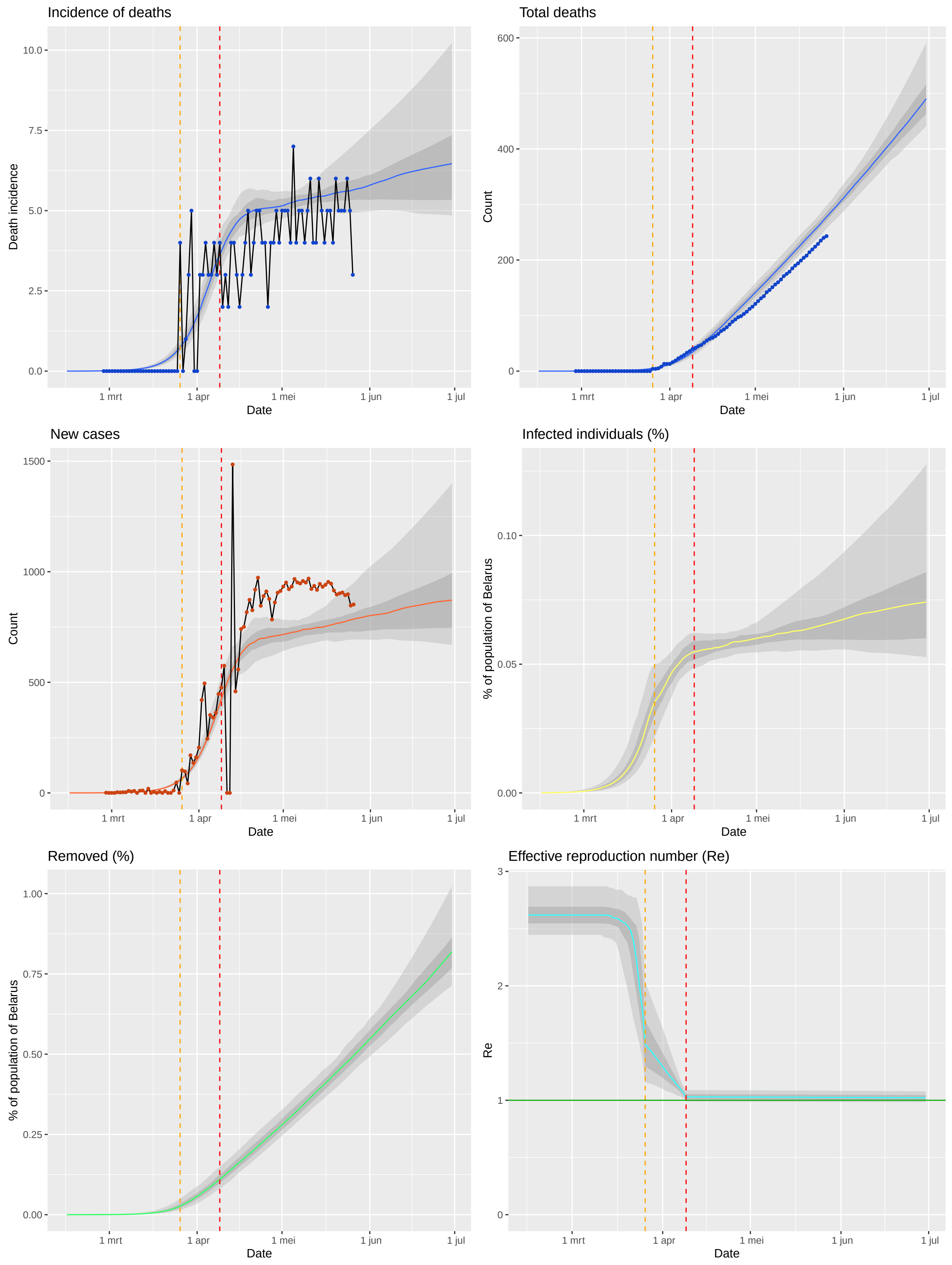

Incidence of deaths
Death incidence
10.0
7.5
5.0
2.5
0.0
1 mrt
1 apr
1 mei
1 jun
1 jul
Date
Total deaths
Count
600
400
200
0
1 mrt
1 apr
1 mei
1 jun
1 jul
Date
New cases
Count
1500
1000
500
0
1 mrt
1 apr
1 mei
1 jun
1 jul
Date
Infected individuals (%)
% of population of Belarus
0.10
0.05
0.00
1 mrt
1 apr
1 mei
1 jun
1 jul
Date
Removed (%)
% of population of Belarus
1.00
0.75
0.50
0.25
0.00
1 mrt
1 apr
1 mei
1 jun
1 jul
Date
Effective reproduction number (Re)
Re
3
2
1
0
1 mrt
1 apr
1 mei
1 jun
1 jul
Date

3 Belgium

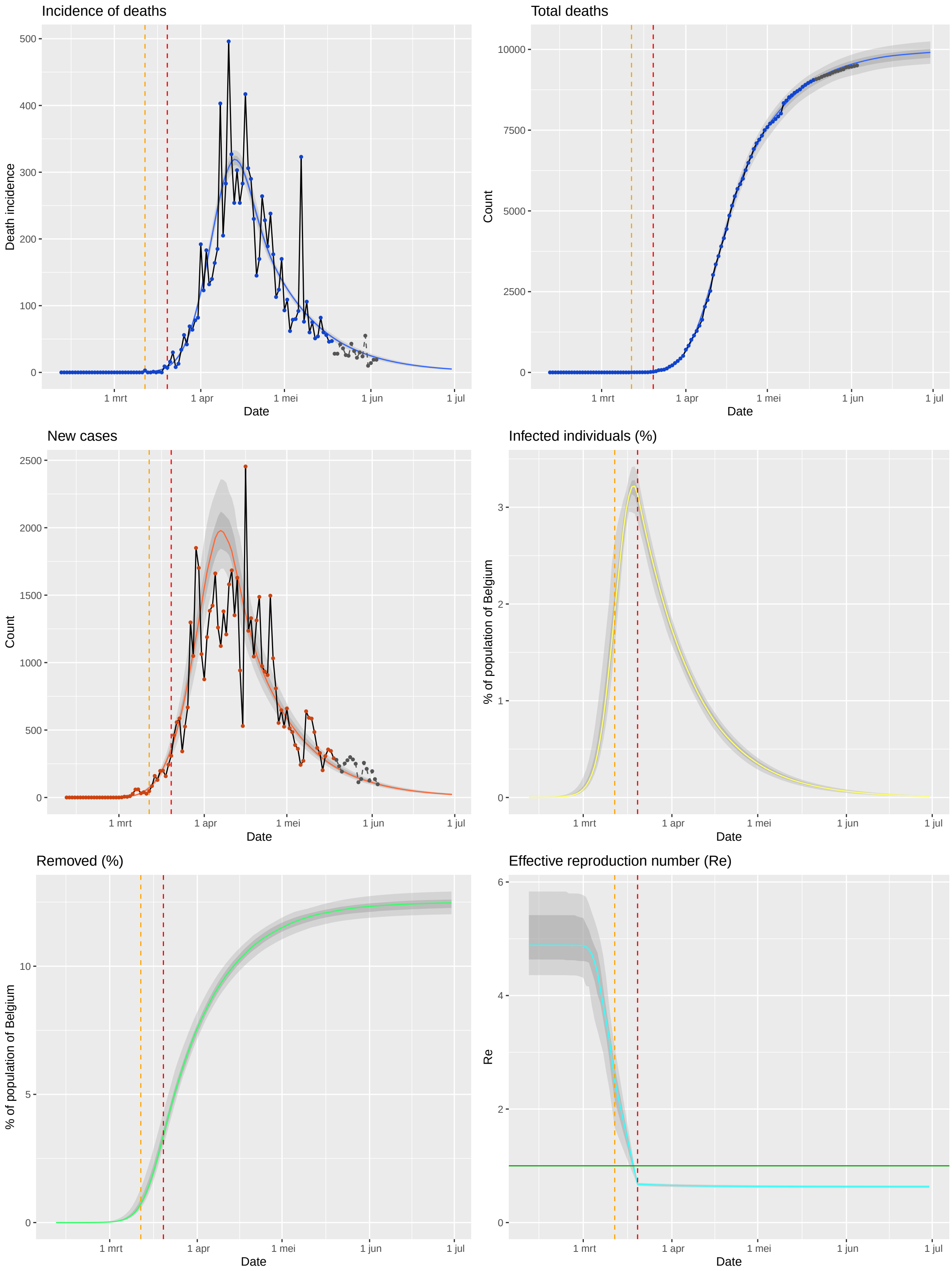

Incidence of deaths
Death incidence
500
400
300
200
100
0
1 mrt
1 apr
1 mei
1 jun
1 jul
Date
Total deaths
Count
10000
7500
5000
2500
0
1 mrt
1 apr
1 mei
1 jun
1 jul
Date
New cases
Count
2500
2000
1500
1000
500
0
1 mrt
1 apr
1 mei
1 jun
1 jul
Date
Infected individuals (%)
% of population of Belgium
3
2
1
0
1 mrt
1 apr
1 mei
1 jun
1 jul
Date
Removed (%)
% of population of Belgium
10
5
0
1 mrt
1 apr
1 mei
1 jun
1 jul
Date
Effective reproduction number (Re)
Re
6
4
2
0
1 mrt
1 apr
1 mei
1 jun
1 jul
Date

4 Bosnia and Herzegovina

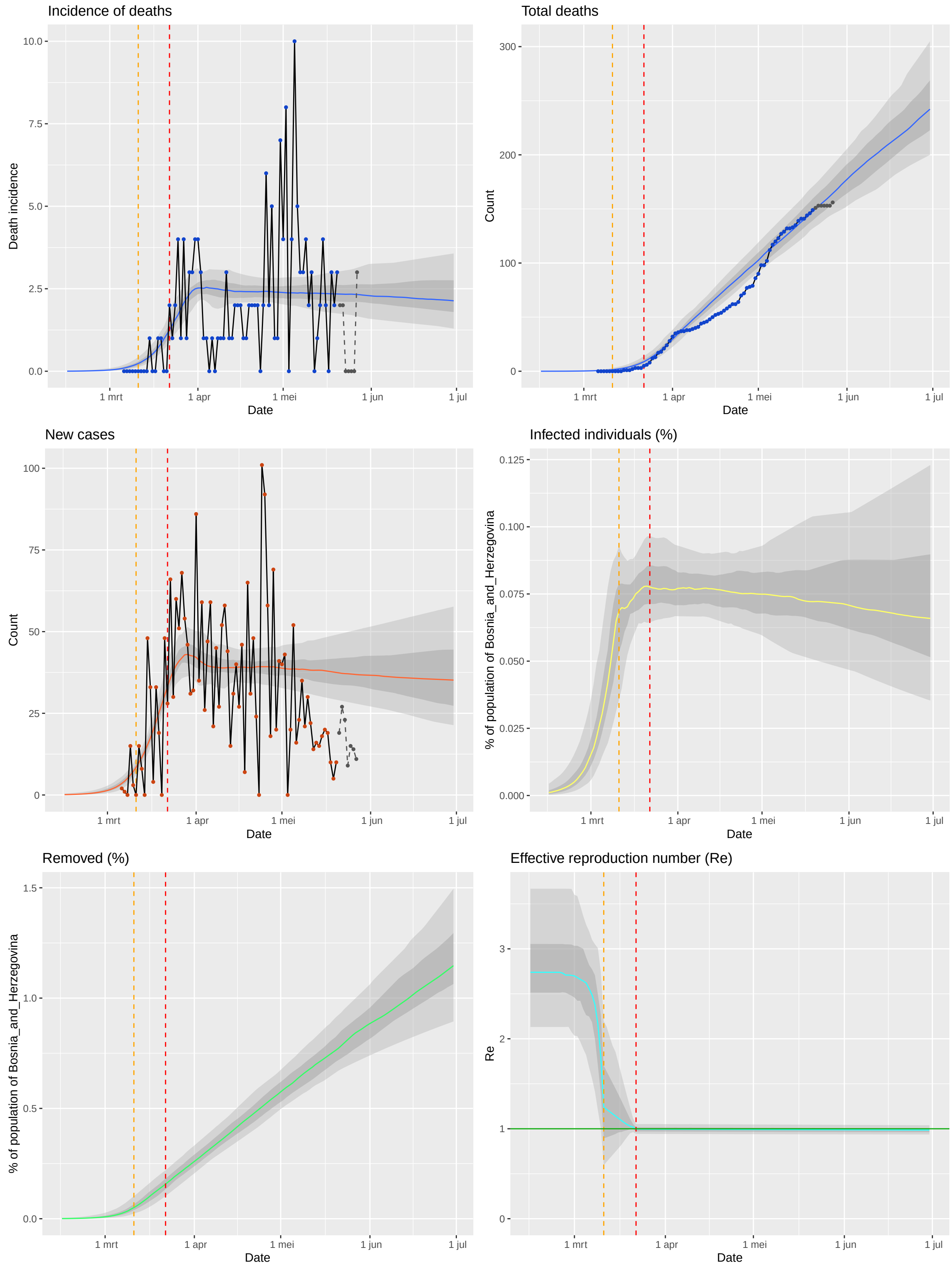

5 Canada

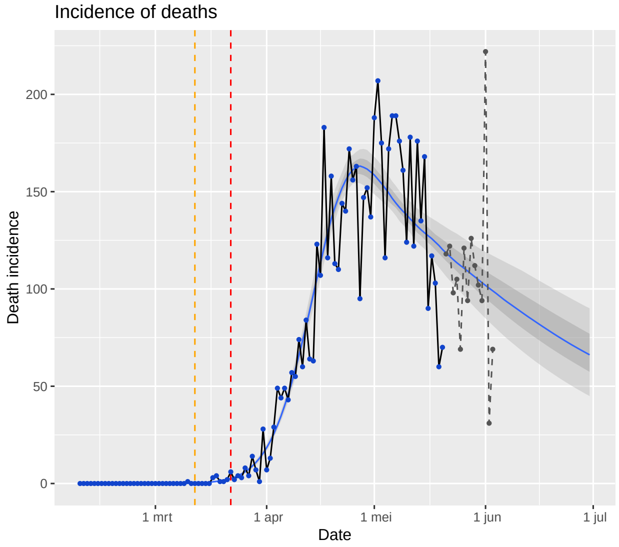
Incidence of deaths
Death incidence
200
150
100
50
0
1 mrt
1 apr
1 mei
1 jun
1 jul
Date

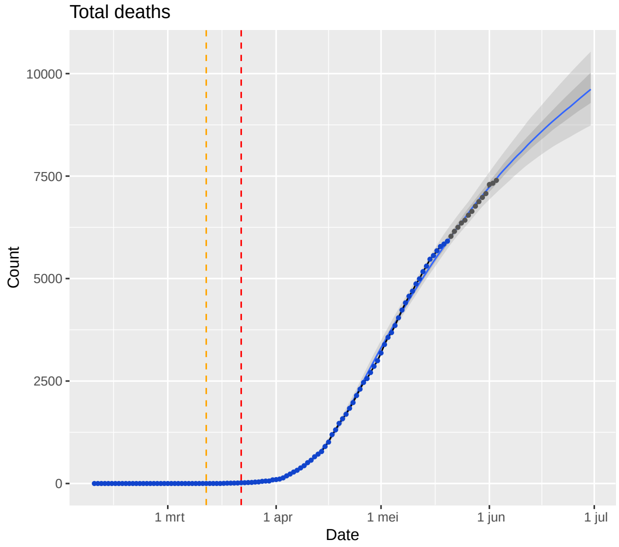
Total deaths
Count
10000
7500
5000
2500
0
1 mrt
1 apr
1 mei
1 jun
1 jul
Date

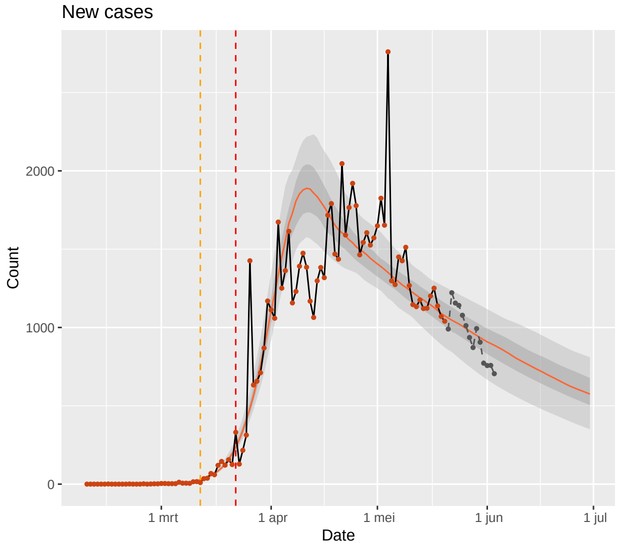
New cases
Count
2000
1000
0
1 mrt
1 apr
1 mei
1 jun
1 jul
Date

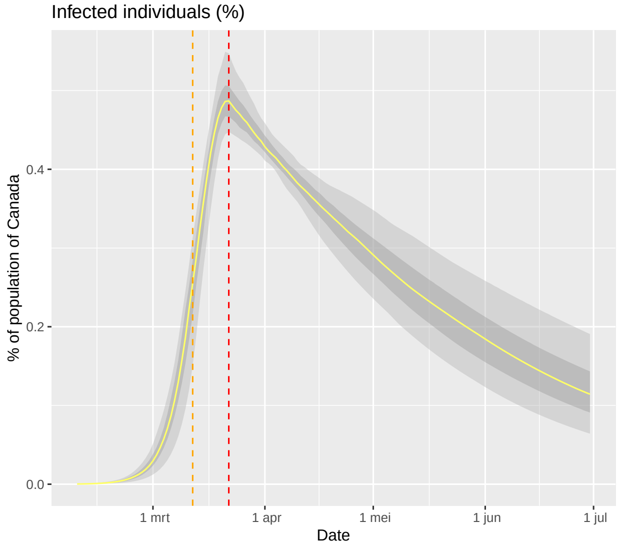
Infected individuals (%)
% of population of Canada
0.4
0.2
0.0
1 mrt
1 apr
1 mei
1 jun
1 jul
Date

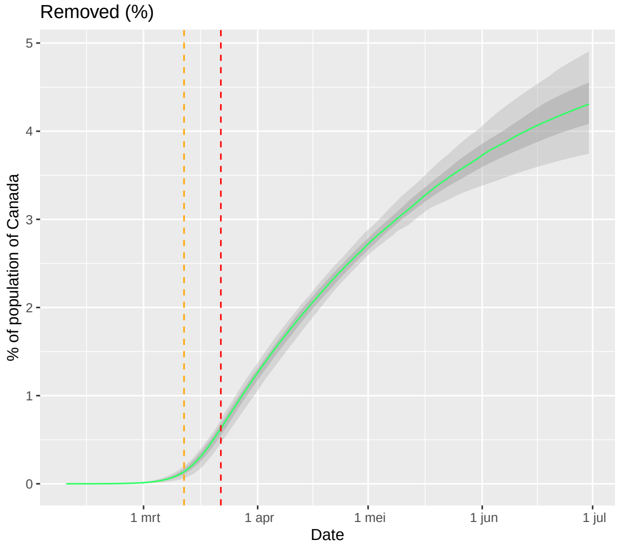
Removed (%)
% of population of Canada
5
4
3
2
1
0
1 mrt
1 apr
1 mei
1 jun
1 jul
Date

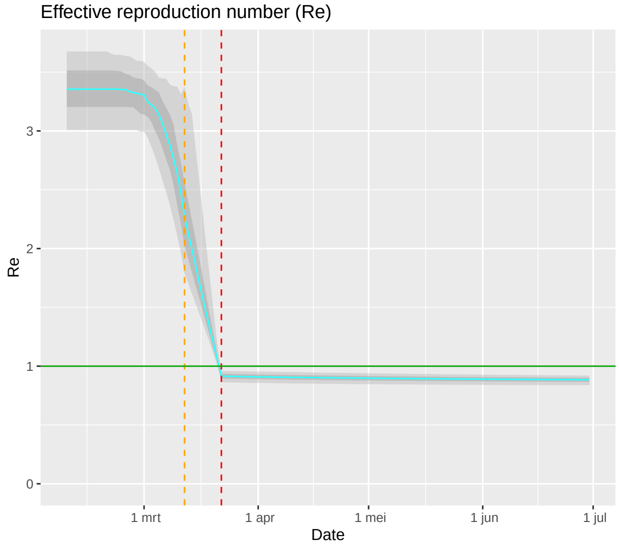
Effective reproduction number (Re)
Re
3
2
1
0
1 mrt
1 apr
1 mei
1 jun
1 jul
Date

6 Croatia

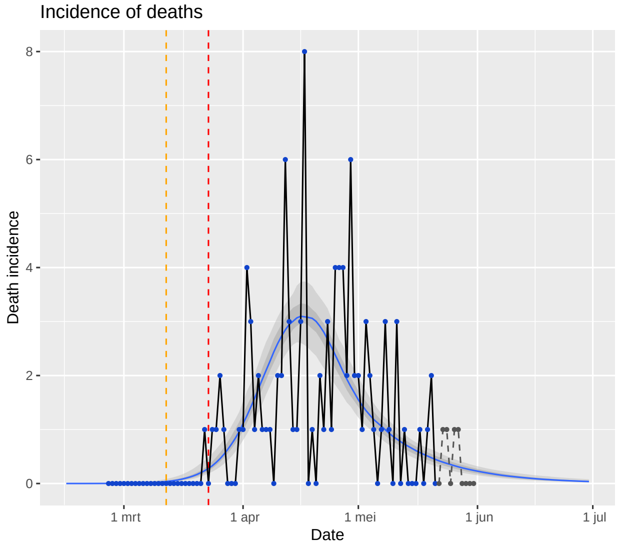
Incidence of deaths
Death incidence
0
2
4
6
8
1 mrt
1 apr
1 mei
1 jun
1 jul
Date

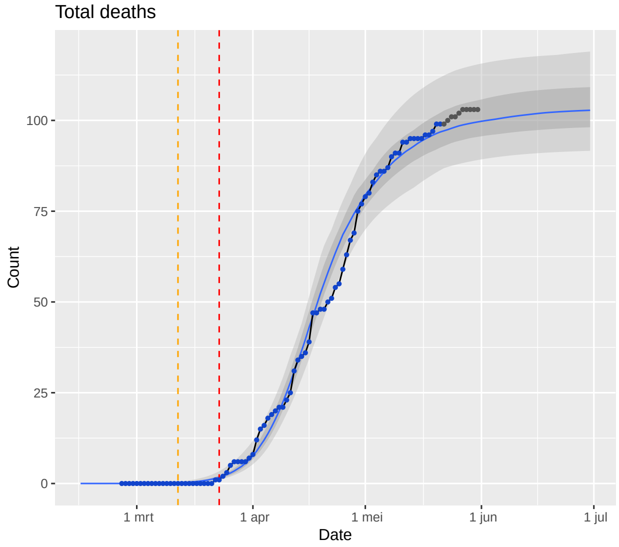
Total deaths
Count
0
25
50
75
100
1 mrt
1 apr
1 mei
1 jun
1 jul
Date

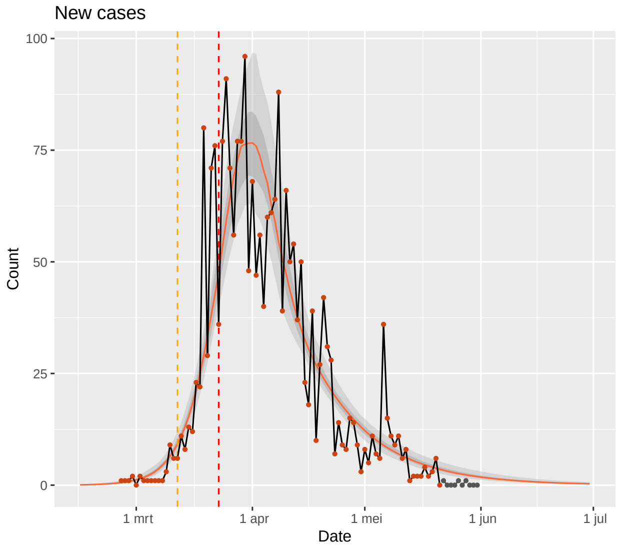
New cases
Count
0
25
50
75
100
1 mrt
1 apr
1 mei
1 jun
1 jul
Date

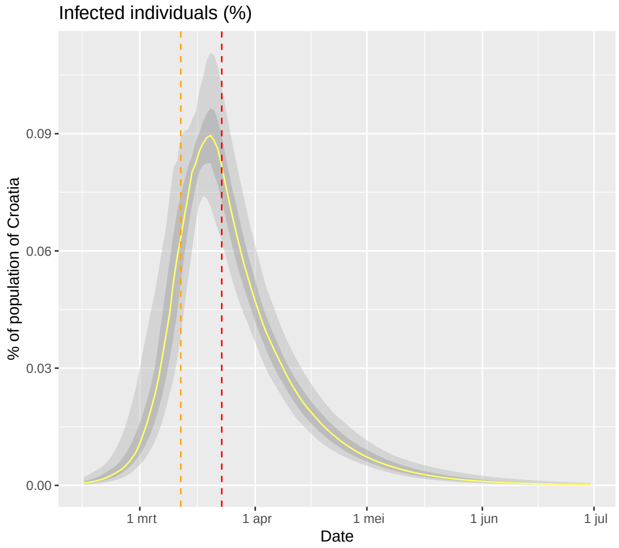
Infected individuals (%)
% of population of Croatia
0.00
0.03
0.06
0.09
1 mrt
1 apr
1 mei
1 jun
1 jul
Date

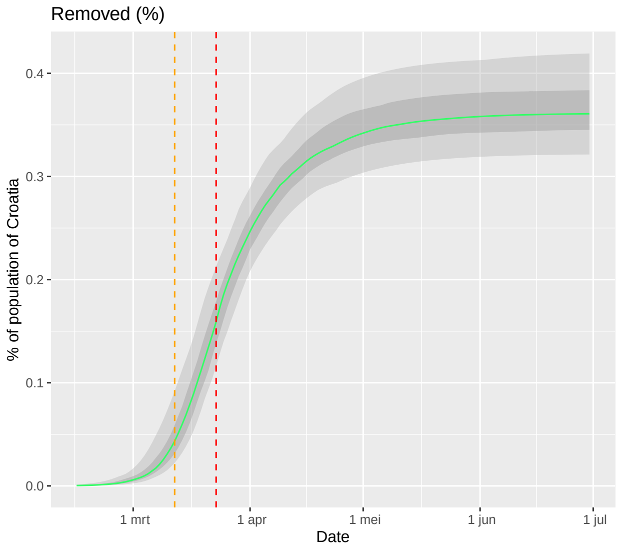
Removed (%)
% of population of Croatia
0.0
0.1
0.2
0.3
0.4
1 mrt
1 apr
1 mei
1 jun
1 jul
Date

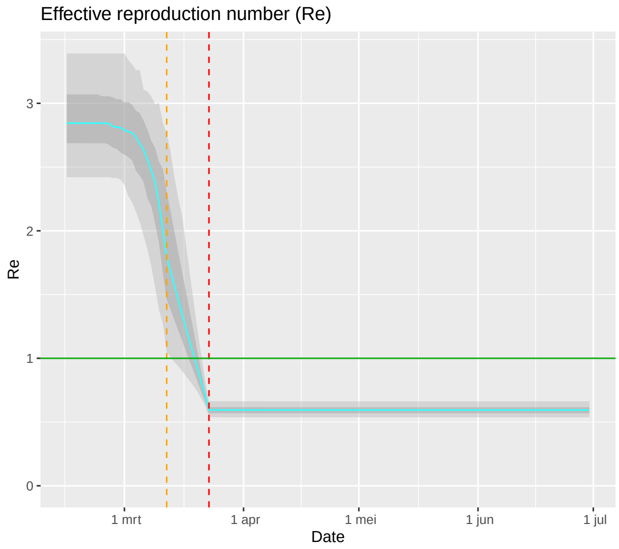
Effective reproduction number (Re)
Re
0
1
2
3
1 mrt
1 apr
1 mei
1 jun
1 jul
Date

7 Czechia

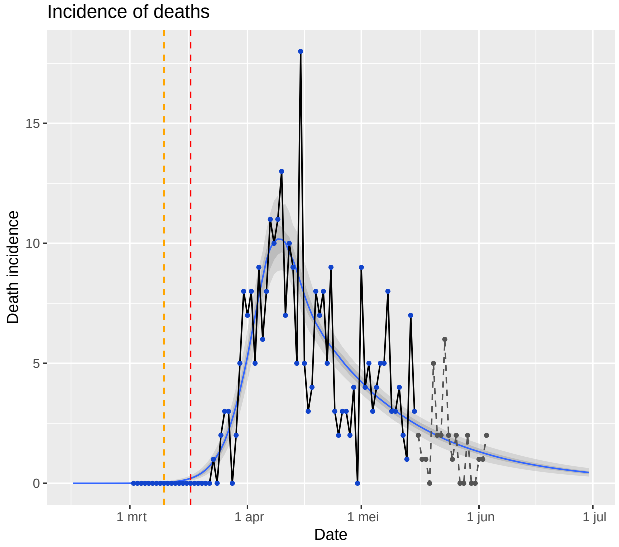

Incidence of deaths
Death incidence
0
5
10
15
1 mrt
1 apr
1 mei
1 jun
1 jul
Date

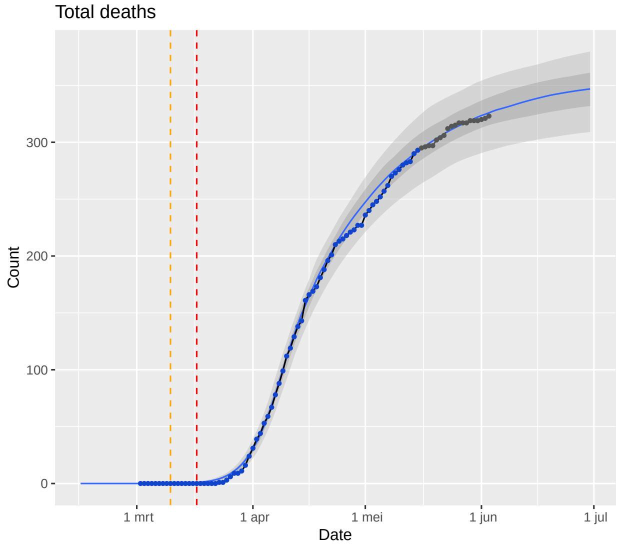

Total deaths
Count
0
100
200
300
1 mrt
1 apr
1 mei
1 jun
1 jul
Date

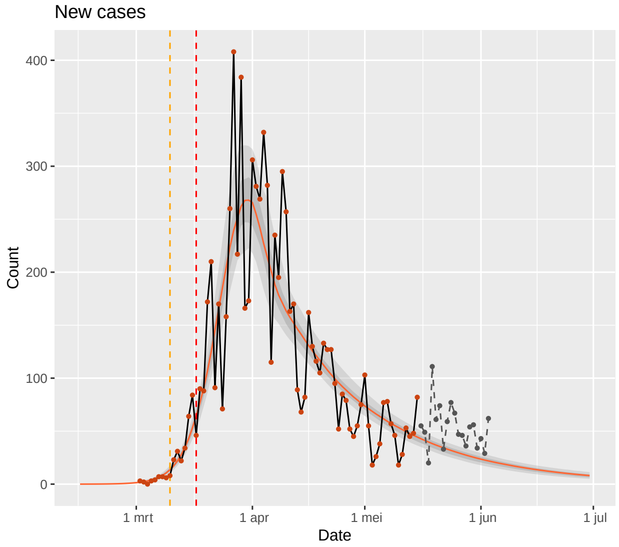

New cases
Count
0
100
200
300
400
1 mrt
1 apr
1 mei
1 jun
1 jul
Date

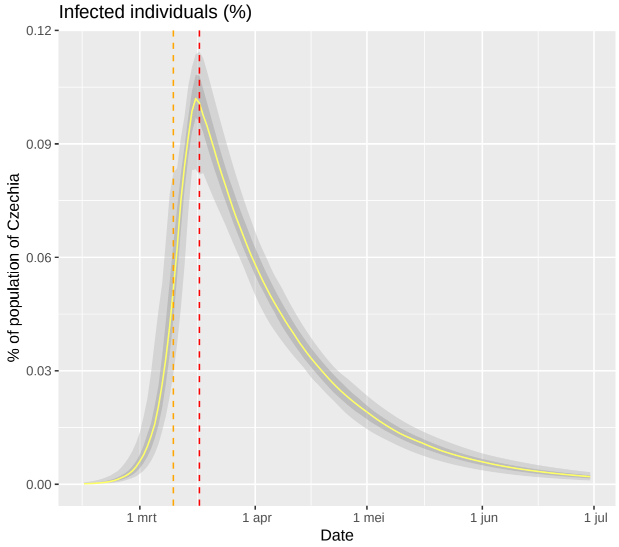

Infected individuals (%)
% of population of Czechia
0.00
0.03
0.06
0.09
0.12
1 mrt
1 apr
1 mei
1 jun
1 jul
Date

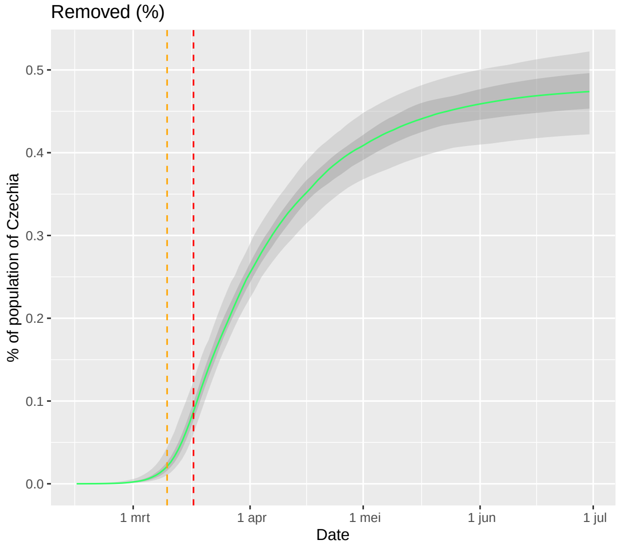

Removed (%)
% of population of Czechia
0.0
0.1
0.2
0.3
0.4
0.5
1 mrt
1 apr
1 mei
1 jun
1 jul
Date

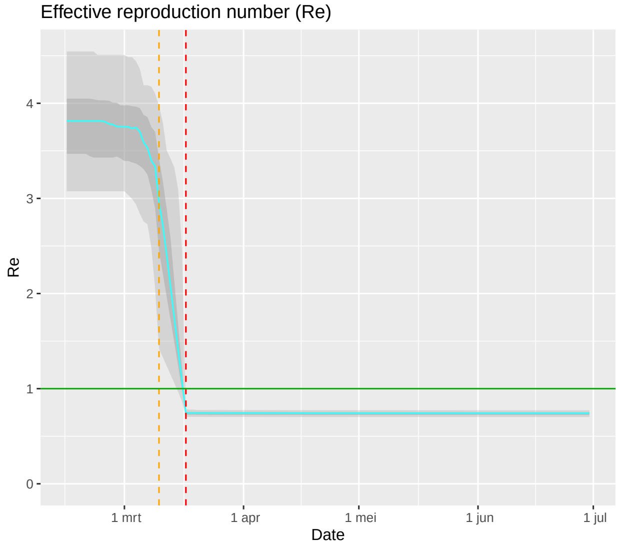

Effective reproduction number (Re)
Re
0
1
2
3
4
1 mrt
1 apr
1 mei
1 jun
1 jul
Date

8 Denmark

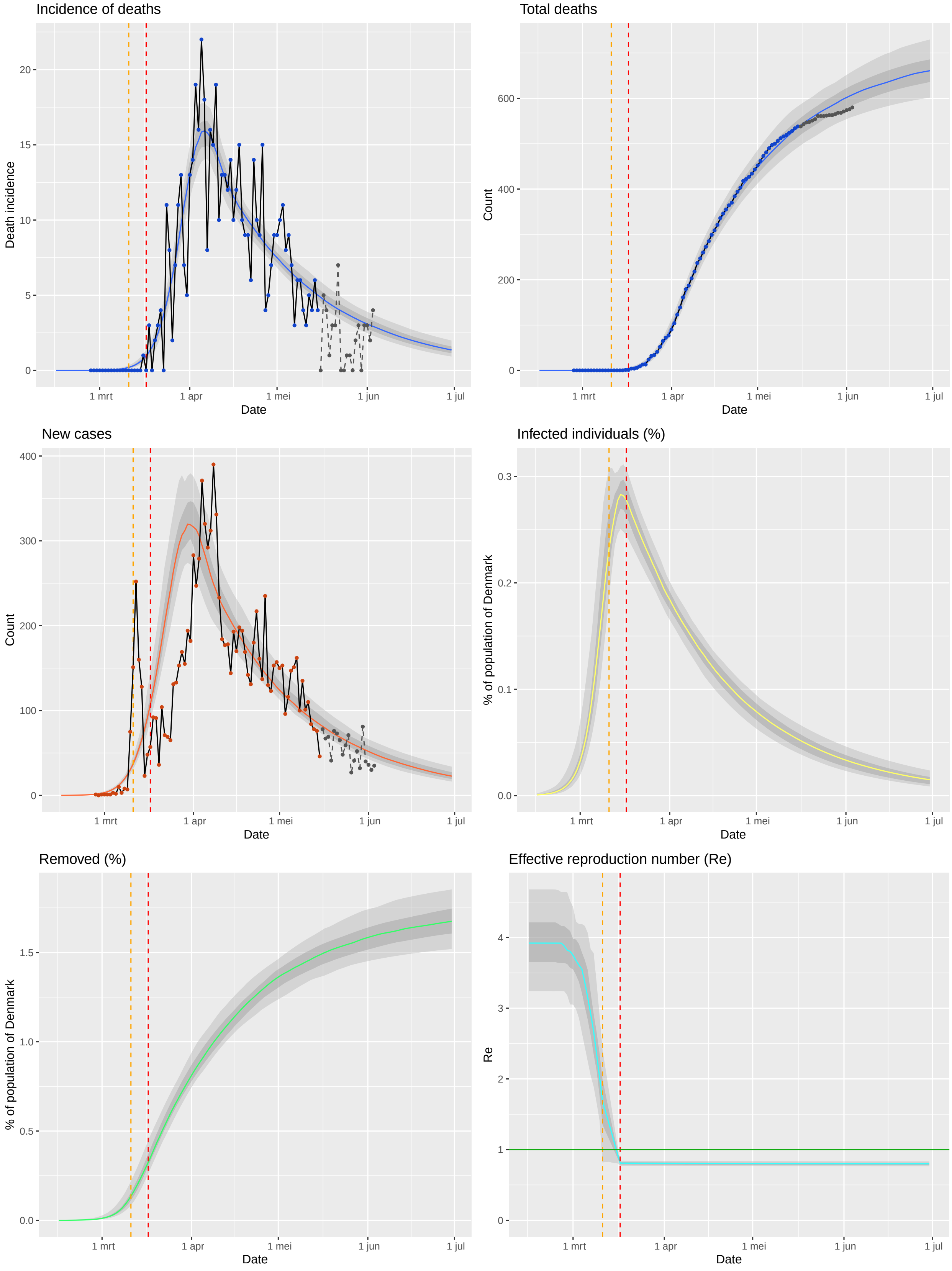
Incidence of deaths
Death incidence
20
15
10
5
0
1 mrt
1 apr
1 mei
1 jun
1 jul
Date
Total deaths
Count
600
400
200
0
1 mrt
1 apr
1 mei
1 jun
1 jul
Date
New cases
Count
400
300
200
100
0
1 mrt
1 apr
1 mei
1 jun
1 jul
Date
Infected individuals (%)
% of population of Denmark
0.3
0.2
0.1
0.0
1 mrt
1 apr
1 mei
1 jun
1 jul
Date
Removed (%)
% of population of Denmark
1.5
1.0
0.5
0.0
1 mrt
1 apr
1 mei
1 jun
1 jul
Date
Effective reproduction number (Re)
Re
4
3
2
1
0
1 mrt
1 apr
1 mei
1 jun
1 jul
Date

9 Estonia

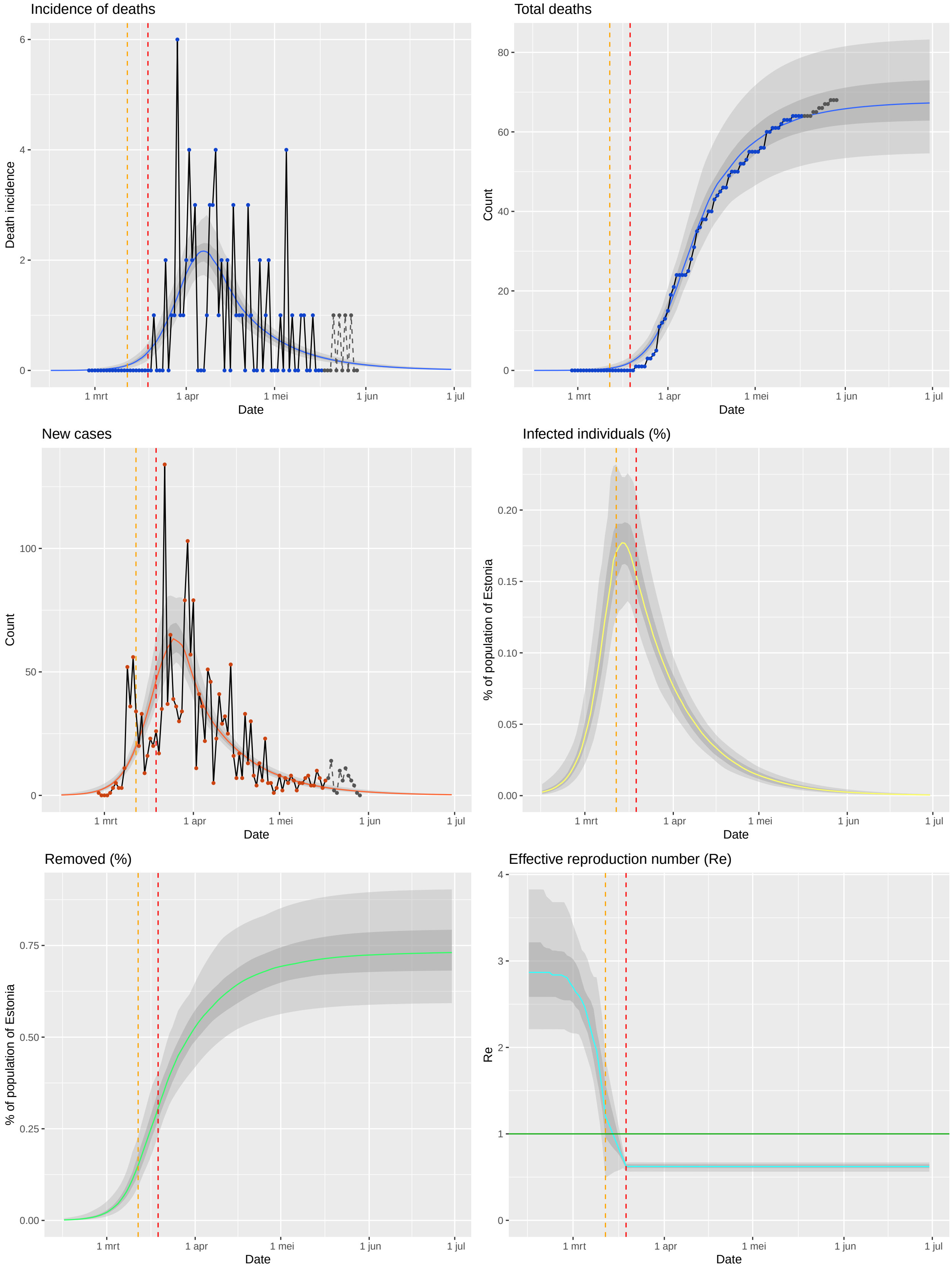

Incidence of deaths
Death incidence
6
4
2
0
1 mrt
1 apr
1 mei
1 jun
1 jul
Date
Total deaths
Count
80
60
40
20
0
1 mrt
1 apr
1 mei
1 jun
1 jul
Date
New cases
Count
100
50
0
1 mrt
1 apr
1 mei
1 jun
1 jul
Date
Infected individuals (%)
% of population of Estonia
0.20
0.15
0.10
0.05
0.00
1 mrt
1 apr
1 mei
1 jun
1 jul
Date
Removed (%)
% of population of Estonia
0.75
0.50
0.25
0.00
1 mrt
1 apr
1 mei
1 jun
1 jul
Date
Effective reproduction number (Re)
Re
4
3
2
1
0
1 mrt
1 apr
1 mei
1 jun
1 jul
Date

10 Finland

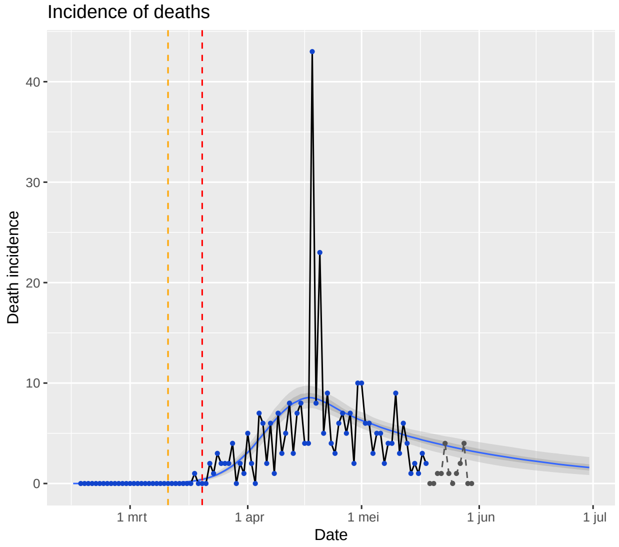
Incidence of deaths
Death incidence
0
10
20
30
40
1 mrt
1 apr
1 mei
1 jun
1 jul
Date

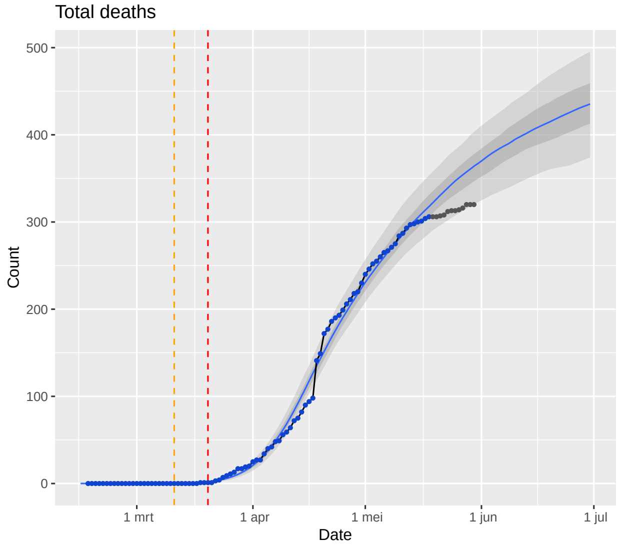
Total deaths
Count
0
100
200
300
400
500
1 mrt
1 apr
1 mei
1 jun
1 jul
Date

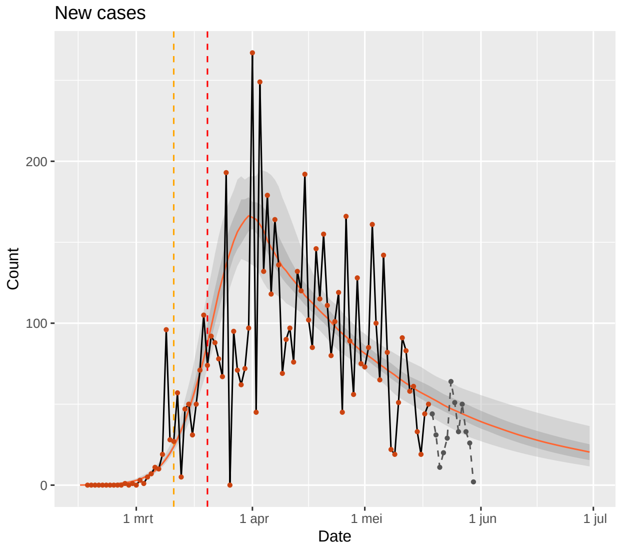
New cases
Count
0
100
200
1 mrt
1 apr
1 mei
1 jun
1 jul
Date

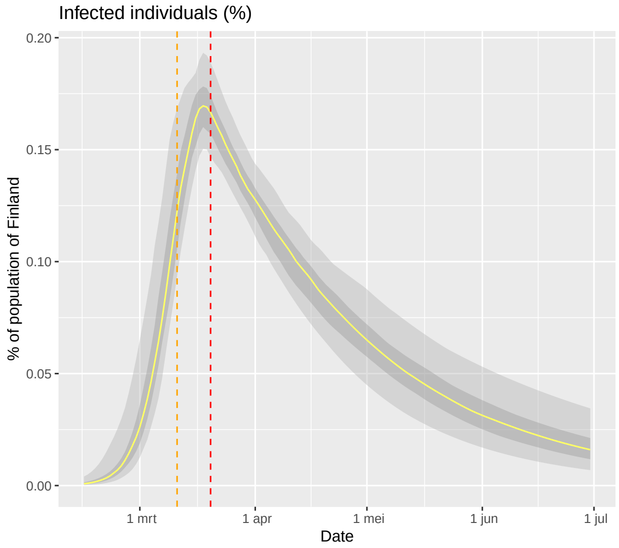
Infected individuals (%)
% of population of Finland
0.00
0.05
0.10
0.15
0.20
1 mrt
1 apr
1 mei
1 jun
1 jul
Date

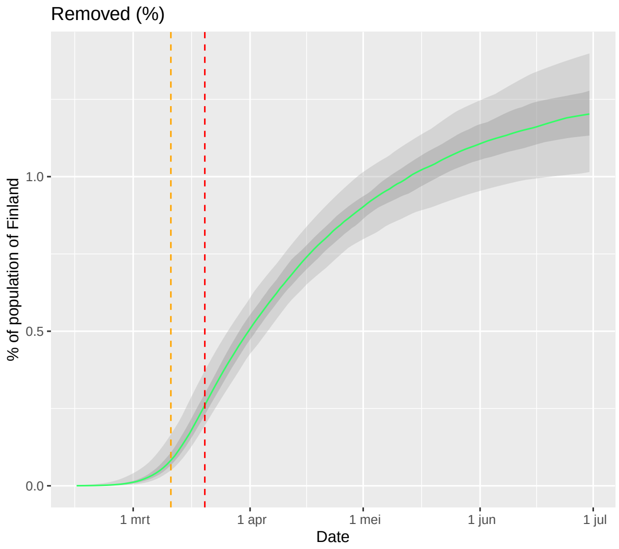
Removed (%)
% of population of Finland
0.0
0.5
1.0
1 mrt
1 apr
1 mei
1 jun
1 jul
Date

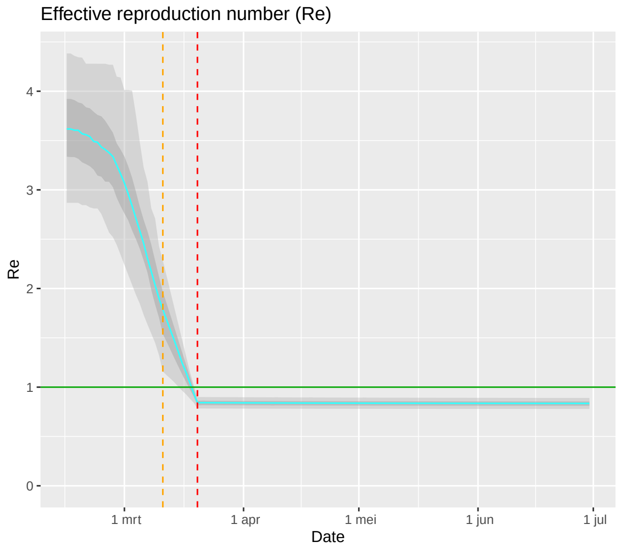
Effective reproduction number (Re)
Re
0
1
2
3
4
1 mrt
1 apr
1 mei
1 jun
1 jul
Date

11 France

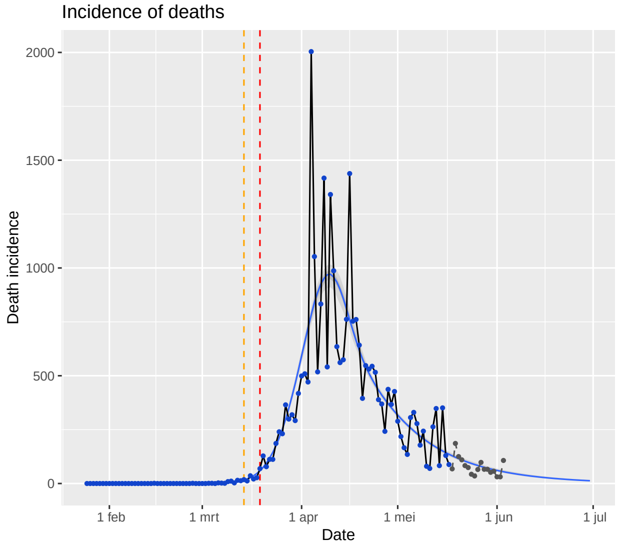
Incidence of deaths
2000
1500
1000
500
0
Death incidence
1 feb
1 mrt
1 apr
1 mei
1 jun
1 jul
Date

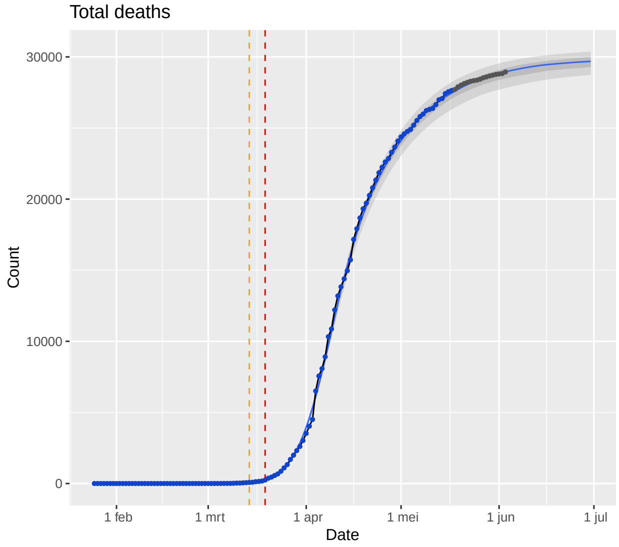
Total deaths
30000
20000
10000
0
Count
1 feb
1 mrt
1 apr
1 mei
1 jun
1 jul
Date

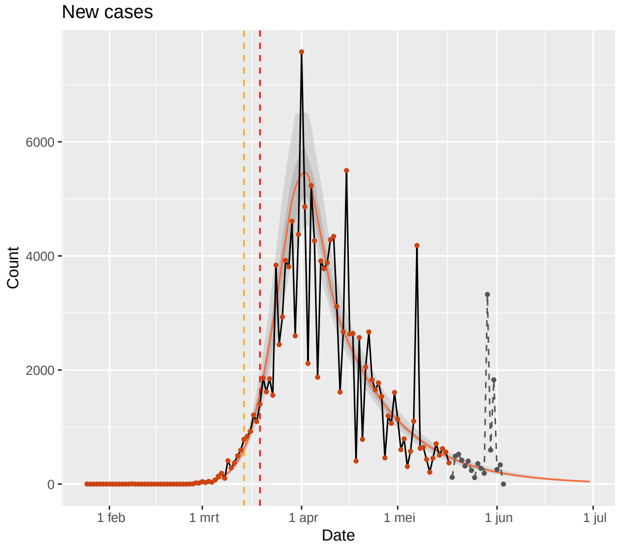
New cases
6000
4000
2000
0
Count
1 feb
1 mrt
1 apr
1 mei
1 jun
1 jul
Date

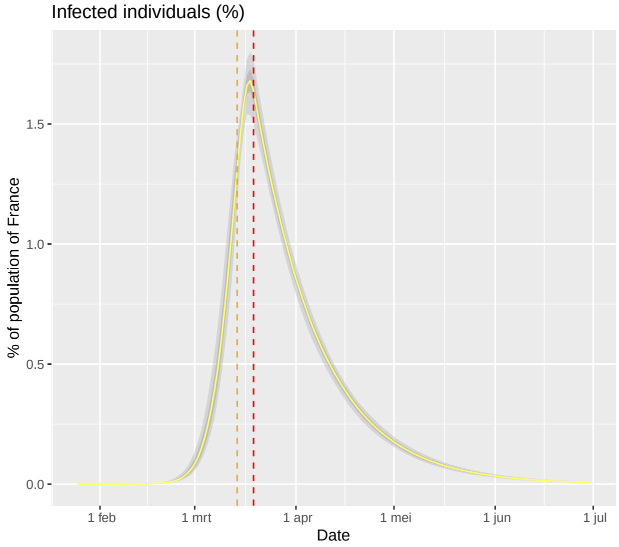
Infected individuals (%)
1.5
1.0
0.5
0.0
% of population of France
1 feb
1 mrt
1 apr
1 mei
1 jun
1 jul
Date

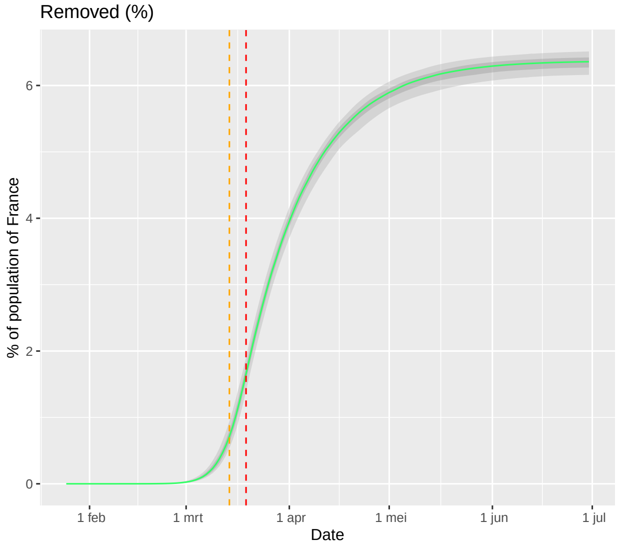
Removed (%)
6
4
2
0
% of population of France
1 feb
1 mrt
1 apr
1 mei
1 jun
1 jul
Date

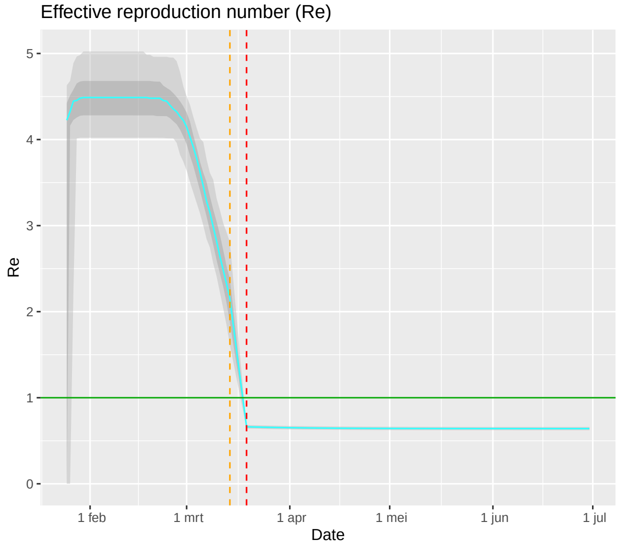
Effective reproduction number (Re)
5
4
3
2
1
0
Re
1 feb
1 mrt
1 apr
1 mei
1 jun
1 jul
Date

12 Germany

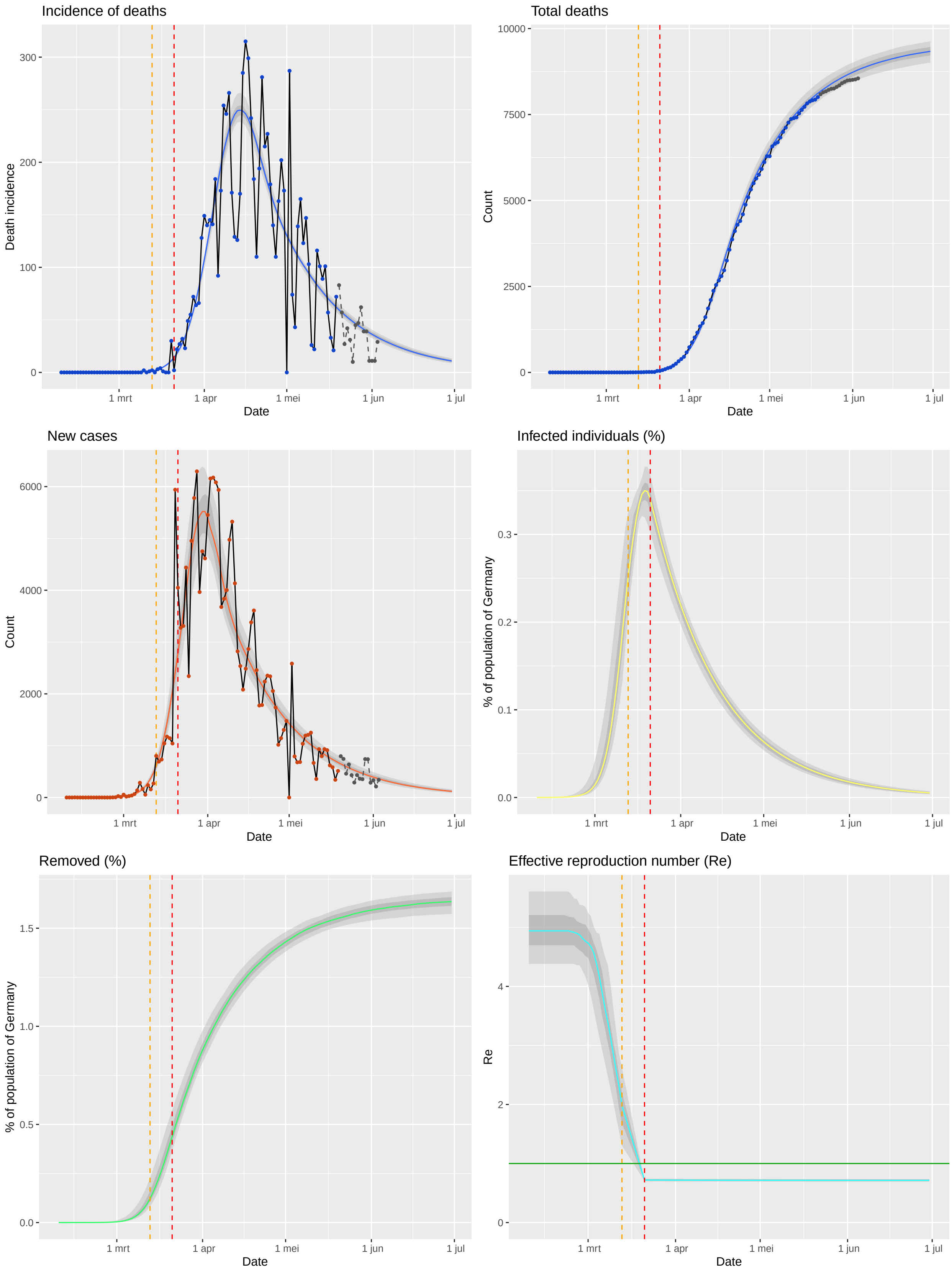
Incidence of deaths
Death incidence
0
100
200
300
1 mrt
1 apr
1 mei
1 jun
1 jul
Date
Total deaths
Count
0
2500
5000
7500
10000
1 mrt
1 apr
1 mei
1 jun
1 jul
Date
New cases
Count
0
2000
4000
6000
1 mrt
1 apr
1 mei
1 jun
1 jul
Date
Infected individuals (%)
% of population of Germany
0.0
0.1
0.2
0.3
1 mrt
1 apr
1 mei
1 jun
1 jul
Date
Removed (%)
% of population of Germany
0.0
0.5
1.0
1.5
1 mrt
1 apr
1 mei
1 jun
1 jul
Date
Effective reproduction number (Re)
Re
0
2
4
1 mrt
1 apr
1 mei
1 jun
1 jul
Date

13 Greece

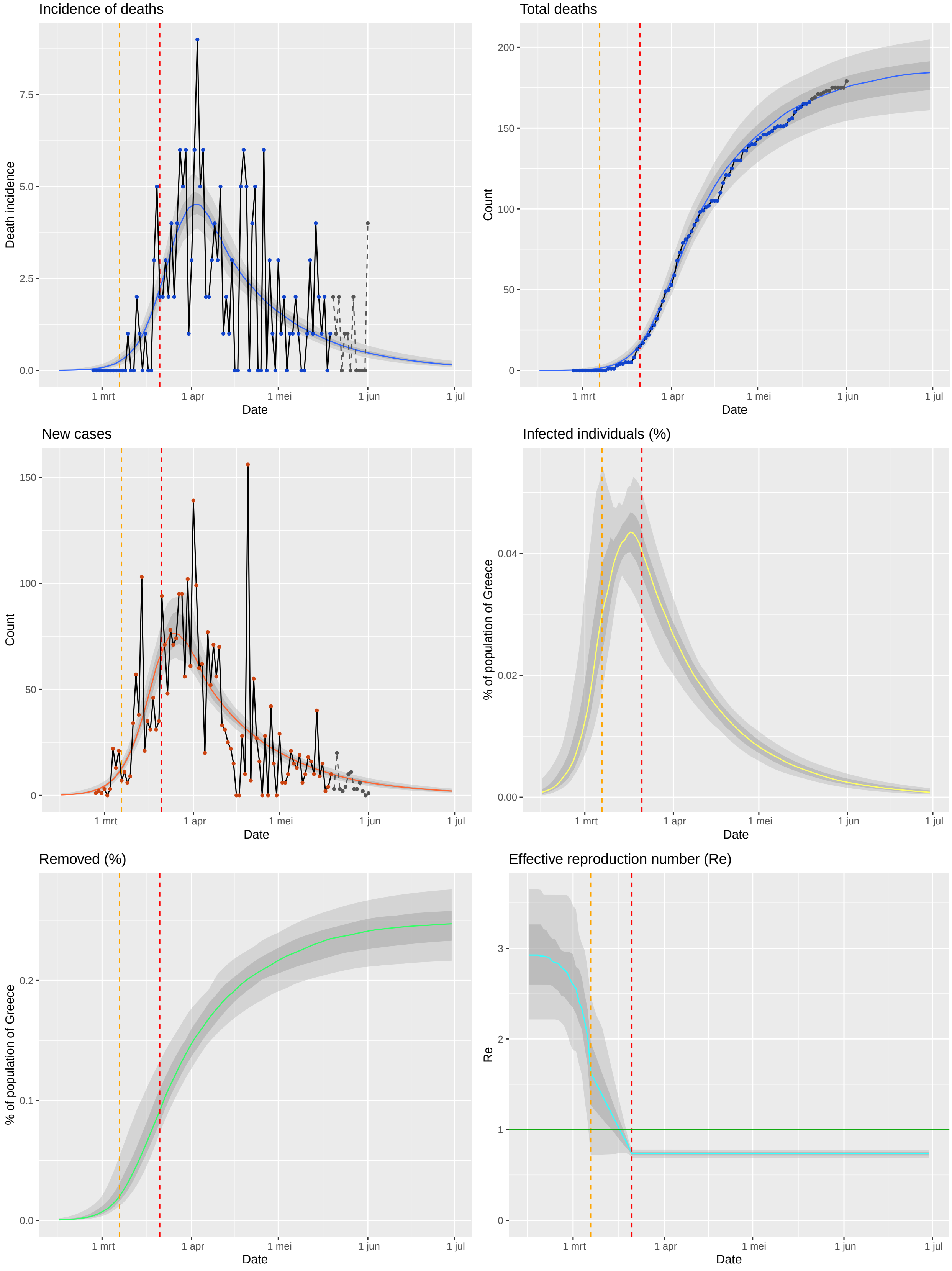
Incidence of deaths
Death incidence
0.0
2.5
5.0
7.5
1 mrt
1 apr
1 mei
1 jun
1 jul
Date
Total deaths
Count
0
50
100
150
200
1 mrt
1 apr
1 mei
1 jun
1 jul
Date
New cases
Count
0
50
100
150
1 mrt
1 apr
1 mei
1 jun
1 jul
Date
Infected individuals (%)
% of population of Greece
0.00
0.02
0.04
1 mrt
1 apr
1 mei
1 jun
1 jul
Date
Removed (%)
% of population of Greece
0.0
0.1
0.2
1 mrt
1 apr
1 mei
1 jun
1 jul
Date
Effective reproduction number (Re)
Re
0
1
2
3
1 mrt
1 apr
1 mei
1 jun
1 jul
Date

14 Hungary

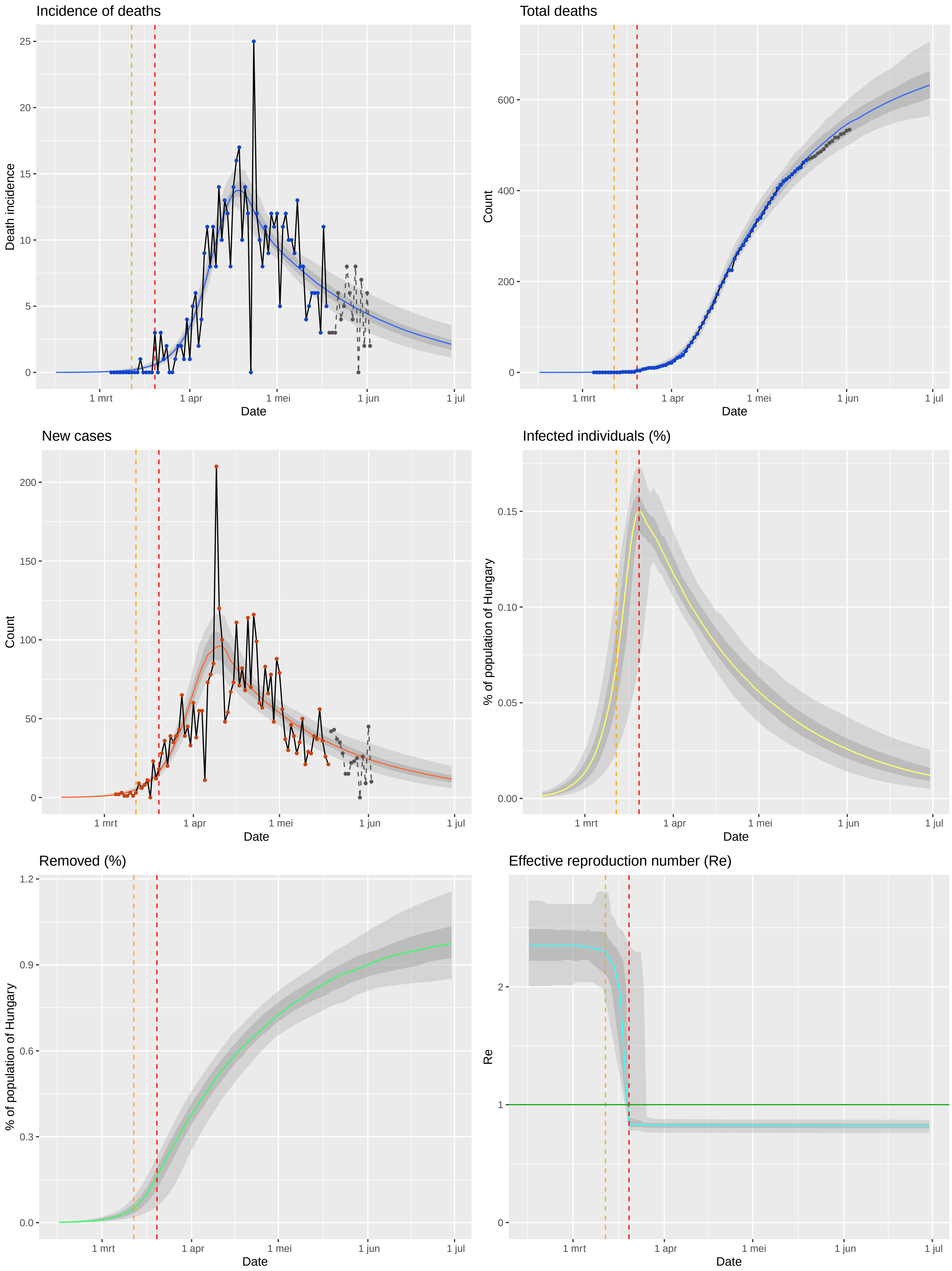
Incidence of deaths
Death incidence
Date
1 mrt
1 apr
1 mei
1 jun
1 jul
Total deaths
Count
Date
New cases
Count
Date
Infected individuals (%)
% of population of Hungary
Date
Removed (%)
% of population of Hungary
Date
Effective reproduction number (Re)
Re
Date

15 Ireland

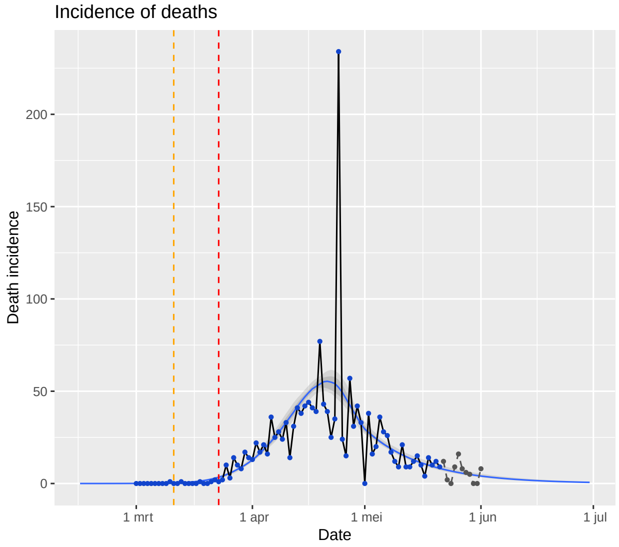
Incidence of deaths
Death incidence
0
50
100
150
200
1 mrt
1 apr
1 mei
1 jun
1 jul
Date

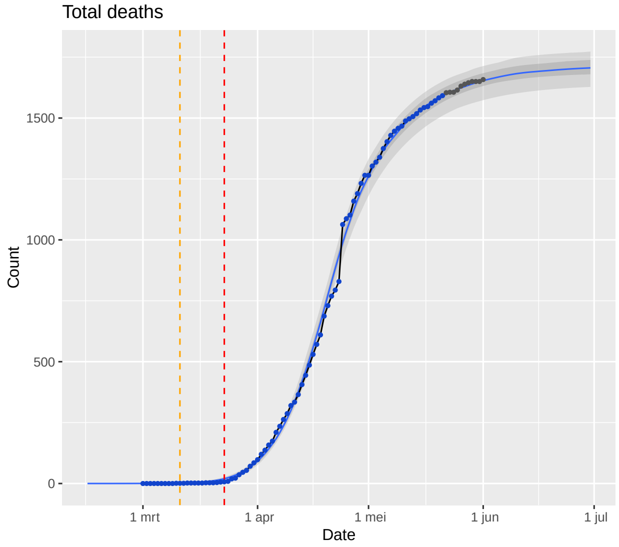
Total deaths
Count
0
500
1000
1500
1 mrt
1 apr
1 mei
1 jun
1 jul
Date

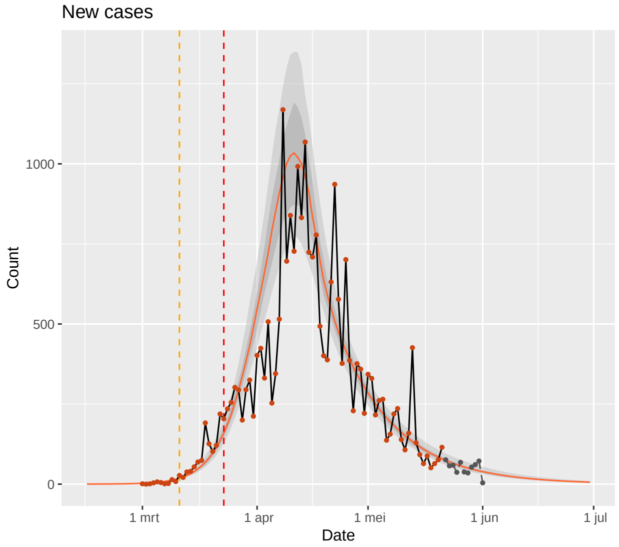
New cases
Count
0
500
1000
1 mrt
1 apr
1 mei
1 jun
1 jul
Date

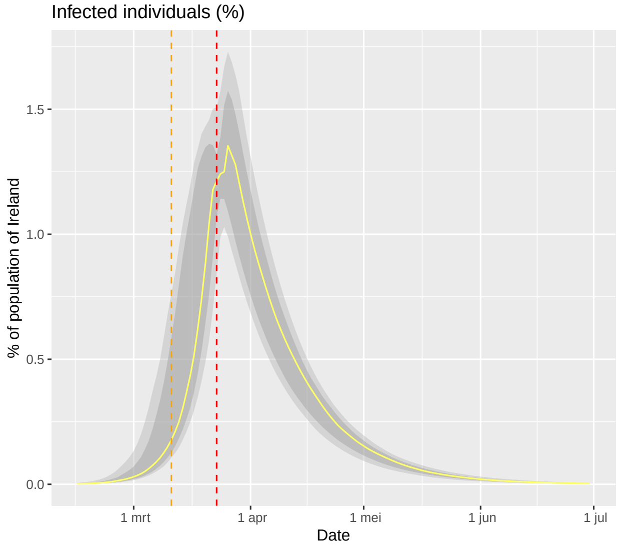
Infected individuals (%)
% of population of Ireland
0.0
0.5
1.0
1.5
1 mrt
1 apr
1 mei
1 jun
1 jul
Date

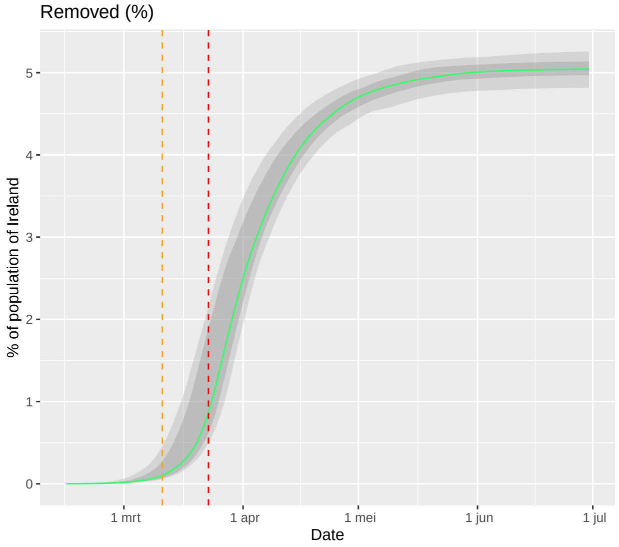
Removed (%)
% of population of Ireland
0
1
2
3
4
5
1 mrt
1 apr
1 mei
1 jun
1 jul
Date

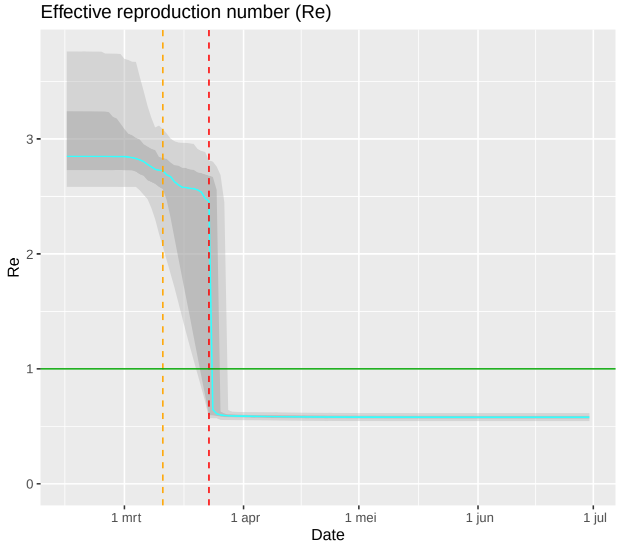
Effective reproduction number (Re)
Re
0
1
2
3
1 mrt
1 apr
1 mei
1 jun
1 jul
Date

16 Israel

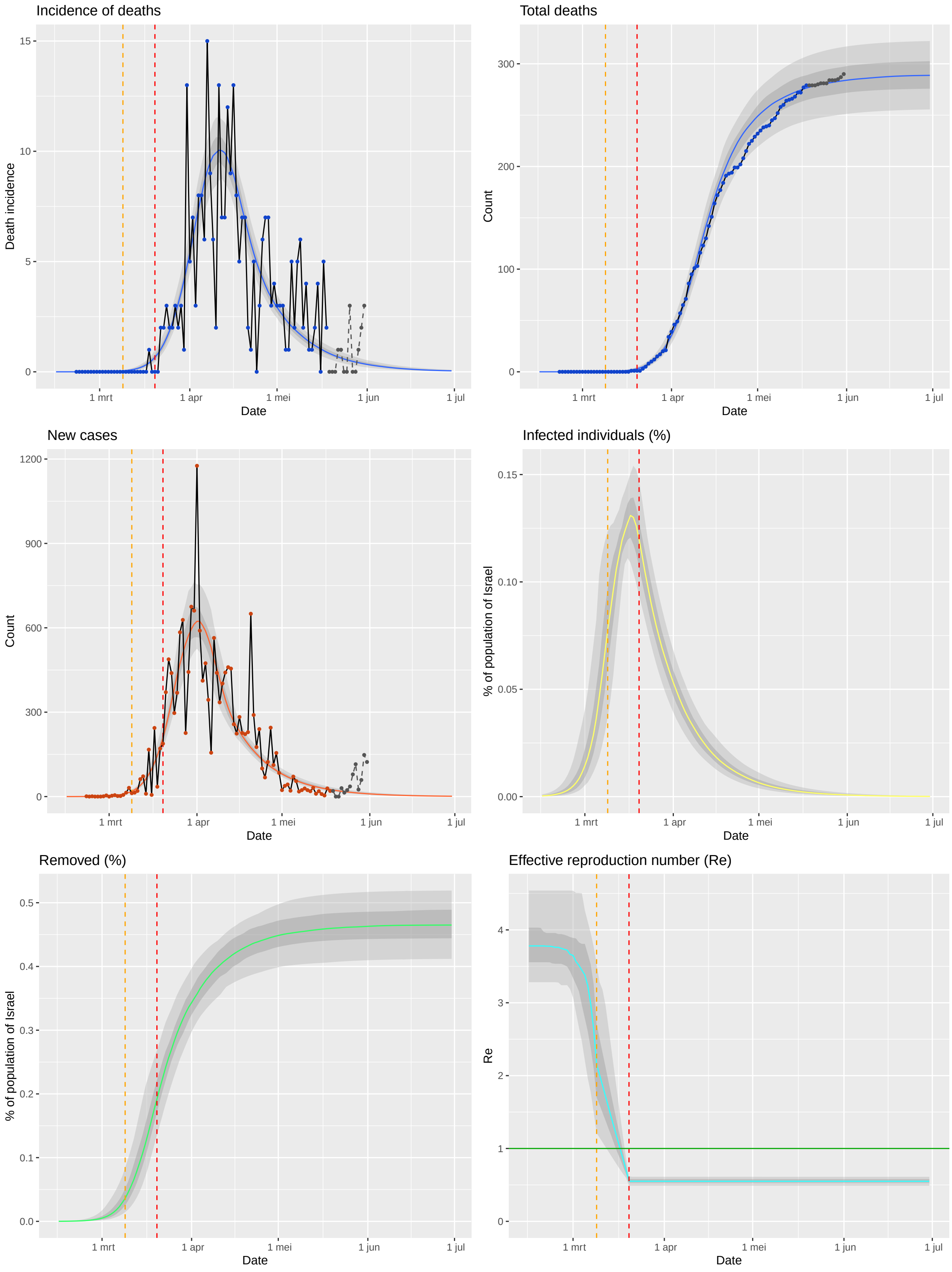
Incidence of deaths
Death incidence
Date
1 mrt
1 apr
1 mei
1 jun
1 jul
Total deaths
Count
Date
New cases
Count
Date
Infected individuals (%)
% of population of Israel
Date
Removed (%)
% of population of Israel
Date
Effective reproduction number (Re)
Re
Date

17 Italy

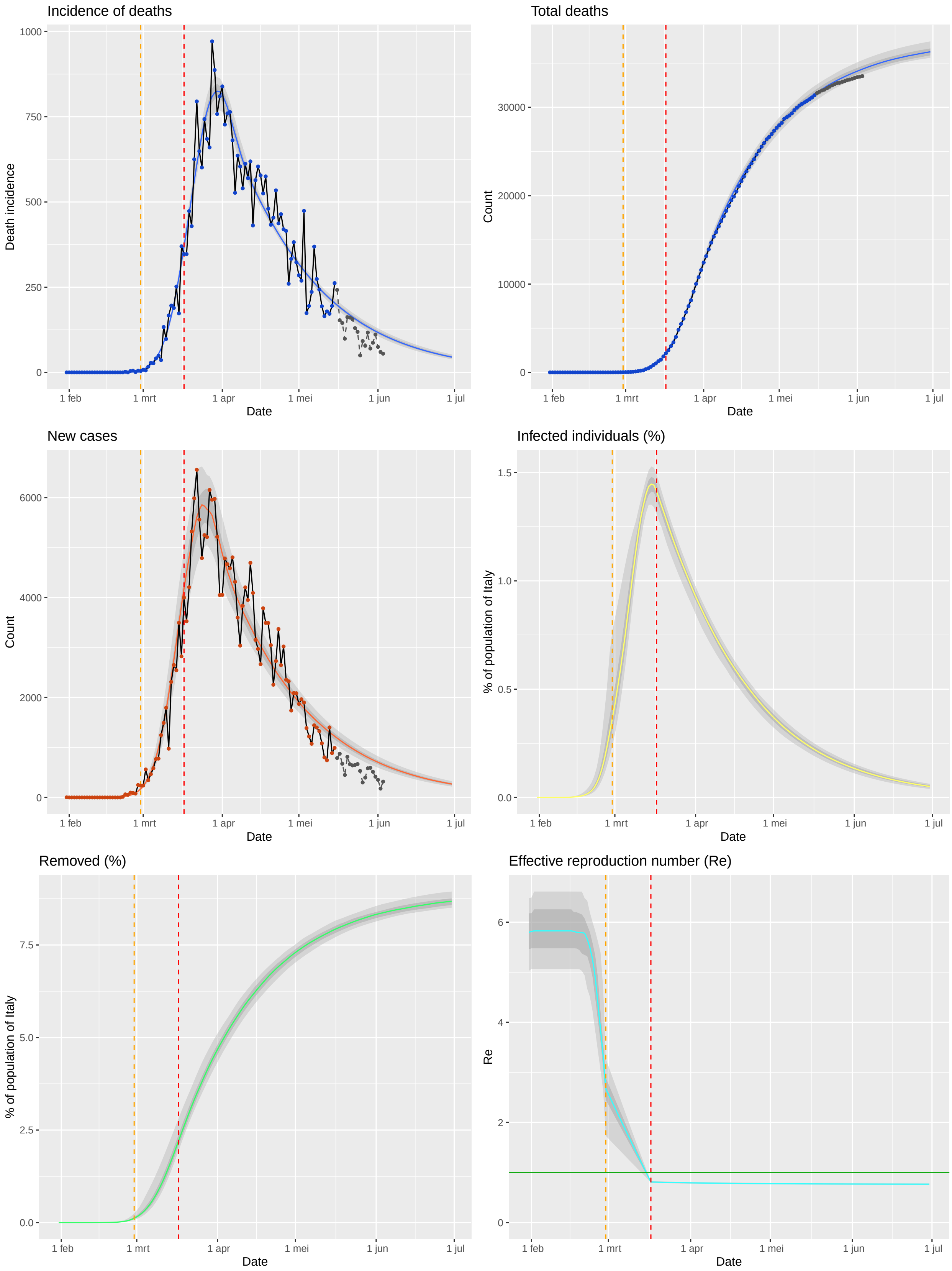
Incidence of deaths
Death incidence
Date
Total deaths
Count
Date
New cases
Count
Date
Infected individuals (%)
% of population of Italy
Date
Removed (%)
% of population of Italy
Date
Effective reproduction number (Re)
Re
Date
1 feb
1 mrt
1 apr
1 mei
1 jun
1 jul

18 Latvia

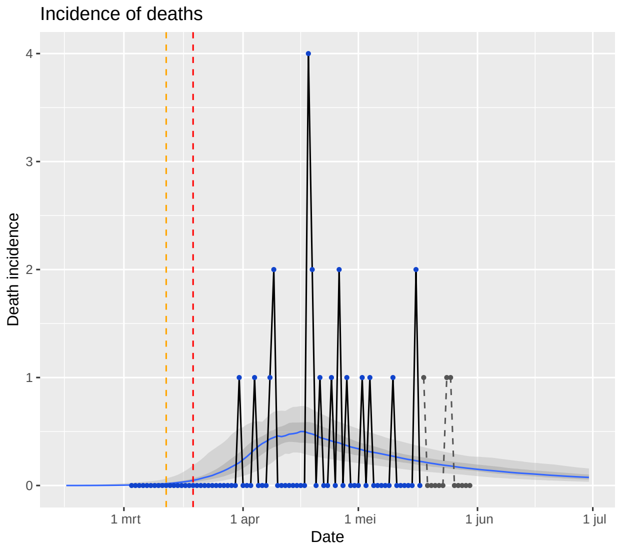
Incidence of deaths
Death incidence
0
1
2
3
4
1 mrt
1 apr
1 mei
1 jun
1 jul
Date

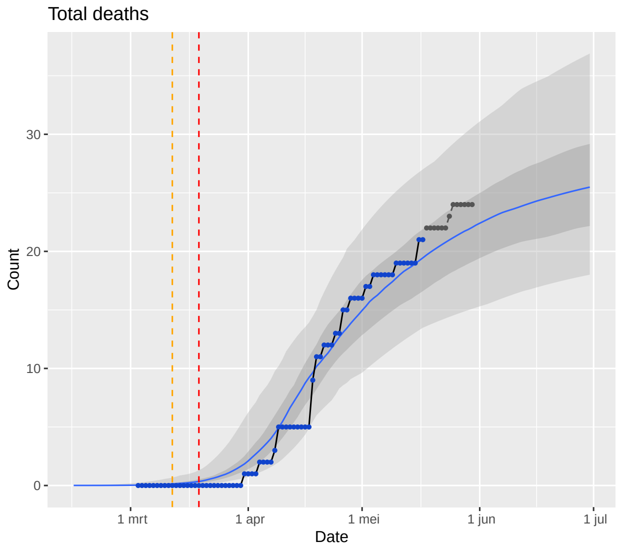
Total deaths
Count
0
10
20
30
1 mrt
1 apr
1 mei
1 jun
1 jul
Date

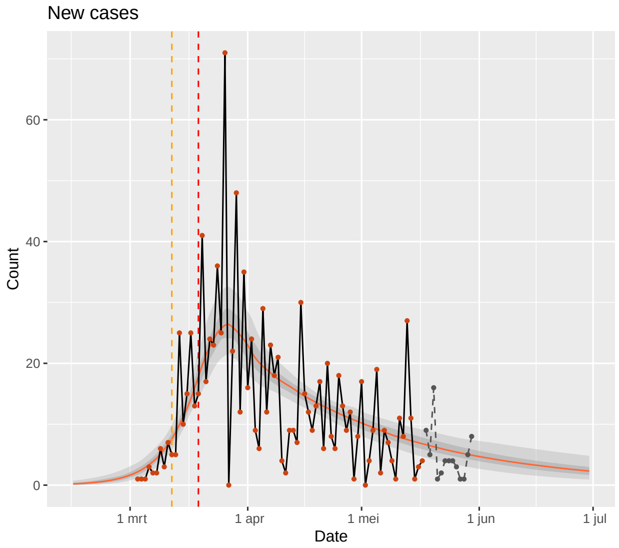
New cases
Count
0
20
40
60
1 mrt
1 apr
1 mei
1 jun
1 jul
Date

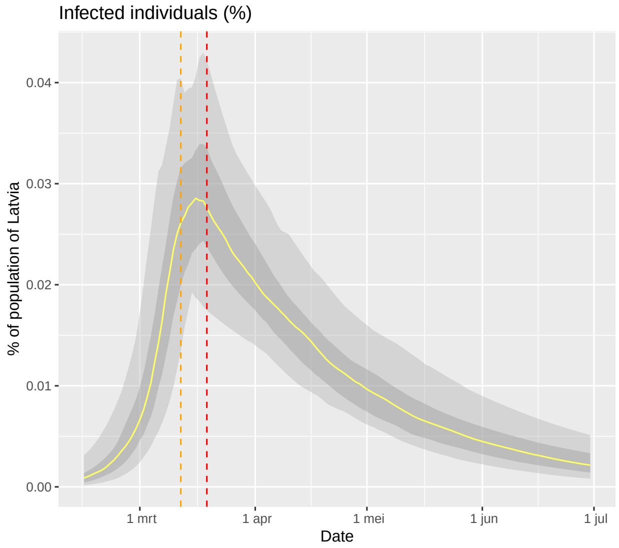
Infected individuals (%)
% of population of Latvia
0.00
0.01
0.02
0.03
0.04
1 mrt
1 apr
1 mei
1 jun
1 jul
Date

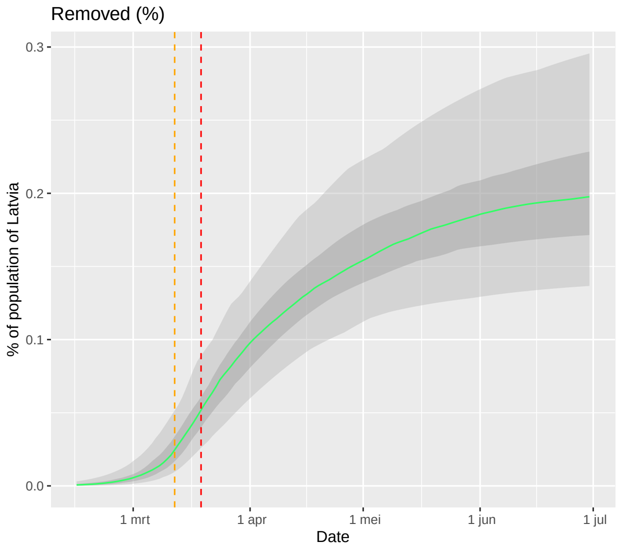
Removed (%)
% of population of Latvia
0.0
0.1
0.2
0.3
1 mrt
1 apr
1 mei
1 jun
1 jul
Date

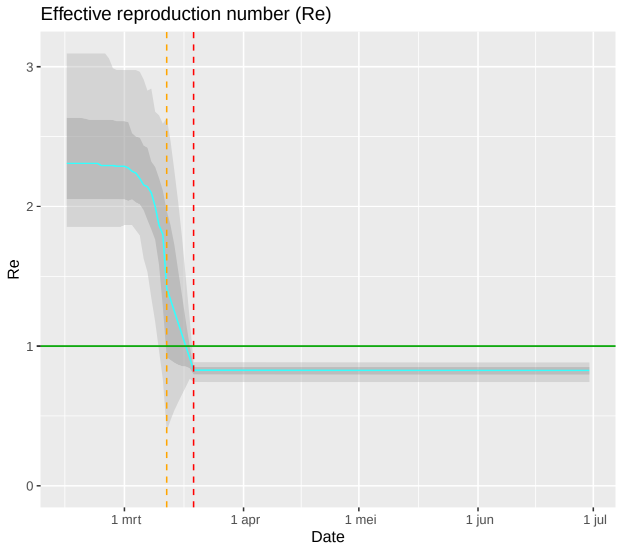
Effective reproduction number (Re)
Re
0
1
2
3
1 mrt
1 apr
1 mei
1 jun
1 jul
Date

19 Lithuania

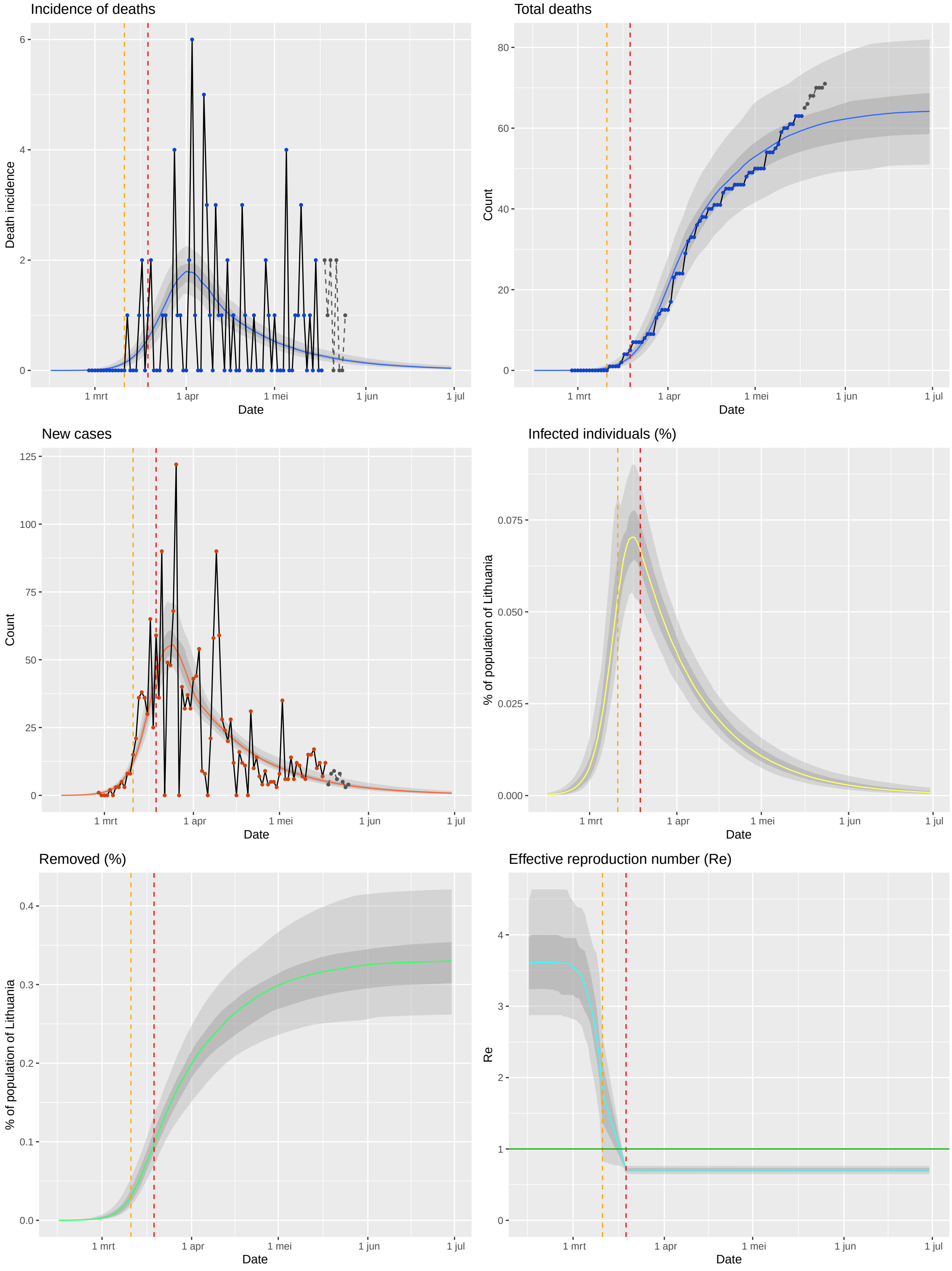

20 Luxembourg

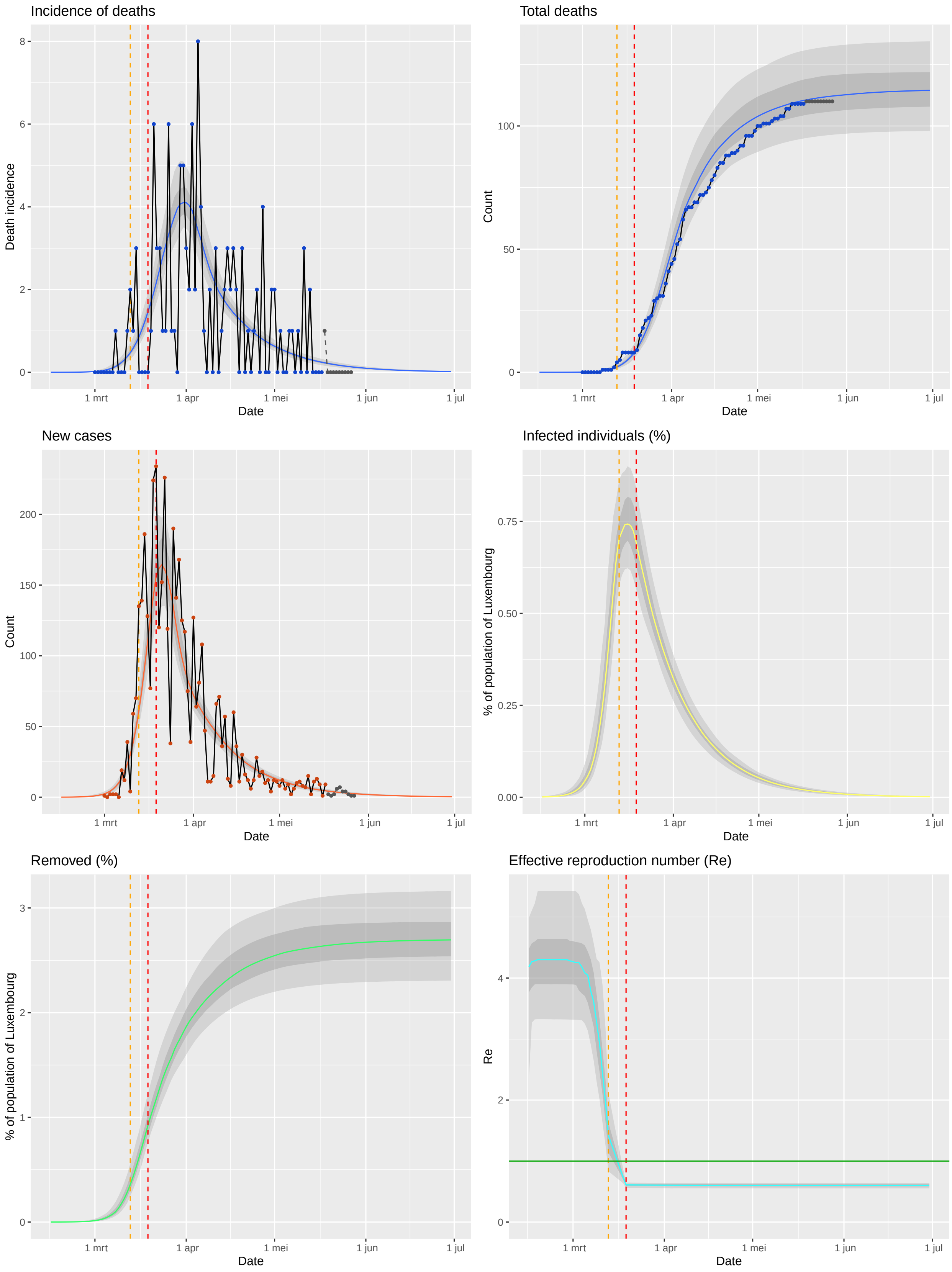

21 Moldova

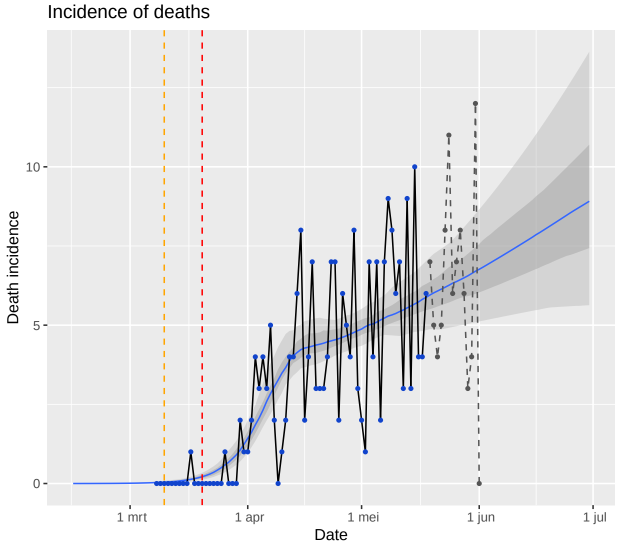
Incidence of deaths
Death incidence
0
5
10
1 mrt
1 apr
1 mei
1 jun
1 jul
Date

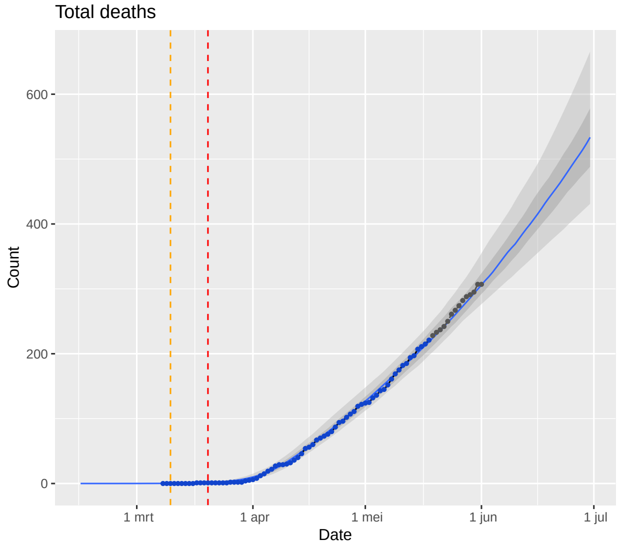
Total deaths
Count
0
200
400
600
1 mrt
1 apr
1 mei
1 jun
1 jul
Date

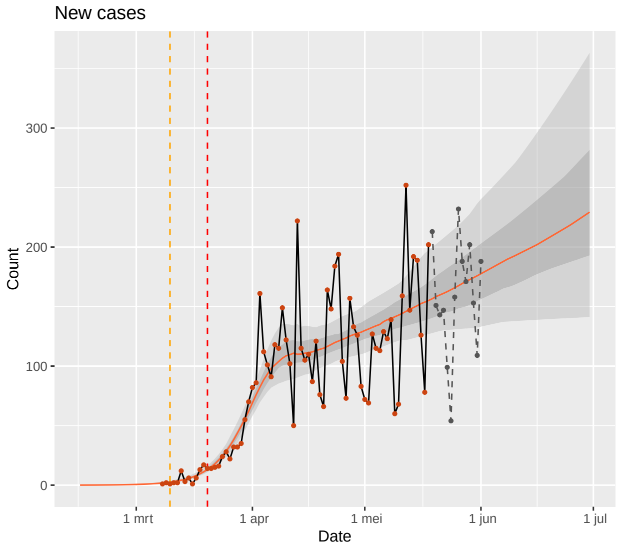
New cases
Count
0
100
200
300
1 mrt
1 apr
1 mei
1 jun
1 jul
Date

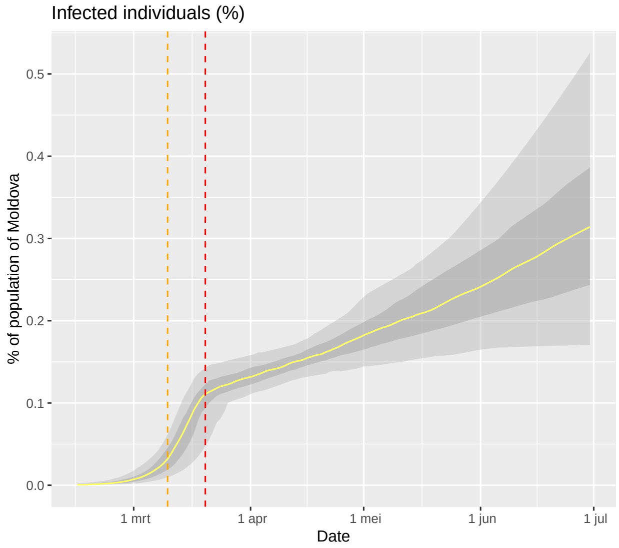
Infected individuals (%)
% of population of Moldova
0.0
0.1
0.2
0.3
0.4
0.5
1 mrt
1 apr
1 mei
1 jun
1 jul
Date

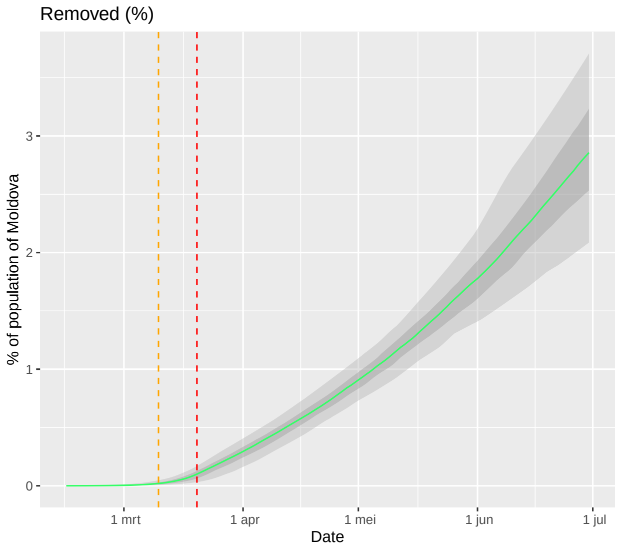
Removed (%)
% of population of Moldova
0
1
2
3
1 mrt
1 apr
1 mei
1 jun
1 jul
Date

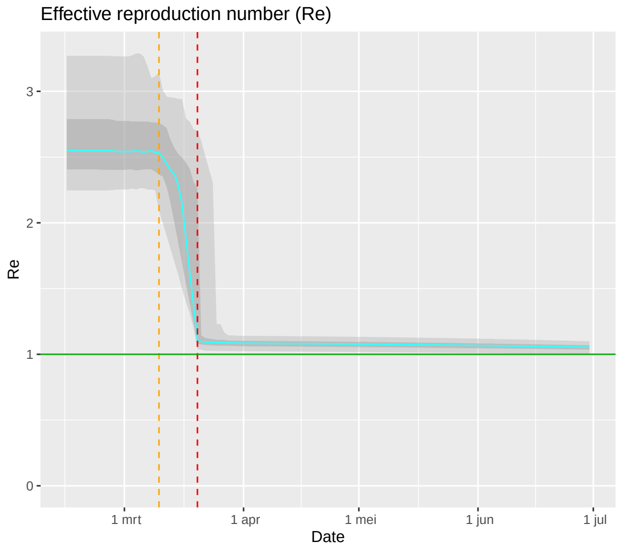
Effective reproduction number (Re)
Re
0
1
2
3
1 mrt
1 apr
1 mei
1 jun
1 jul
Date

22 Netherlands

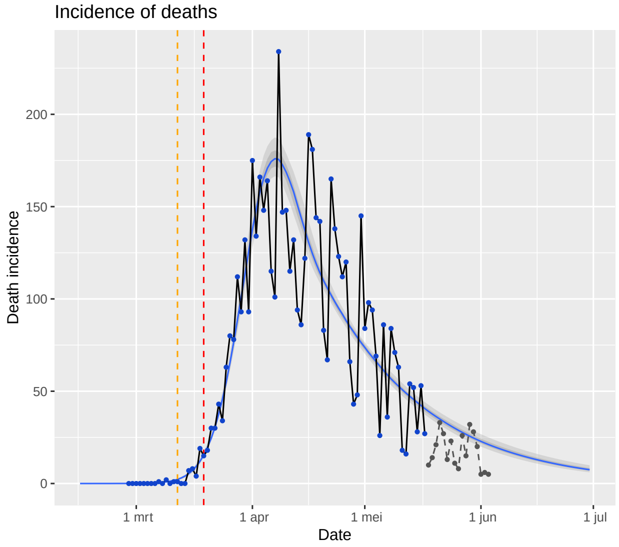

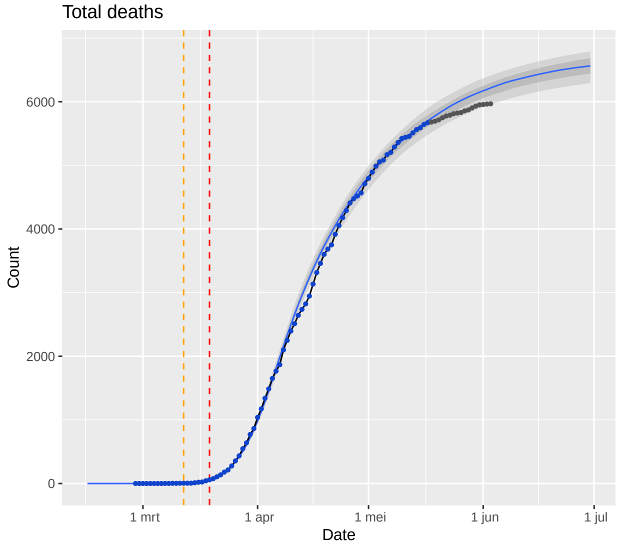

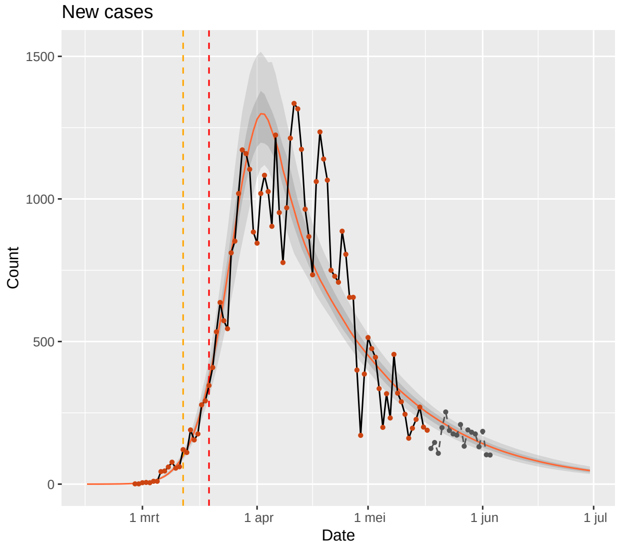

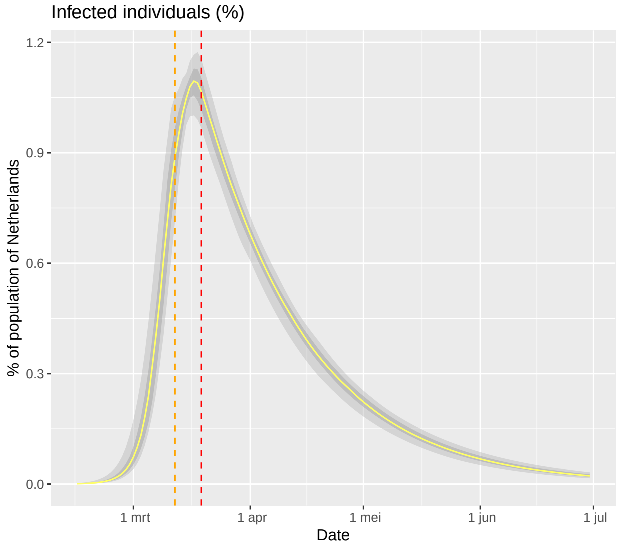

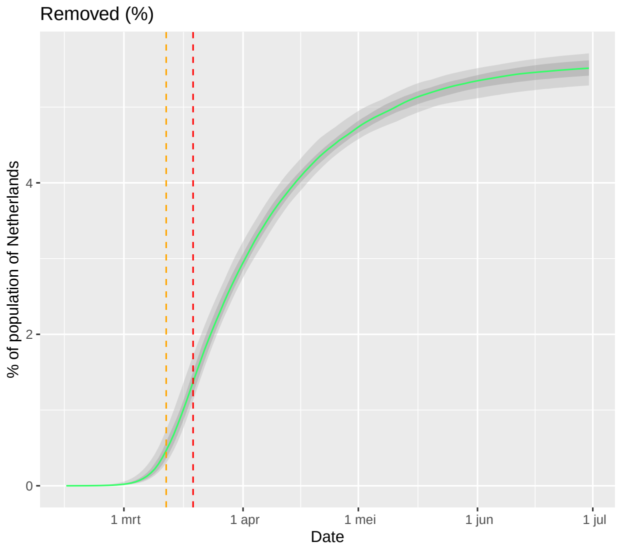

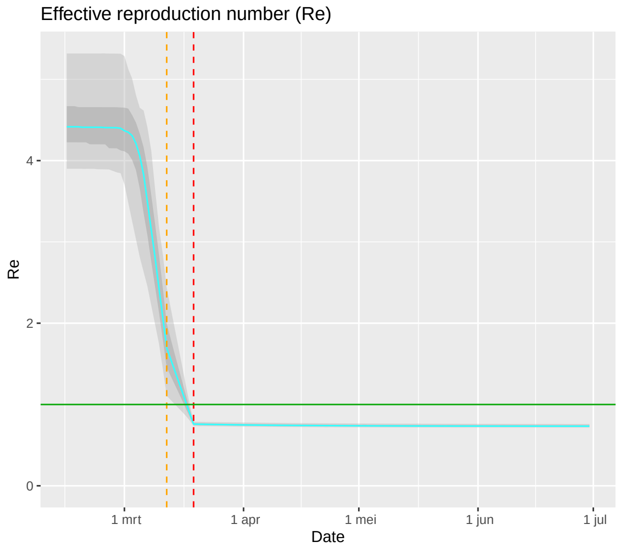

23 North Macedonia

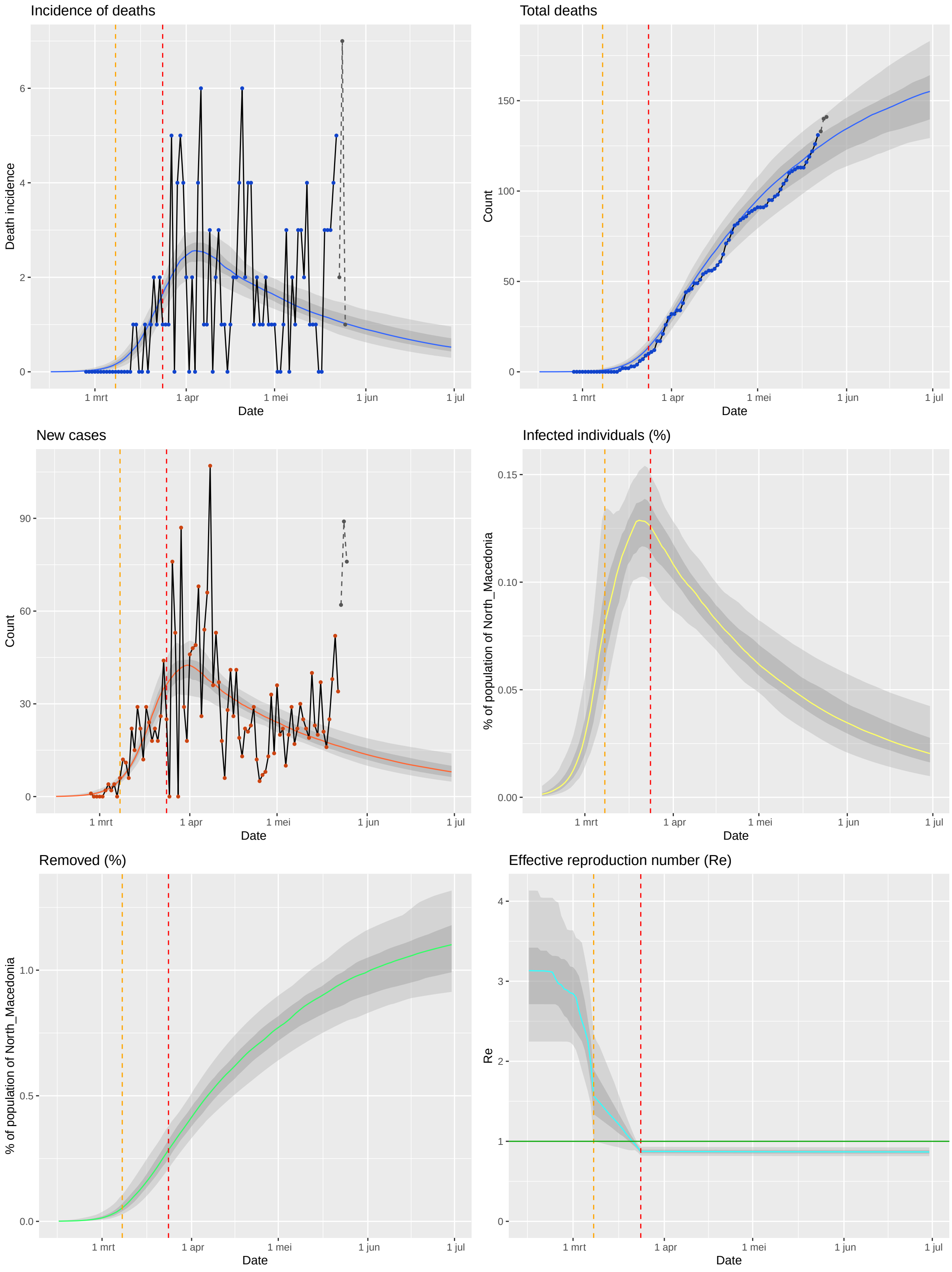

24 Norway

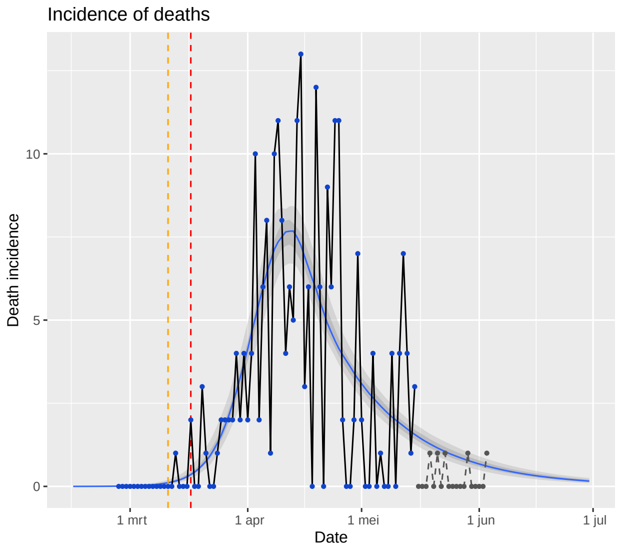
Incidence of deaths
Death incidence
0
5
10
1 mrt
1 apr
1 mei
1 jun
1 jul
Date

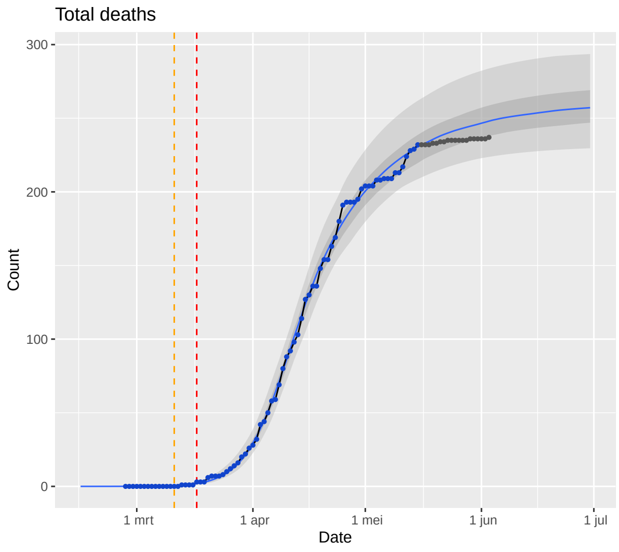
Total deaths
Count
0
100
200
300
1 mrt
1 apr
1 mei
1 jun
1 jul
Date

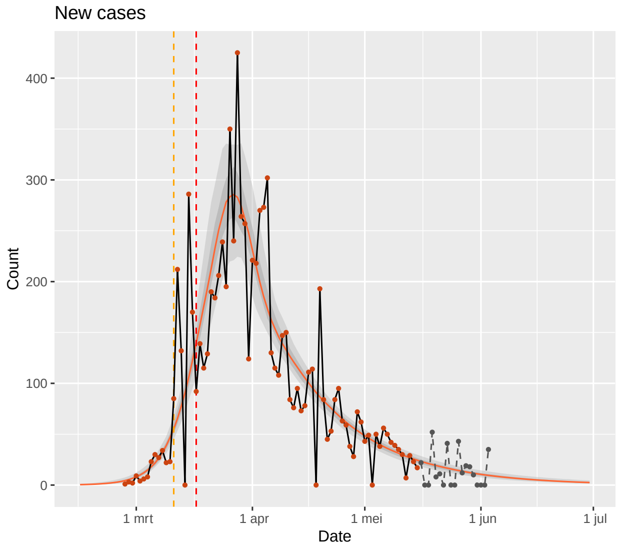
New cases
Count
0
100
200
300
400
1 mrt
1 apr
1 mei
1 jun
1 jul
Date

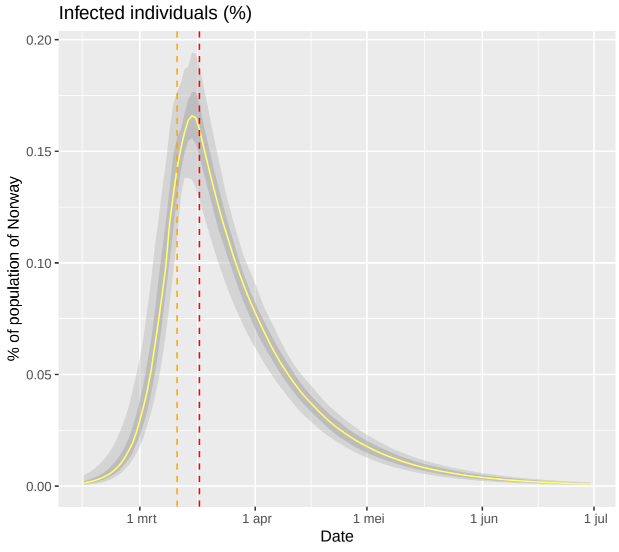
Infected individuals (%)
% of population of Norway
0.00
0.05
0.10
0.15
0.20
1 mrt
1 apr
1 mei
1 jun
1 jul
Date

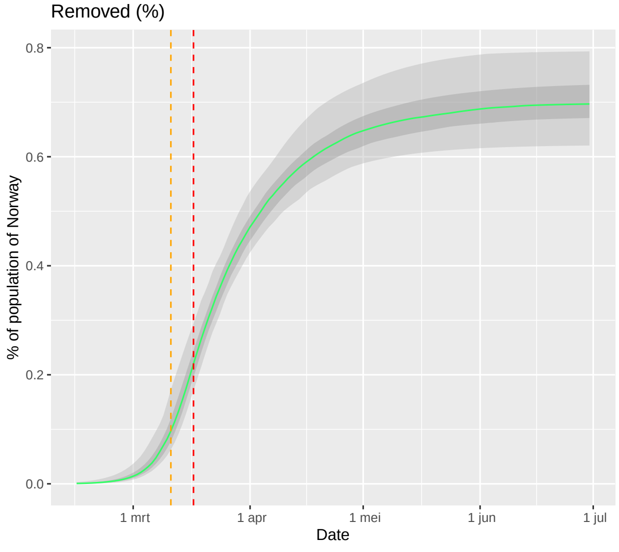
Removed (%)
% of population of Norway
0.0
0.2
0.4
0.6
0.8
1 mrt
1 apr
1 mei
1 jun
1 jul
Date

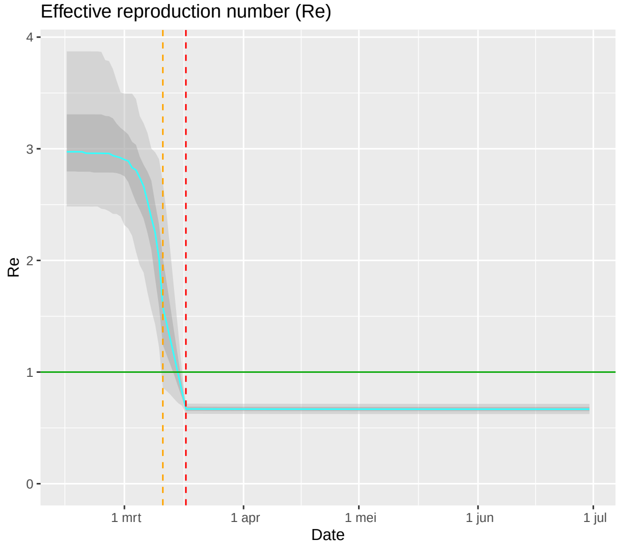
Effective reproduction number (Re)
Re
0
1
2
3
4
1 mrt
1 apr
1 mei
1 jun
1 jul
Date

25 Poland

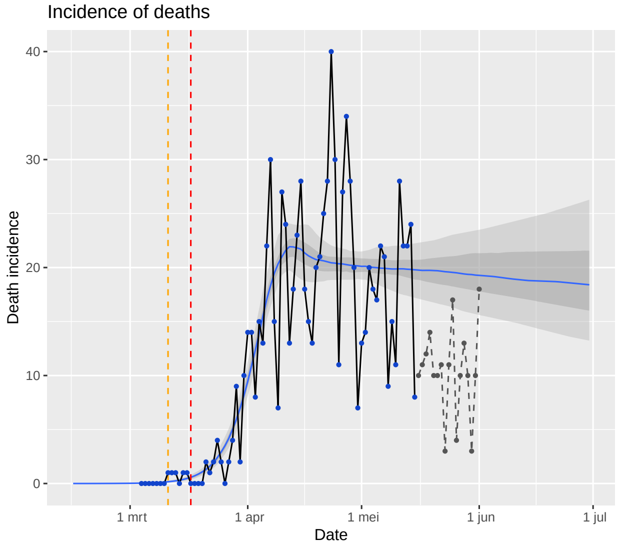
Incidence of deaths
Death incidence
0
10
20
30
40
1 mrt
1 apr
1 mei
1 jun
1 jul
Date

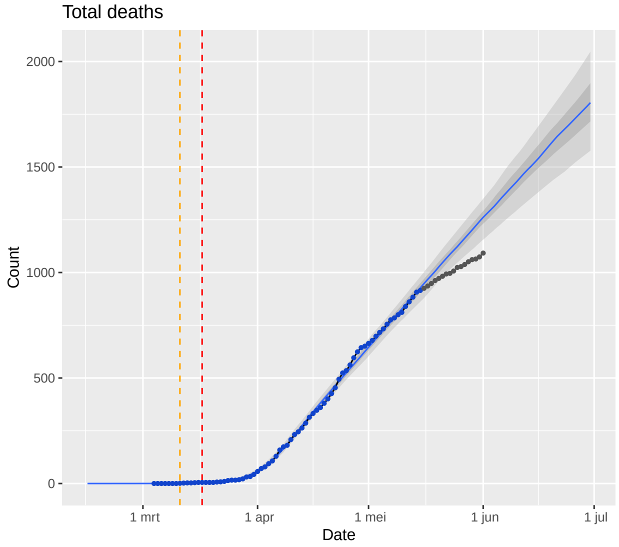
Total deaths
Count
0
500
1000
1500
2000
1 mrt
1 apr
1 mei
1 jun
1 jul
Date

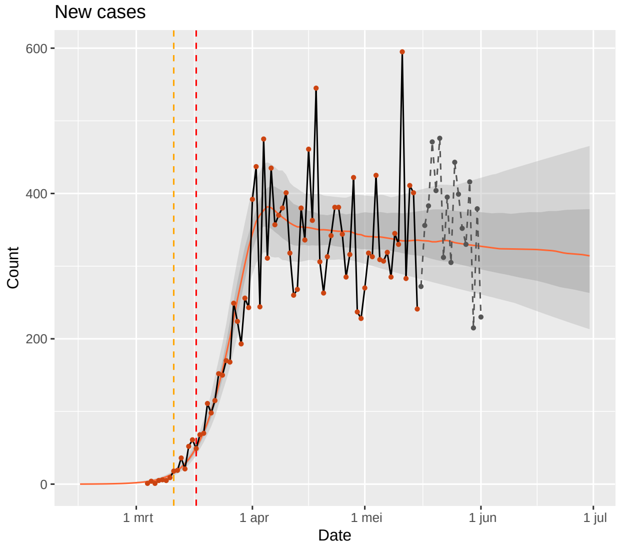
New cases
Count
0
200
400
600
1 mrt
1 apr
1 mei
1 jun
1 jul
Date

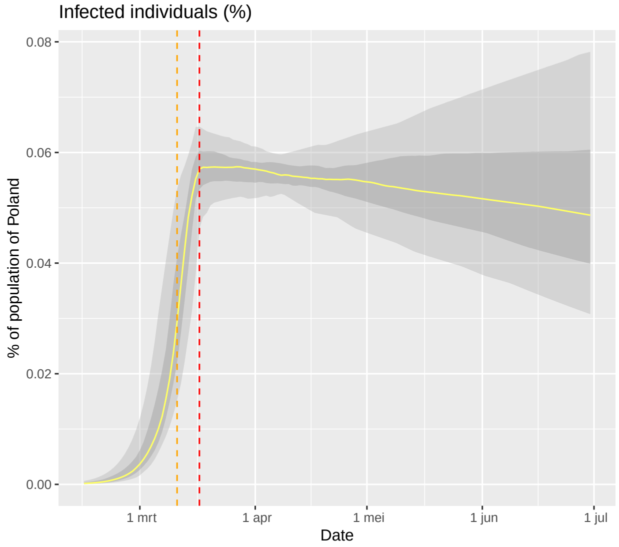
Infected individuals (%)
% of population of Poland
0.00
0.02
0.04
0.06
0.08
1 mrt
1 apr
1 mei
1 jun
1 jul
Date

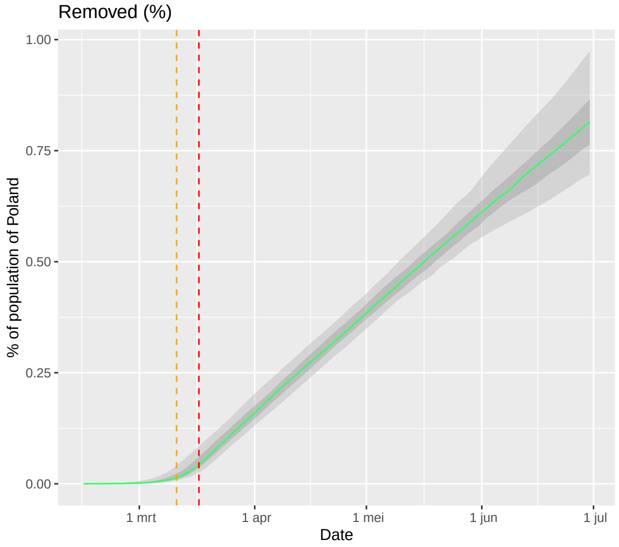
Removed (%)
% of population of Poland
0.00
0.25
0.50
0.75
1.00
1 mrt
1 apr
1 mei
1 jun
1 jul
Date

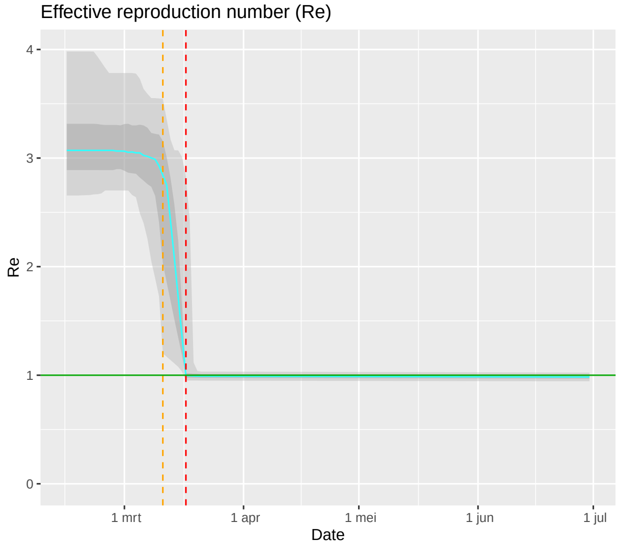
Effective reproduction number (Re)
Re
0
1
2
3
4
1 mrt
1 apr
1 mei
1 jun
1 jul
Date

26 Portugal

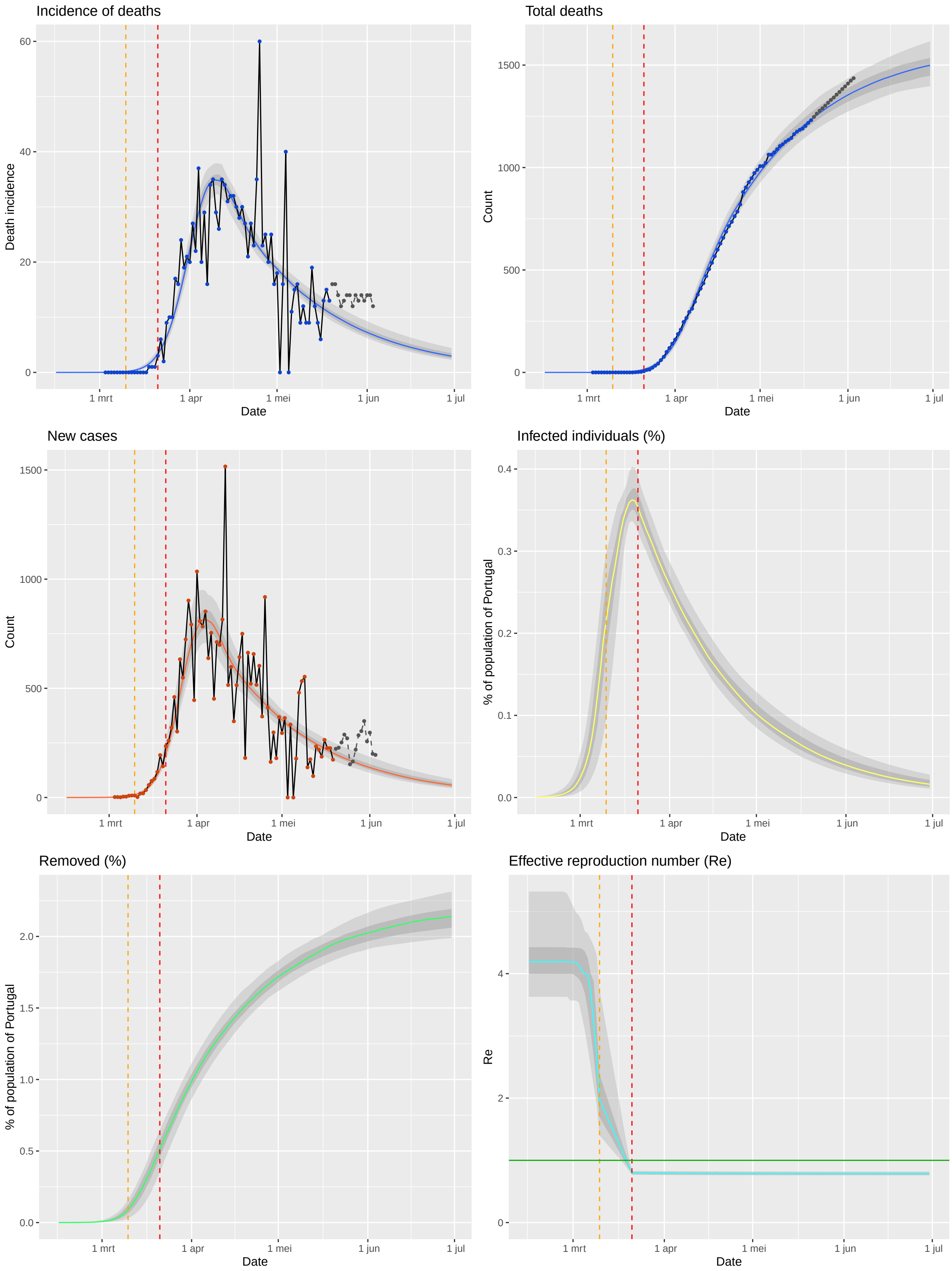
Incidence of deaths
Death incidence
0
20
40
60
1 mrt
1 apr
1 mei
1 jun
1 jul
Date
Total deaths
Count
0
500
1000
1500
1 mrt
1 apr
1 mei
1 jun
1 jul
Date
New cases
Count
0
500
1000
1500
1 mrt
1 apr
1 mei
1 jun
1 jul
Date
Infected individuals (%)
% of population of Portugal
0.0
0.1
0.2
0.3
0.4
1 mrt
1 apr
1 mei
1 jun
1 jul
Date
Removed (%)
% of population of Portugal
0.0
0.5
1.0
1.5
2.0
1 mrt
1 apr
1 mei
1 jun
1 jul
Date
Effective reproduction number (Re)
Re
0
2
4
1 mrt
1 apr
1 mei
1 jun
1 jul
Date

27 Romania

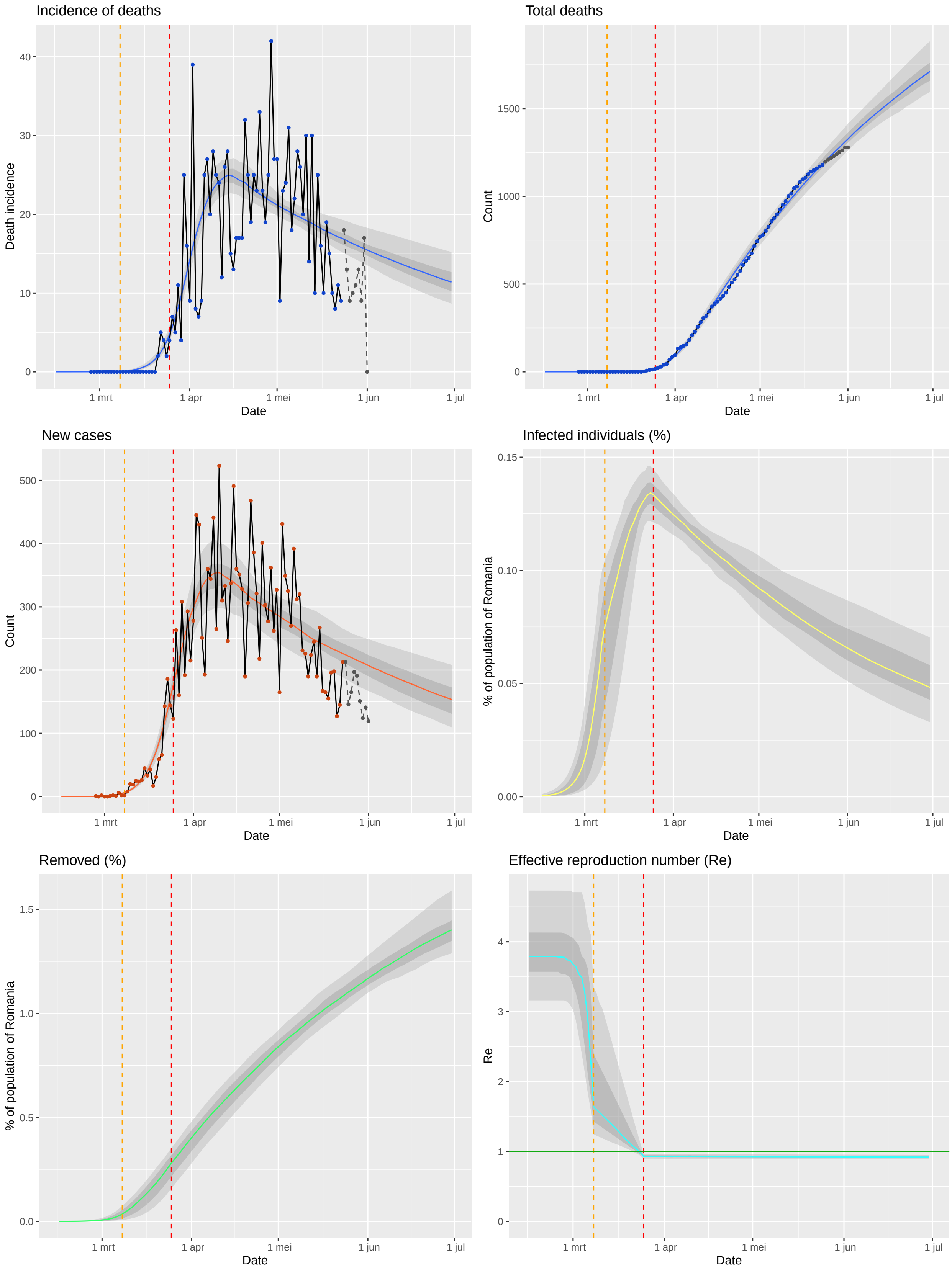
Incidence of deaths
Death incidence
40
30
20
10
0
1 mrt
1 apr
1 mei
1 jun
1 jul
Date
Total deaths
Count
1500
1000
500
0
1 mrt
1 apr
1 mei
1 jun
1 jul
Date
New cases
Count
500
400
300
200
100
0
1 mrt
1 apr
1 mei
1 jun
1 jul
Date
Infected individuals (%)
% of population of Romania
0.15
0.10
0.05
0.00
1 mrt
1 apr
1 mei
1 jun
1 jul
Date
Removed (%)
% of population of Romania
1.5
1.0
0.5
0.0
1 mrt
1 apr
1 mei
1 jun
1 jul
Date
Effective reproduction number (Re)
Re
4
3
2
1
0
1 mrt
1 apr
1 mei
1 jun
1 jul
Date

28 Serbia

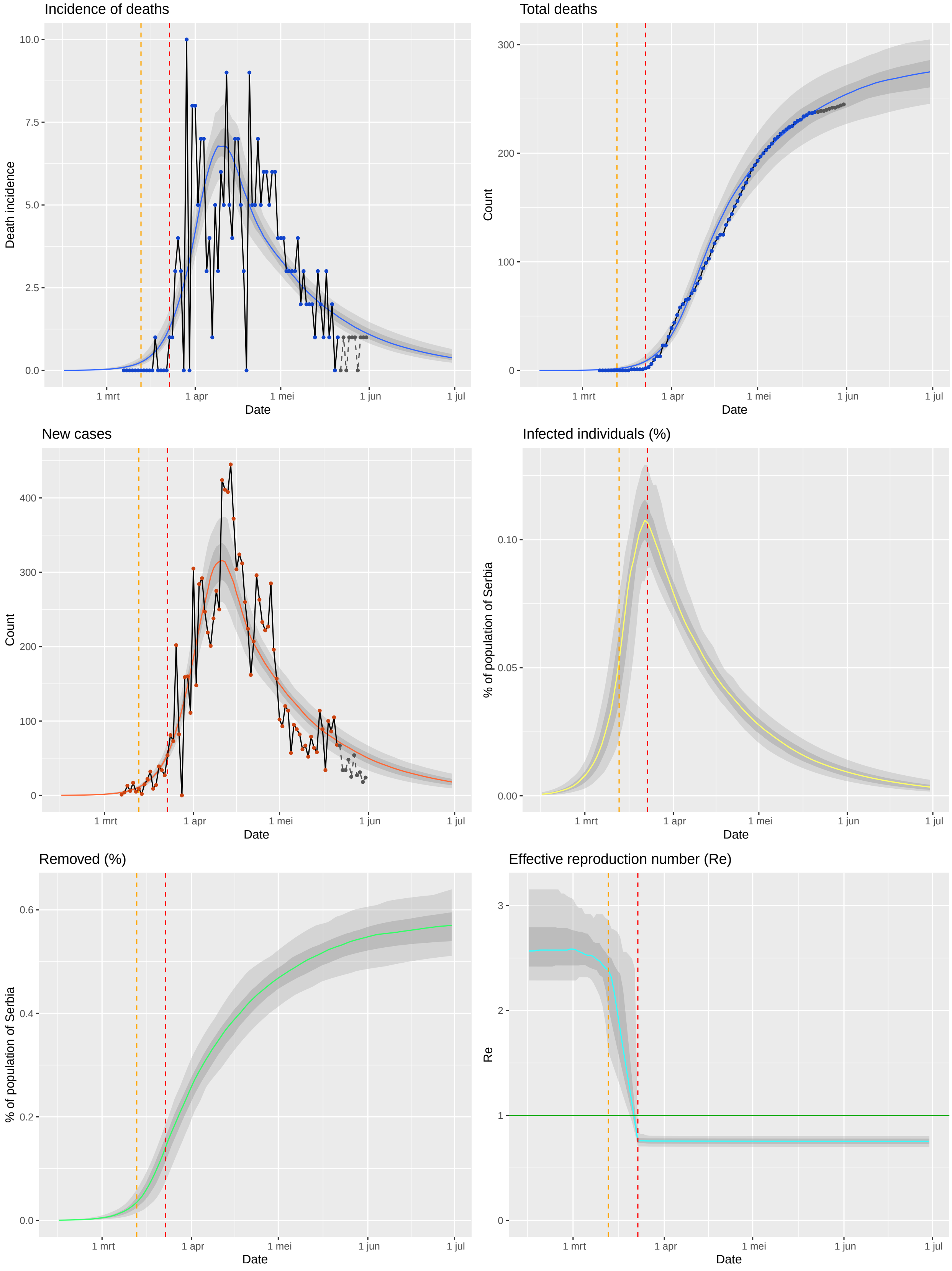

Incidence of deaths
Death incidence
10.0
7.5
5.0
2.5
0.0
1 mrt
1 apr
1 mei
1 jun
1 jul
Date
Total deaths
Count
300
200
100
0
1 mrt
1 apr
1 mei
1 jun
1 jul
Date
New cases
Count
400
300
200
100
0
1 mrt
1 apr
1 mei
1 jun
1 jul
Date
Infected individuals (%)
% of population of Serbia
0.10
0.05
0.00
1 mrt
1 apr
1 mei
1 jun
1 jul
Date
Removed (%)
% of population of Serbia
0.6
0.4
0.2
0.0
1 mrt
1 apr
1 mei
1 jun
1 jul
Date
Effective reproduction number (Re)
Re
3
2
1
0
1 mrt
1 apr
1 mei
1 jun
1 jul
Date

29 Slovakia

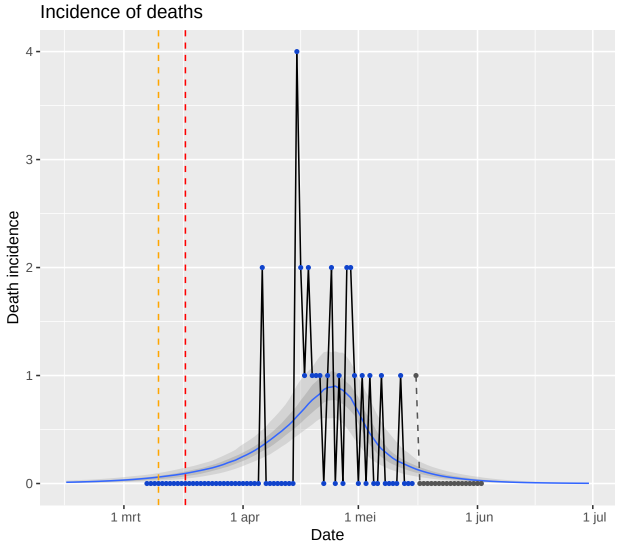
Incidence of deaths
Death incidence
0
1
2
3
4
1 mrt
1 apr
1 mei
1 jun
1 jul
Date

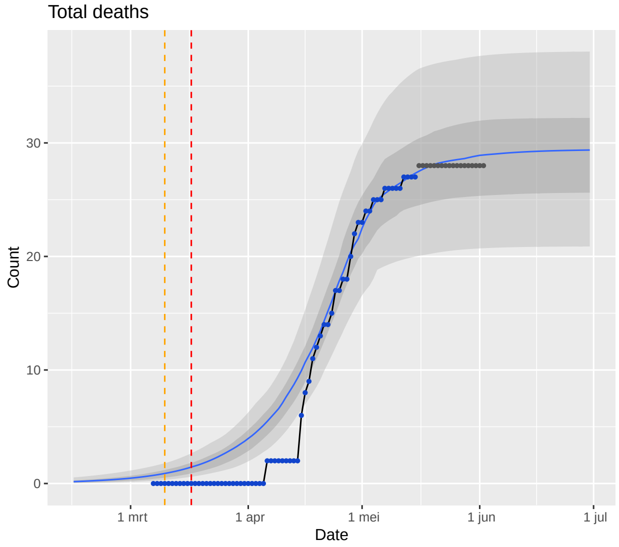
Total deaths
Count
0
10
20
30
1 mrt
1 apr
1 mei
1 jun
1 jul
Date

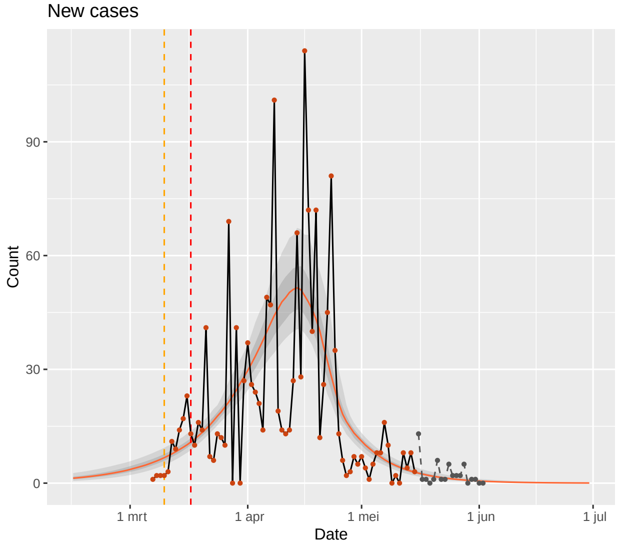
New cases
Count
0
30
60
90
1 mrt
1 apr
1 mei
1 jun
1 jul
Date

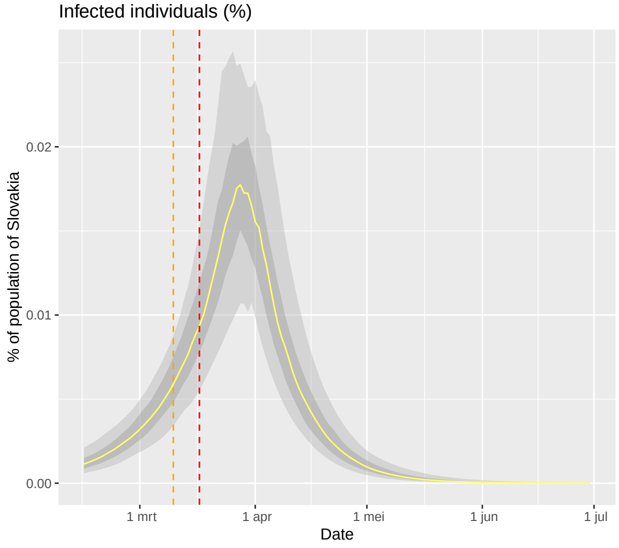
Infected individuals (%)
% of population of Slovakia
0.00
0.01
0.02
1 mrt
1 apr
1 mei
1 jun
1 jul
Date

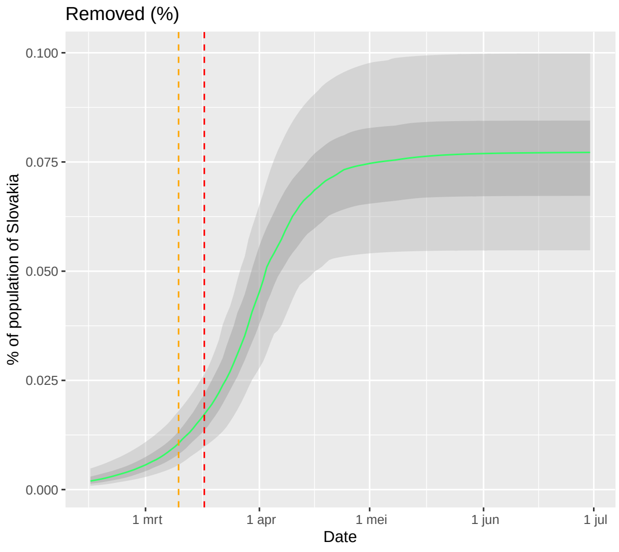
Removed (%)
% of population of Slovakia
0.000
0.025
0.050
0.075
0.100
1 mrt
1 apr
1 mei
1 jun
1 jul
Date

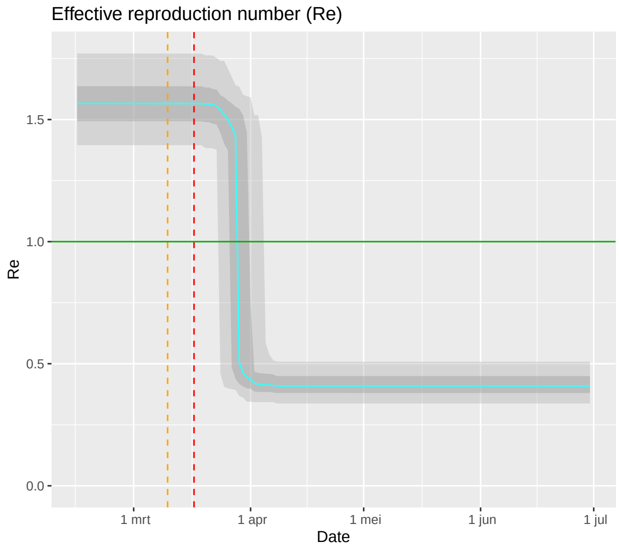
Effective reproduction number (Re)
Re
0.0
0.5
1.0
1.5
1 mrt
1 apr
1 mei
1 jun
1 jul
Date

30 Slovenia

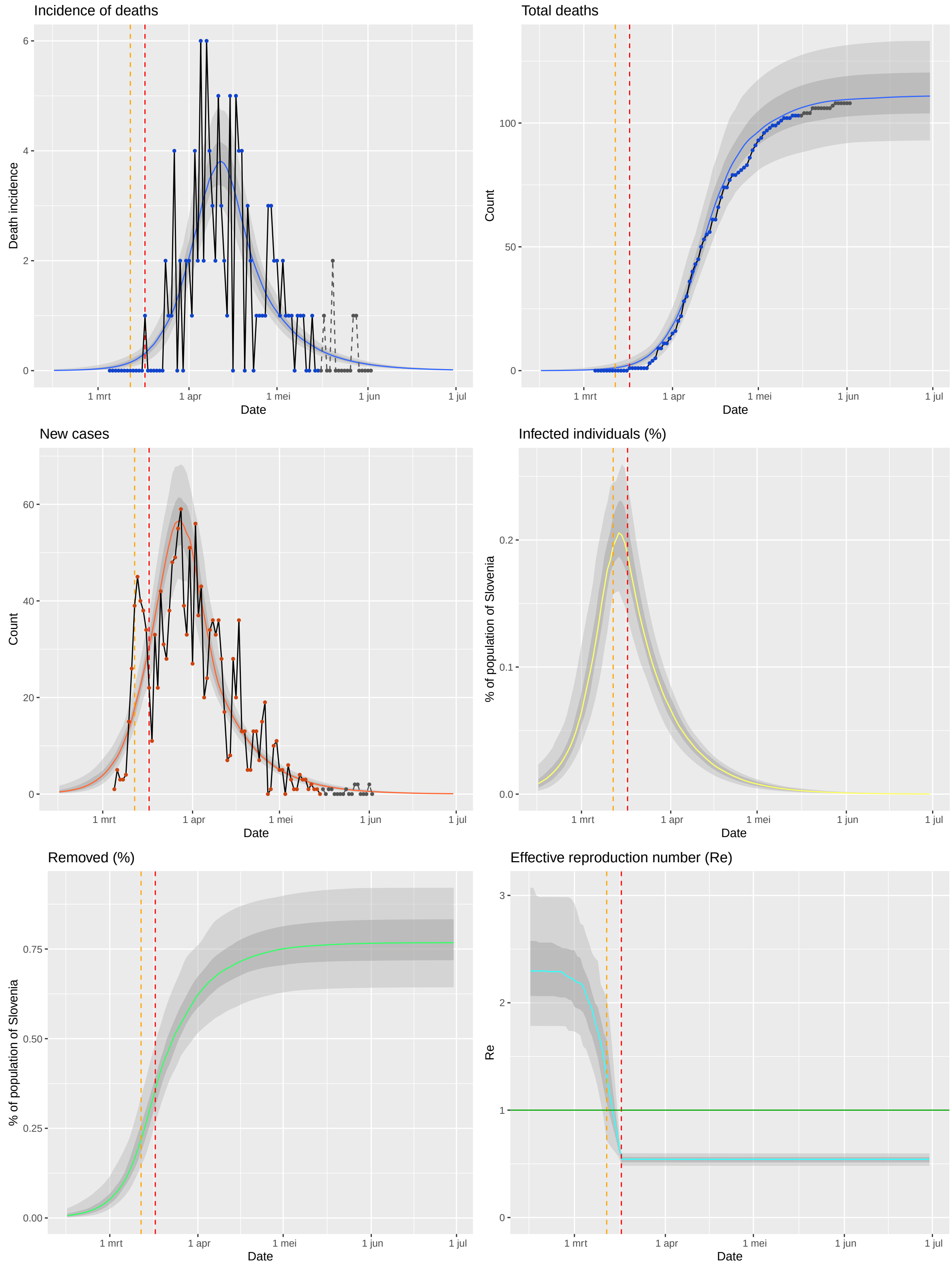
Incidence of deaths
Death incidence
0
2
4
6
1 mrt
1 apr
1 mei
1 jun
1 jul
Date
Total deaths
Count
0
50
100
1 mrt
1 apr
1 mei
1 jun
1 jul
Date
New cases
Count
0
20
40
60
1 mrt
1 apr
1 mei
1 jun
1 jul
Date
Infected individuals (%)
% of population of Slovenia
0.0
0.1
0.2
1 mrt
1 apr
1 mei
1 jun
1 jul
Date
Removed (%)
% of population of Slovenia
0.00
0.25
0.50
0.75
1 mrt
1 apr
1 mei
1 jun
1 jul
Date
Effective reproduction number (Re)
Re
0
1
2
3
1 mrt
1 apr
1 mei
1 jun
1 jul
Date

31 Spain

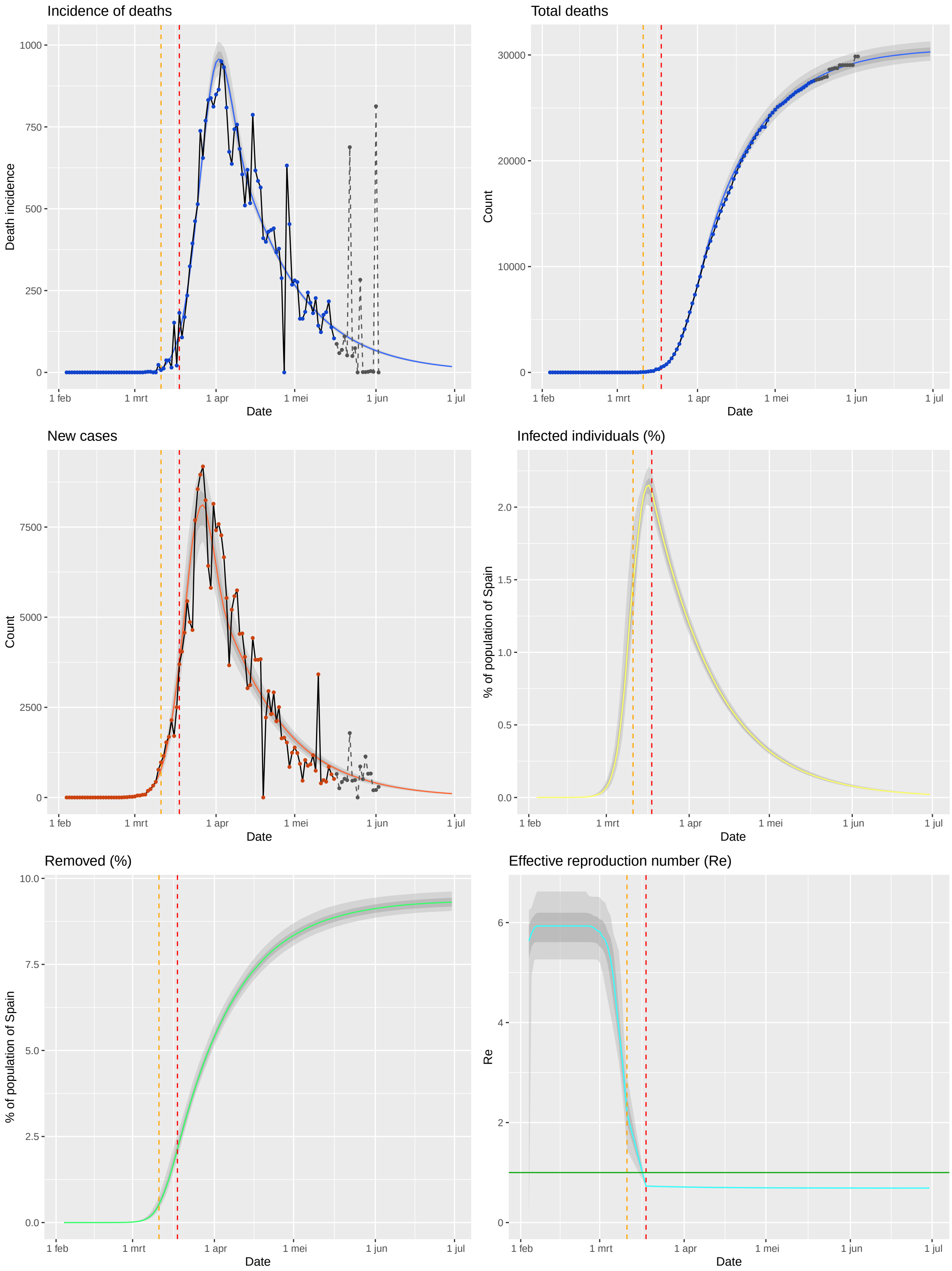

Incidence of deaths
Death incidence
1000
750
500
250
0
1 feb
1 mrt
1 apr
1 mei
1 jun
1 jul
Date
Total deaths
Count
30000
20000
10000
0
New cases
Count
7500
5000
2500
0
Infected individuals (%)
% of population of Spain
2.0
1.5
1.0
0.5
0.0
Removed (%)
% of population of Spain
10.0
7.5
5.0
2.5
0.0
Effective reproduction number (Re)
Re
6
4
2
0

32 Sweden

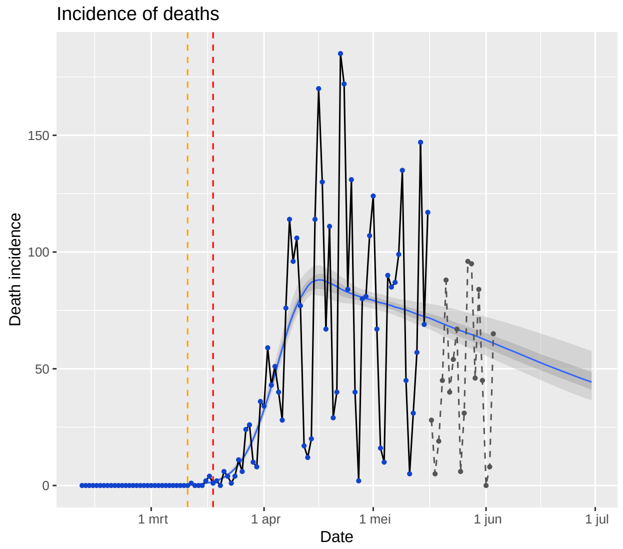

Incidence of deaths
Death incidence
0
50
100
150
1 mrt
1 apr
1 mei
1 jun
1 jul
Date

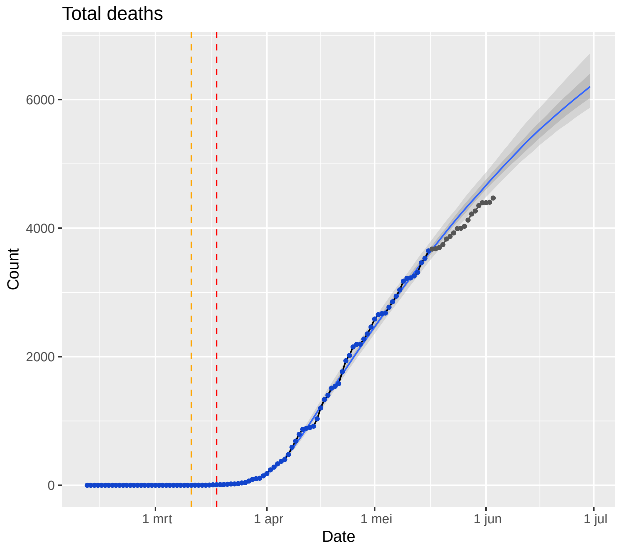

Total deaths
Count
0
2000
4000
6000
1 mrt
1 apr
1 mei
1 jun
1 jul
Date

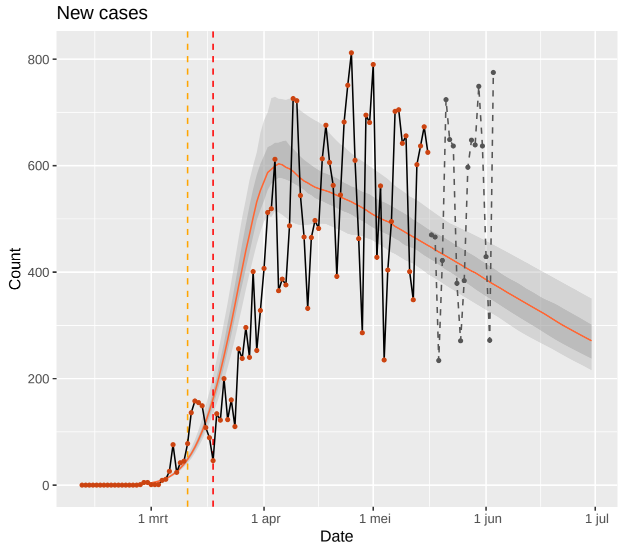

New cases
Count
0
200
400
600
800
1 mrt
1 apr
1 mei
1 jun
1 jul
Date

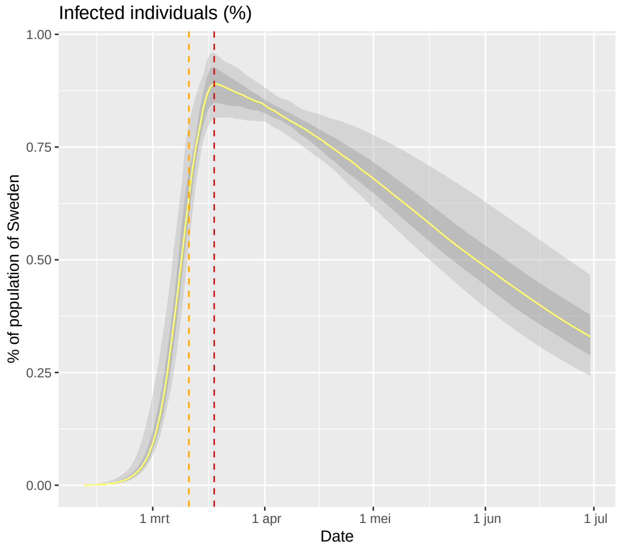

Infected individuals (%)
% of population of Sweden
0.00
0.25
0.50
0.75
1.00
1 mrt
1 apr
1 mei
1 jun
1 jul
Date

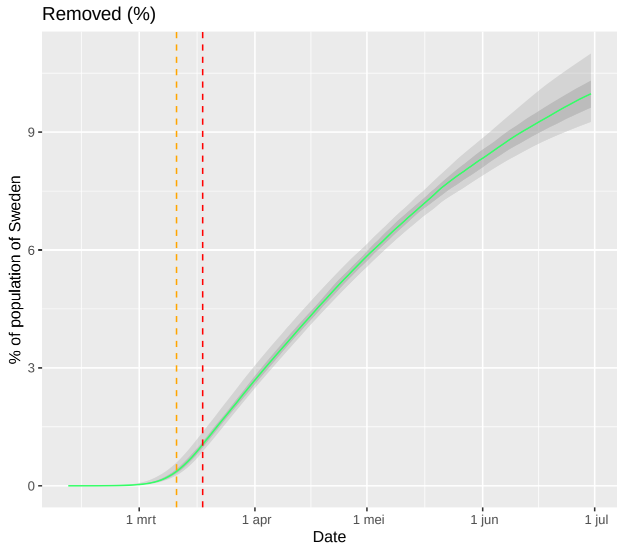

Removed (%)
% of population of Sweden
0
3
6
9
1 mrt
1 apr
1 mei
1 jun
1 jul
Date

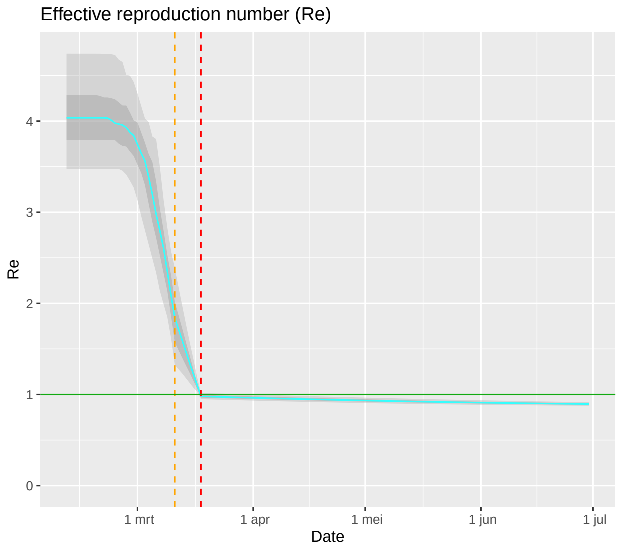

Effective reproduction number (Re)
Re
0
1
2
3
4
1 mrt
1 apr
1 mei
1 jun
1 jul
Date

33 Switzerland

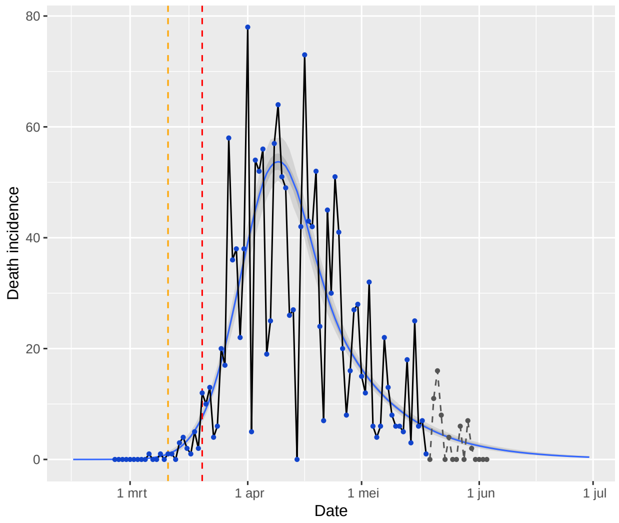

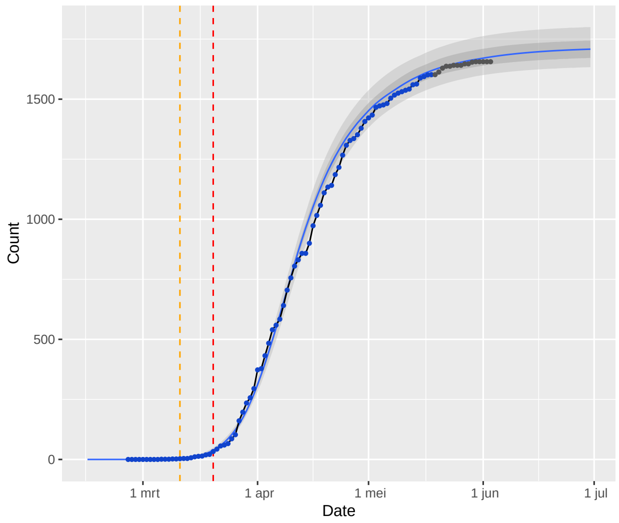

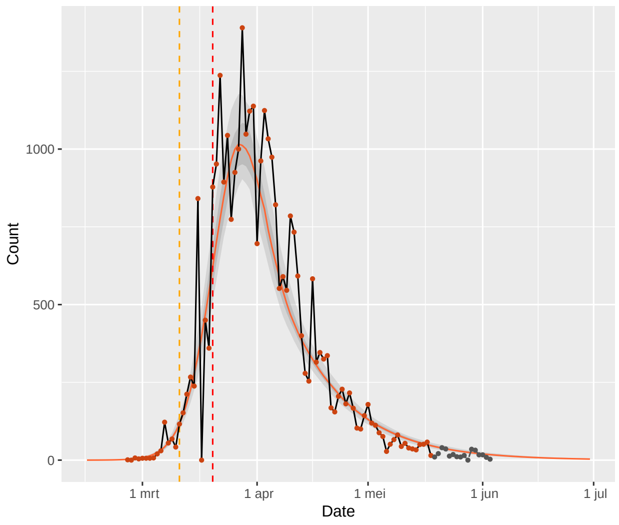

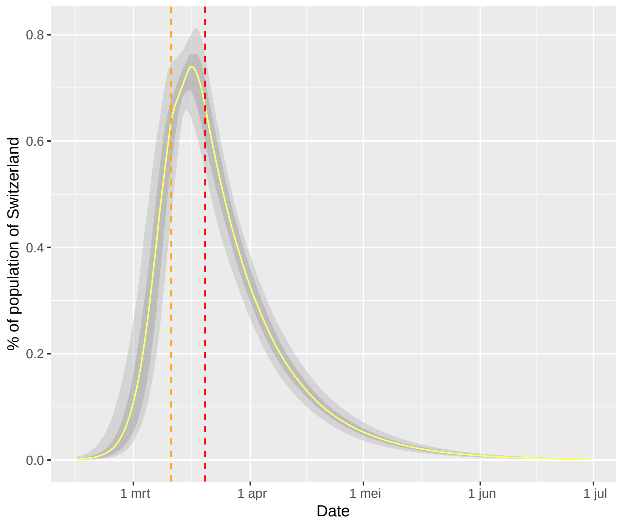

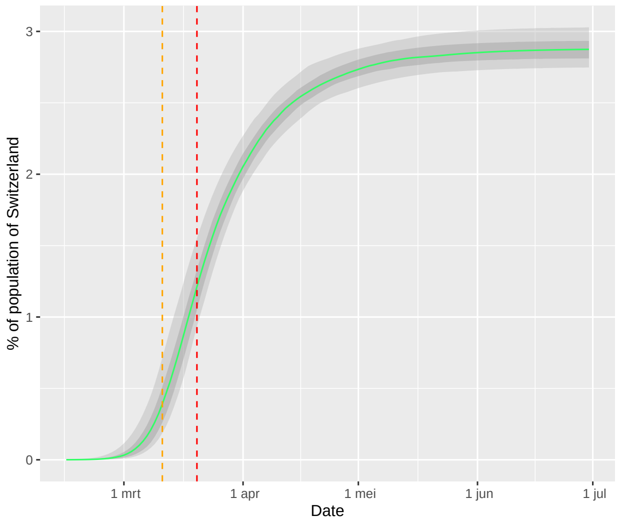

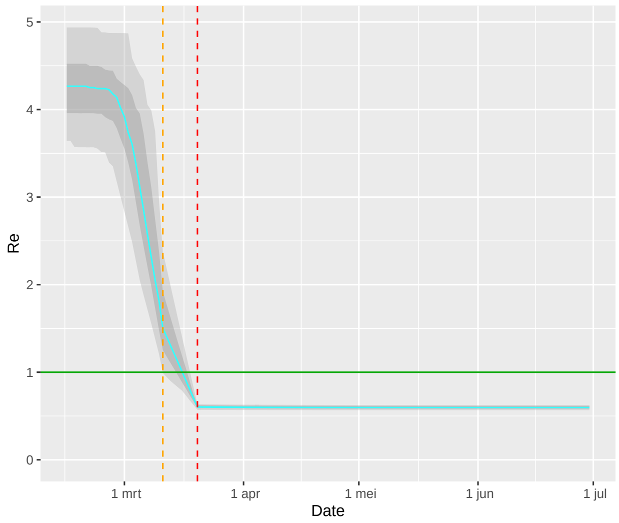

34 United Kingdom

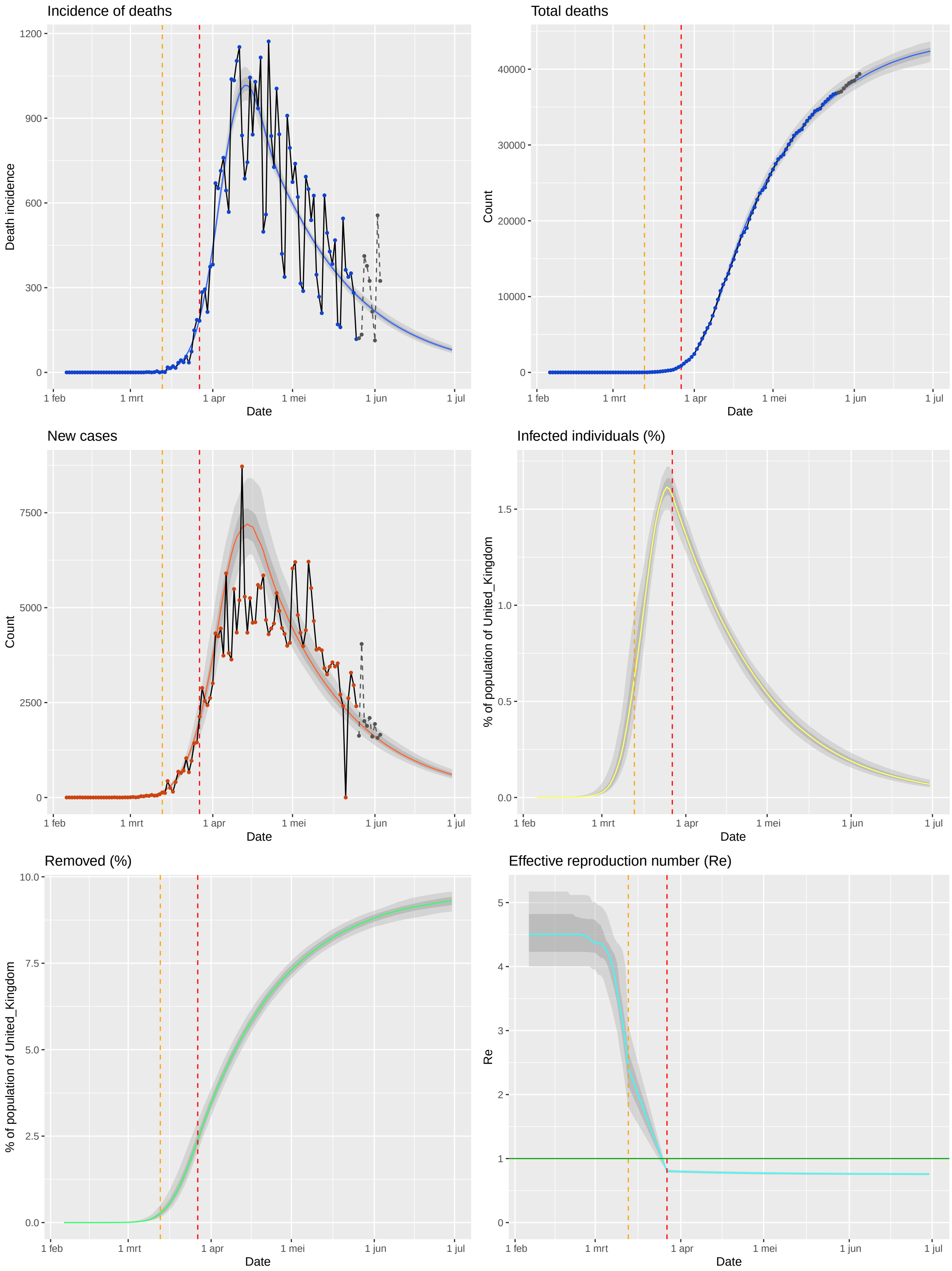

35 United States of America

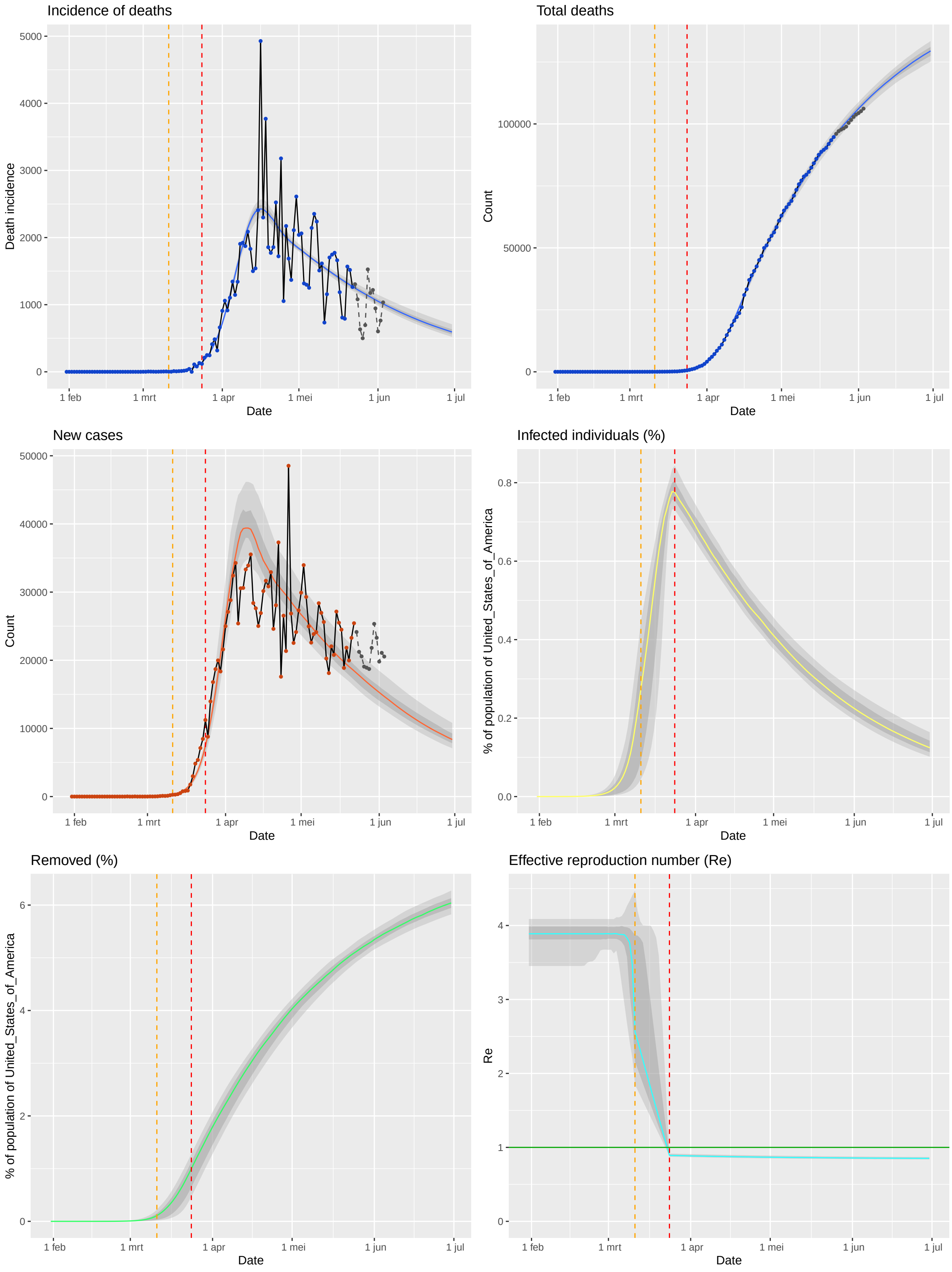

Supplement: Supplementary file 4 — Additional file 4. Model predictions per country. [file 12889_2021_10676_MOESM4_ESM.pdf]
